# Inferring microbiota functions from taxonomic genes: a review

**DOI:** 10.1093/gigascience/giab090

**Published:** 2022-01-12

**Authors:** Christophe Djemiel, Pierre-Alain Maron, Sébastien Terrat, Samuel Dequiedt, Aurélien Cottin, Lionel Ranjard

**Affiliations:** Agroécologie, AgroSup Dijon, INRAE, Université de Bourgogne, Université de Bourgogne Franche-Comté, F-21000 Dijon, France; Agroécologie, AgroSup Dijon, INRAE, Université de Bourgogne, Université de Bourgogne Franche-Comté, F-21000 Dijon, France; Agroécologie, AgroSup Dijon, INRAE, Université de Bourgogne, Université de Bourgogne Franche-Comté, F-21000 Dijon, France; Agroécologie, AgroSup Dijon, INRAE, Université de Bourgogne, Université de Bourgogne Franche-Comté, F-21000 Dijon, France; Agroécologie, AgroSup Dijon, INRAE, Université de Bourgogne, Université de Bourgogne Franche-Comté, F-21000 Dijon, France; Agroécologie, AgroSup Dijon, INRAE, Université de Bourgogne, Université de Bourgogne Franche-Comté, F-21000 Dijon, France

**Keywords:** microbiota, metabarcoding, taxonomy, functional inference, ecological traits, soil

## Abstract

Deciphering microbiota functions is crucial to predict ecosystem sustainability in response to global change. High-throughput sequencing at the individual or community level has revolutionized our understanding of microbial ecology, leading to the big data era and improving our ability to link microbial diversity with microbial functions. Recent advances in bioinformatics have been key for developing functional prediction tools based on DNA metabarcoding data and using taxonomic gene information. This cheaper approach in every aspect serves as an alternative to shotgun sequencing. Although these tools are increasingly used by ecologists, an objective evaluation of their modularity, portability, and robustness is lacking. Here, we reviewed 100 scientific papers on functional inference and ecological trait assignment to rank the advantages, specificities, and drawbacks of these tools, using a scientific benchmarking. To date, inference tools have been mainly devoted to bacterial functions, and ecological trait assignment tools, to fungal functions. A major limitation is the lack of reference genomes—compared with the human microbiota—especially for complex ecosystems such as soils. Finally, we explore applied research prospects. These tools are promising and already provide relevant information on ecosystem functioning, but standardized indicators and corresponding repositories are still lacking that would enable them to be used for operational diagnosis.

## Background

Microorganisms are present in all habitats on Earth and are essential for animals, plants, and therefore for the sustainability of human activities [1]. The extraordinary diversity of microbial communities plays an essential role in the various biogeochemical cycles, allows aquatic and terrestrial ecosystems to function properly, and ensures their ability to provide ecological services (e.g., soil structuring, organic matter renewal, nutrient recycling, pollution control, regulation/barrier to pathogens, or even plant productivity) [2–4]. Their fabulous capacity to adapt to different environmental stresses over time is now well known, and the regulation process of their diversity is better and better deciphered. Despite these tremendous improvements in the approaches targeting indigenous microbiotas, our understanding of the link between microbes and their associated functions remains limited [5]. A workshop hosted by the British Ecological Society's Microbial Ecology Special Interest Group (June 2016) recently identified 50 important research questions in microbial ecology. One of the main ones was “What methods can we use to marry microbial diversity with function; how do we link transcriptomics, proteomics and metabolomics?” [6]. This sums up the future challenges facing the scientific community when it comes to improving our understanding of the regulation of the microbiome diversity and functions [7].

**Figure 1:**
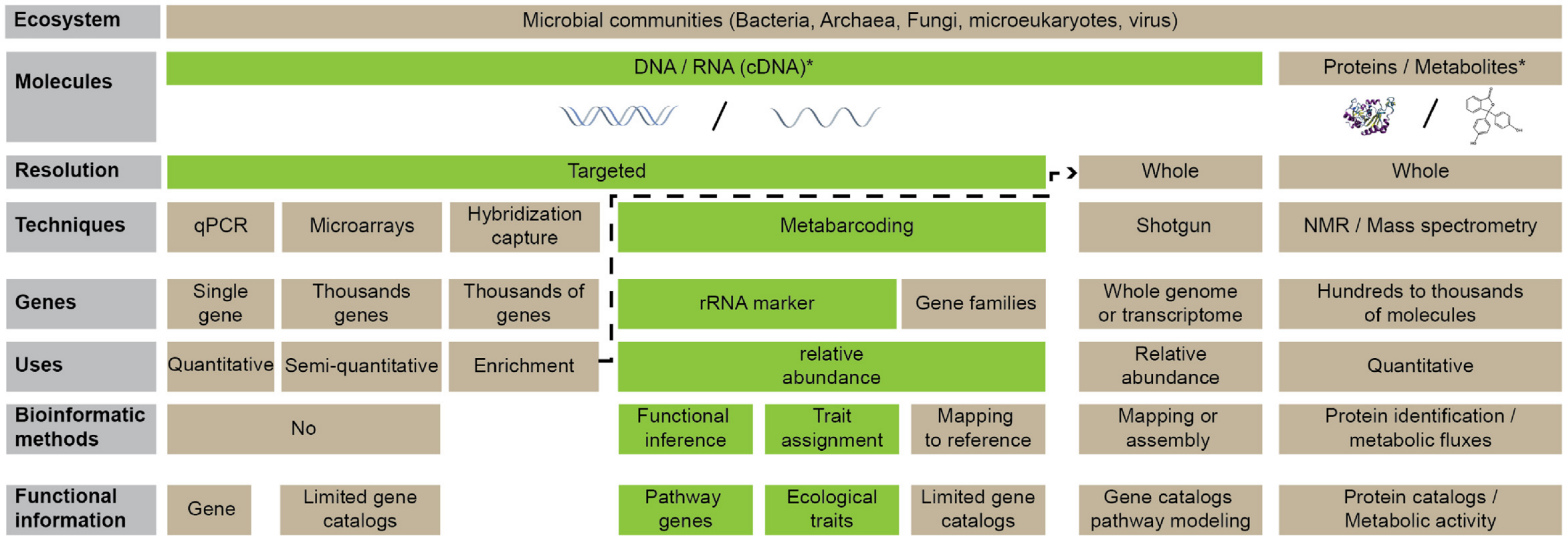
Schematic diagram of the various strategies available for exploring the functional diversity of the microbiota. Green frames indicate metabarcoding approaches for retrieving putative functions from taxonomic genes by functional inference and ecological trait assignment. cDNA: complementary DNA; NMR: nuclear magnetic resonance; rRNA: ribosomal RNA.

Microbial functions can be characterized from genomic, proteomic, or metabolic data (Fig. 1) [8–10]. Considering genomics, quantitative PCR (qPCR) and microarrays were the first technologies used to describe functional genes or taxa from complex environmental samples [11]. Initially designed to determine the absolute copy number of a single given gene, the latest technical advances can analyze thousands of combinations of samples and targets in parallel [12]. Standardized methods even make it possible to quantify genes of interest (e.g., involved in biogeochemical cycles or pesticide degradation) to estimate soil quality [13]. DNA microarrays were the first high-throughput technologies giving access to gene expression profiles at the individual or community levels [11, 14]. There exist different kinds of microarrays (e.g., PhyloChip, GeoChip, PathoChip, StressChip, CAZyChip). They provide a snapshot of microbial diversity (bacteria, fungi, viruses) and/or of the functional genes present in a given sample (e.g., genes coding for enzymes involved in polysaccharide degradation) [15–18]. Some of these microarrays have become diagnostic tools in many fields, in particular for targeting viruses, bacterial or fungal pathogens, or harmful organisms [19]. More recent and cheaper, various high-throughput sequencing (HTS) alternatives have been developed to explore microbial communities (Fig. 1) [20]. Genome and metagenome sequencing have changed the microbial ecology field: thanks to genome sequencing and meta-omics approaches, gene catalogs can be assessed, and new microorganisms can be discovered [21, 22].

For example, by implementing a metabarcoding approach, microbial ecologists were initially enthusiastic about such huge taxonomic information but quickly pointed out the lack of associated functional information [22]. Taxonomic profiles can indeed change to varying degrees among samples, and predicting to what extent these changes affect the overall functional capacity of the community has remained a technical and scientific challenge to date [6, 23, 24]. Metabarcoding may well be used to directly target functional genes and classify them by taxonomic group, but applications remain limited to a few families [25–29]. In the face of these limitations, 2 solutions have emerged to indirectly obtain functional information from taxonomic profiles, i.e., (i) functional inference, and (ii) ecological trait assignment, using (meta)genome and microbiome big data (Fig. 1). Functional inference predicts the putative functions (e.g., gene catalogs, metabolic pathways) of microbial communities, while ecological trait assignment directly retrieves a trait common to all taxa by linking taxonomic names with a dedicated database. The major difference between these 2 solutions for obtaining functional information is that functional inference retrieves functions even for operational taxonomic units (OTUs) without a taxonomic name thanks to phylogenetic placement of sequences (taxonomic markers) in a reference tree and different evolutionary models.

Many bioinformatic tools have been developed since the first publication about a functional prediction tool using metabarcoding data. To date, only 1 review has addressed functional inference tools; it is focused on aquaculture and on a limited subset of all the tools available to predict functions from 16S ribosomal DNA (rDNA) metabarcoding datasets [30]. Therefore, in the present context where new solutions are proposed regularly to predict putative function profiles, the state of the art needs to be scrutinized more exhaustively to build a scientific and technical benchmark. More precisely, we provide a detailed description of each tool and evaluate their advantages, specificities, and drawbacks by paying special attention to their methods, modularity, portability, and robustness. One of the main objectives of this review is to provide a rationale on the use of the different tools currently available for prokaryote and fungal communities and draw perspectives, with a few suggestions to enhance their usefulness in microbial ecology. Finally, we illustrate the application of these methods with studies focusing on the soil environment. The choice of this particular system is justified by the fact that it is the most diverse and complex one in terms of microbial diversity, ecology, and functional reservoir [4, 31]; therefore, it represents the most challenging environmental matrix for linking diversity and functions. We believe that this work will help scientists working on microbial communities make choices to best take advantage of their high amount of microbial data. This work also shows that although those approaches are promising, they still need improvements to make them operational tools for microbial diagnosis. Repositories using standardized and robust metrics are still lacking when it comes to interpreting the results.

## Historical and Recent Increase of Microbial Datasets

**Figure 2:**
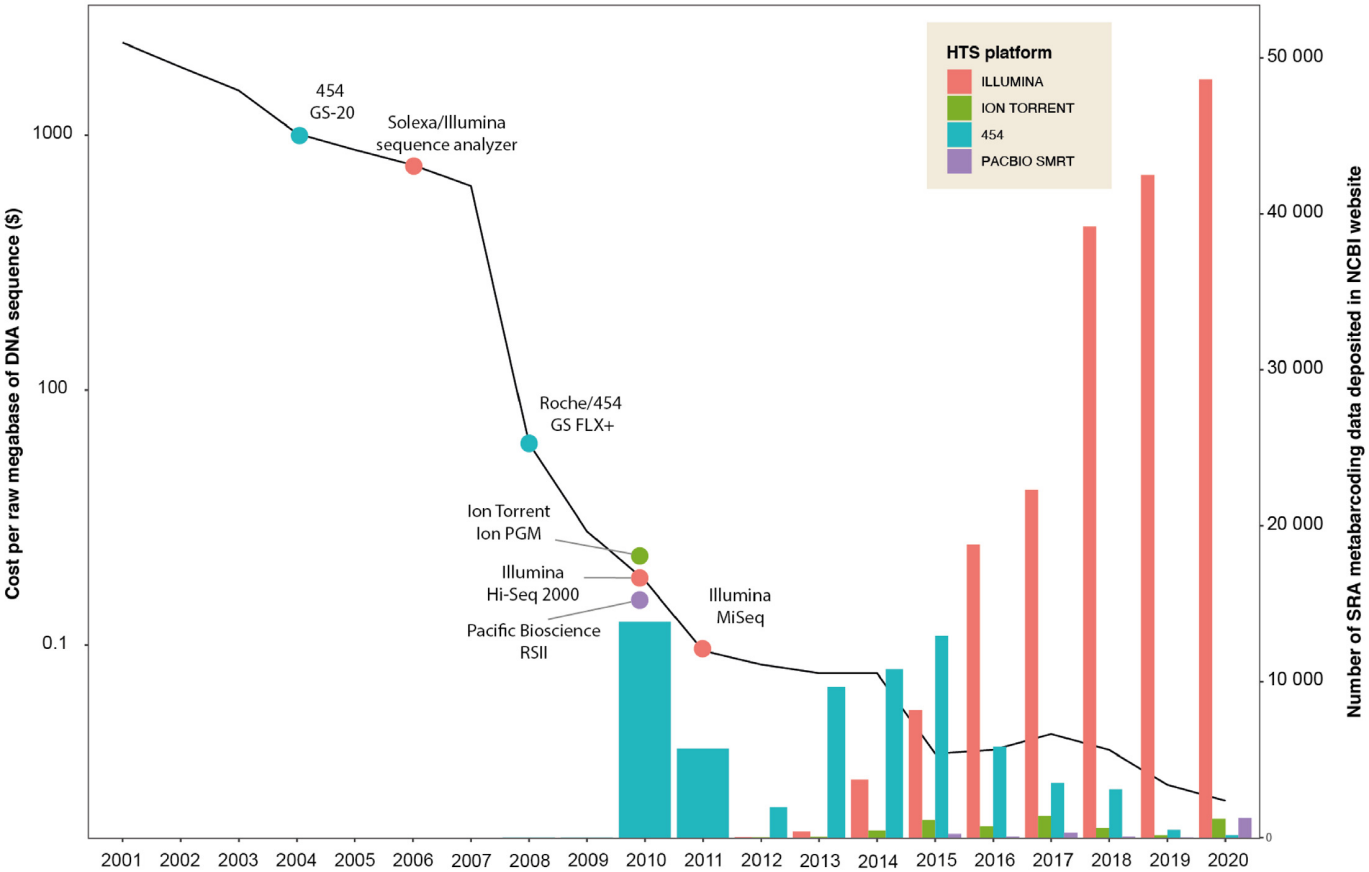
Evolution of costs (dollars) per raw megabase of DNA sequence (black line with logarithmic scale), and evolution of the number of SRA metabarcoding data deposited in the NCBI website. The data used to draw this figure are described in Additional File 1, section Figure 2.

The emergence of HTS in the mid 2000s generated a huge volume of data, leading to a revolution in our way of describing biodiversity. This rise of microbial data can be directly linked to the improvement of HTS technologies, concomitantly with a tremendous decrease in sequencing costs (Fig. 2). This was reflected, with a small time lag, by an increase in the number of sequence read archives (SRAs) linked to metabarcoding data deposited on the NCBI website (Fig. 2).

**Figure 3:**
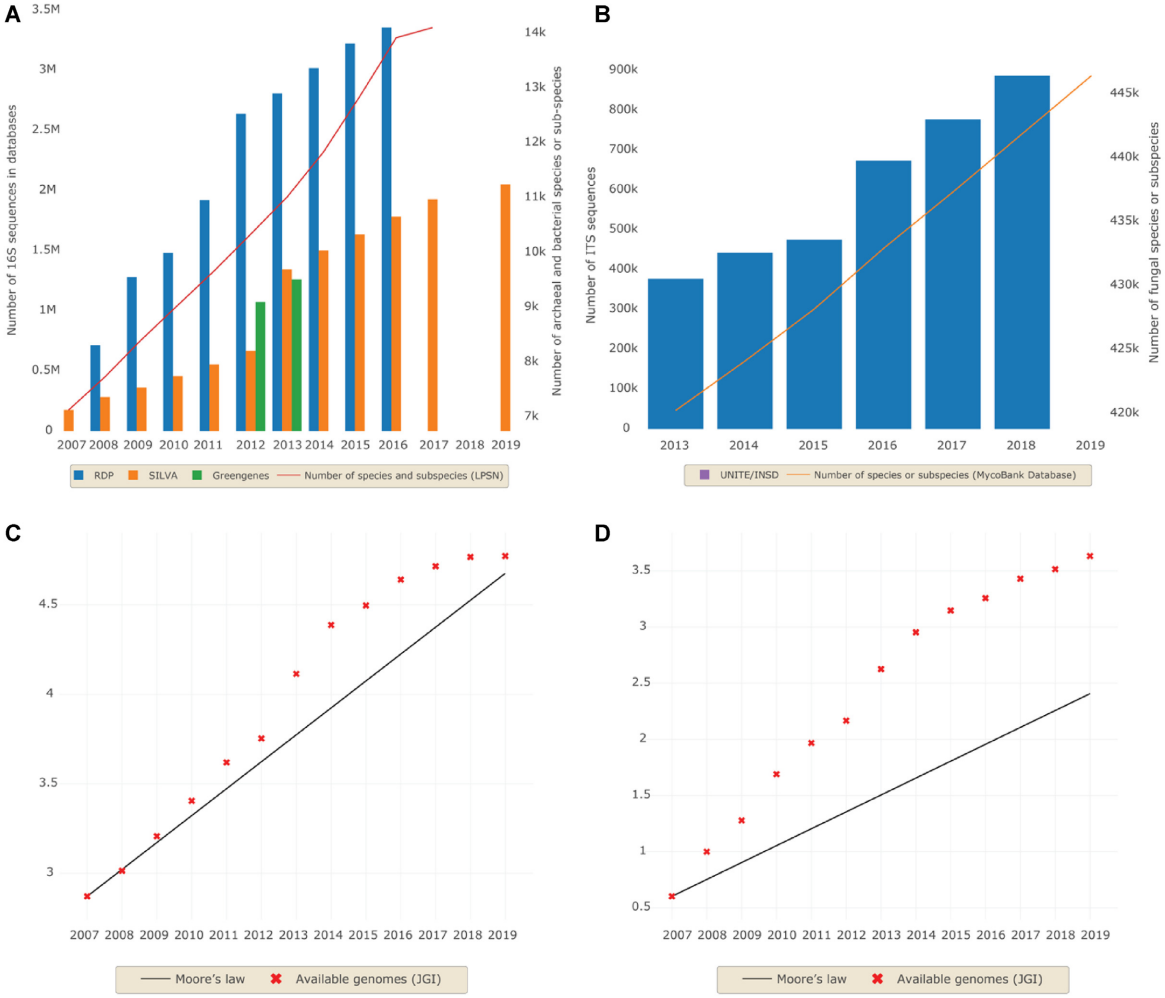
Annual cumulative growth of databases in terms of bacterial/archaeal (A) and fungal (B) sequences, and species/subspecies deposited per year. Comparison of the annual cumulative growth of bacterial/archaeal (C) and fungal (D) genomes compared to simulations of Moore's law. The plot is in logarithmic scale. Three databases were compared for 16S rRNA gene sequences: RDP (blue), SILVA (orange), and Greengenes (green). Information is based on the List of Prokaryotic names with Standing in Nomenclature (LPSN [34, 35]) website for bacterial and archaeal species, and on the MycoBank database for fungal species [36, 37]. Information about the bacterial, archaeal, and fungal genomes is based on the Genome OnLine Database (GOLD) [38].

Thanks to the contribution of ecologists, microbiologists, taxonomists, and computer scientists, the databases are continuously enriched and are key to enhance our knowledge about the description and determinism of environmental and human microbiotas [32, 33]. For example, the 16S rDNA sequence data available to analyze bacterial/archaeal diversity were multiplied by 4 and 10 in the RDP and SILVA databases, respectively, between 2007 and 2019 (Fig. 3A). The trend is the same for fungal diversity, with a doubling of internal transcribed spacer (ITS) sequences in the UNITE/INSD database within the past 5 years (Fig. 3B). The 16S rDNA sequences are much more numerous than ITS sequences. However, there were 30 times more fungal species referenced than bacterial ones in 2017 (Fig. 3A and B). The numbers of microbial genomes available, in particular in the Joint Genome Institute (JGI) platform, have increased continuously, and they outpaced Moore's Law mostly from 2013 for bacteria and archaea (Fig. 3C and D).

**Figure 4:**
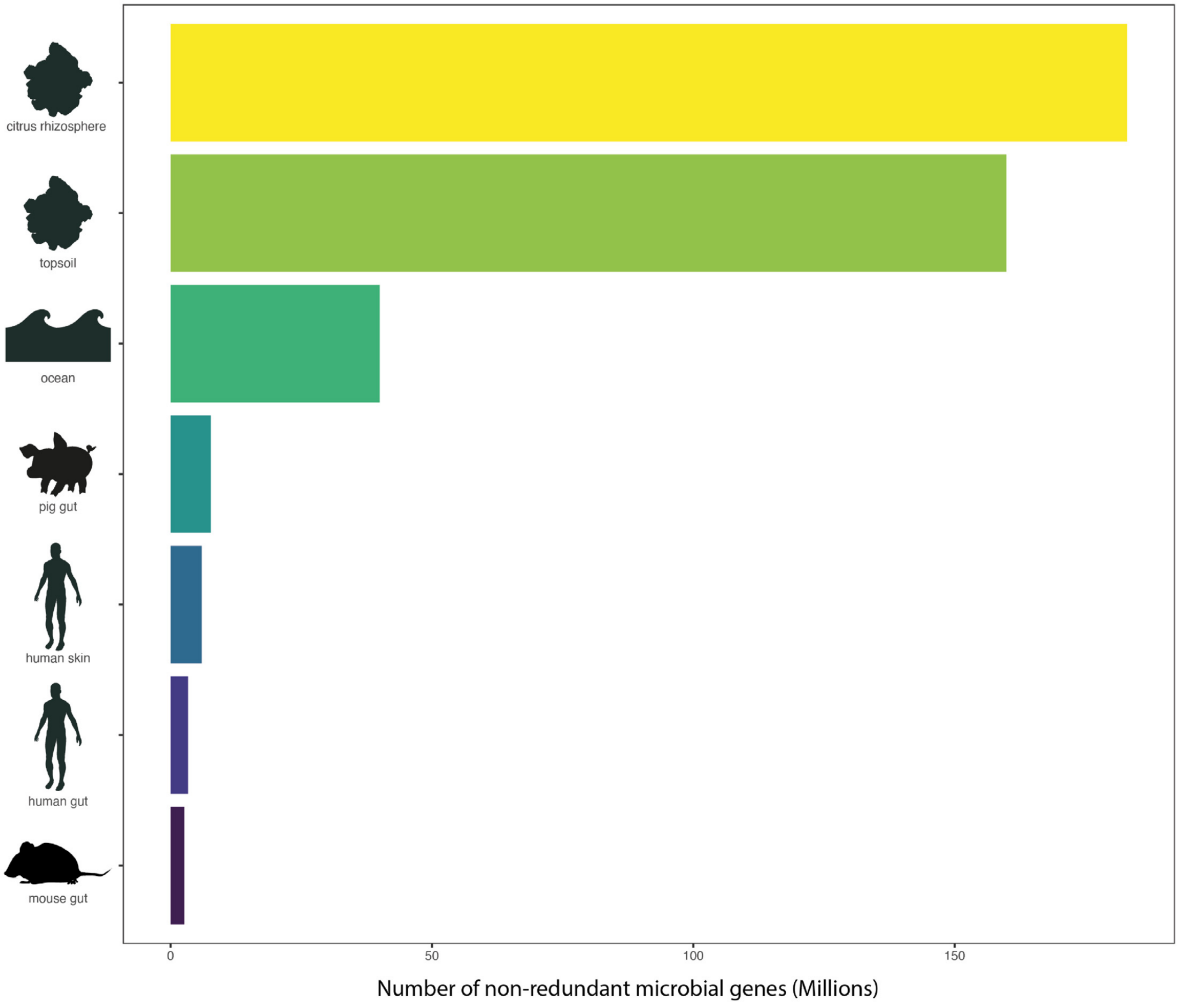
Global microbial gene catalogs from various ecosystems. The references are listed in Additional File 1.

The number of known microbial genes, enzymes, or metabolic pathways available in specialized databases has also considerably increased in the past few years [39–41]. Thousands of functional information files are currently accessible in the KEGG, CAZy, or MetaCyc databases (Table 1). A recent survey predicted the total global estimated bacterial and fungal functions based on KEGG Orthology (KO) to reach 35.5 and 3.2 million, respectively [42]. The authors also indicated that only a tiny fraction of these functions is known today, representing 0.02% and 0.14% for bacteria and fungi, respectively. Although the characterization of gene catalogs using metagenomic approaches was recently criticized [43], the number of non-redundant genes provides an overview of the potential functional reservoir available across various ecosystems [44]. The soil by far seems to harbor the largest pool of functions, followed by the marine, and then animal microbiomes (Fig. 4).

**Table 1:** Numbers of organisms, genes, enzymes, and metabolic pathways available in the CAZy, KEGG, and MetaCyc databases

Database	Organisms	Metabolic pathways	Enzymes/Genes
CAZy	Eukaryotes: 344; Bacteria: 20,421; Archaea: 413	NA	GH: 171; GT: 114; PL: 41; CE: 19; AA: 16
KEGG	Eukaryotes: 557; Bacteria: 6,317; Archaea: 344	547	KO groups: 24,402
MetaCyc	Total: 3,295	2,937	13,356

When possible, we detailed the number of organisms for the 3 domains of the tree of life. CAZy includes glycoside hydrolases (GH), glycosyl transferases (GT), carbohydrate esterases (CE), polysaccharide lyases (PL), and auxiliary activities (AA). CAZy: Carbohydrate-Active Enzymes; KEGG: Kyoto Encyclopedia of Genes and Genomes; KO: KEGG Orthology; MetaCyc: metabolic pathways and enzymes; NA: not applicable.

The rapid growth of available genomes is a unique opportunity to predict the putative microbial functions from metabarcoding data by linking taxonomic markers (i.e., rRNA gene amplicons) and their reference genomes or ecological traits. Therefore, the next section is devoted to the different tools and databases dedicated to functional inference and ecological trait assignment for bacterial and fungal communities.

## Overview of the Available Tools for Predicting the Potential Functions of the Microbiotas

HTS and the presently increasing collection of functional or ecological traits on a more regular and rigorous basis are promising cues for linking biodiversity and associated functions in the near future [24, 45]. In the literature, the term “function” is used in different ways depending on the study model, the time scale, or even the habitat [46–49]. The notion of function may refer to genes, enzymes, or metabolic pathways but may also represent ecological traits that bring together phenotypic and biochemical notions [50–52].

**Figure 5:**
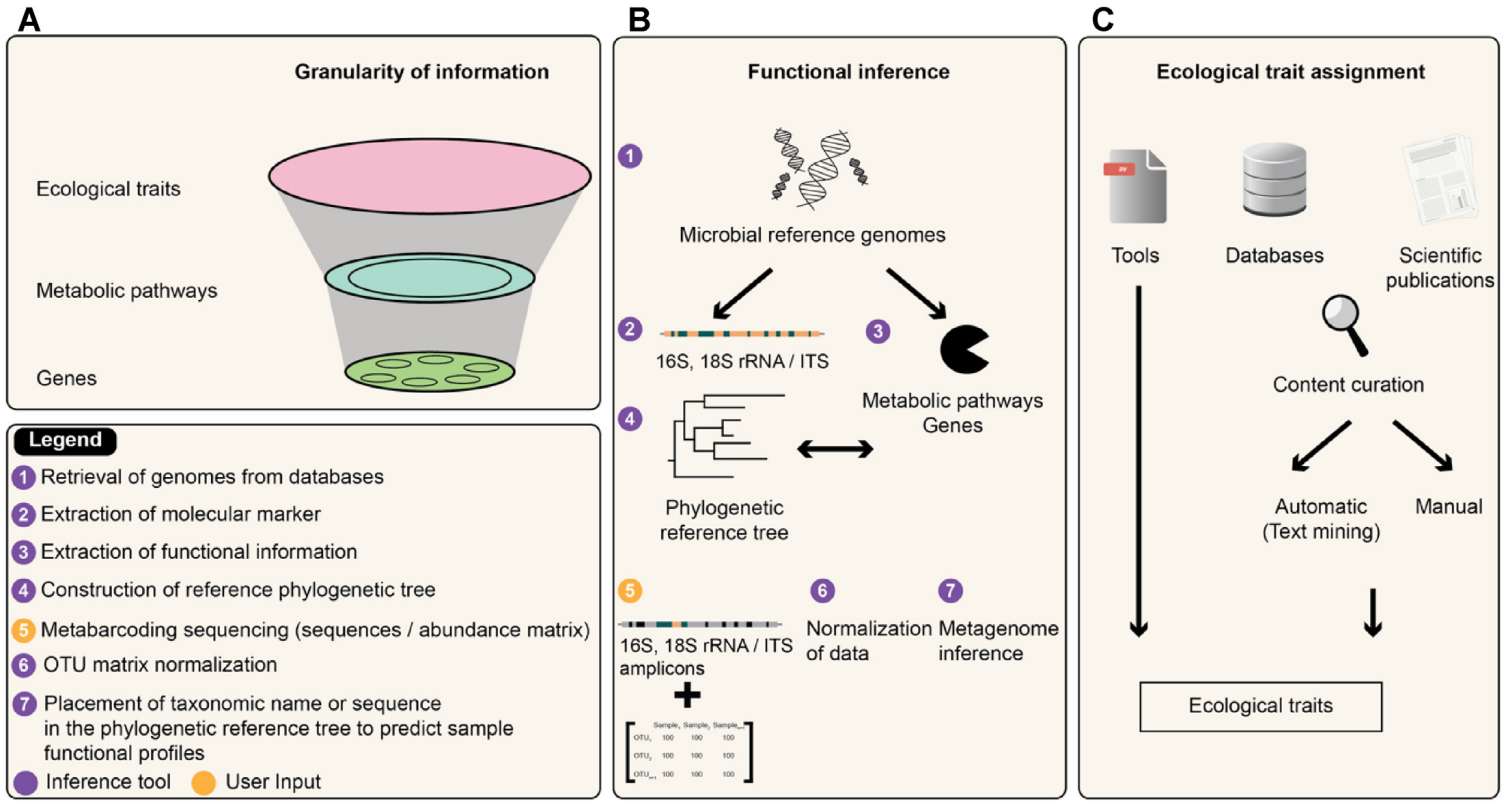
Diagram of the granularity of the data (A) that can be obtained by functional inference (B) or ecological trait assignment (C).

On the basis of the analysis of 20 papers since 2013, we classified the databases and tools according to the granularity of the results (Fig. 5A), from general information such as ecological traits to more detailed information such as genes or metabolic pathways (Fig. 5). The tools used to obtain fine results, i.e., at the metabolic pathway or gene levels for any taxonomic resolution, are known as functional inference tools (Fig. 5B). On the other hand, we grouped existing tools or databases under the term “ecological trait assignment” when functional information referred to phenotypic or ecological traits and was accessible only for a specific taxonomic rank (Fig. 5C). Indeed, there is a wealth of information often linked to ecological traits in published scientific articles, or of partially formatted metadata (i.e., partial taxonomy or data not linked to the ID of a taxonomic database) [53].

Tools or methods exist, known under the term “text mining,” to automatically collect data from various sources (e.g., a website, a document in pdf format) through automatic language processing (e.g., natural language processing) [54]. For example, @MInter [55] retrieves information related to microbial interactions from abstracts of articles thanks to a supervised machine learning model. Other tools are based on ontologies; i.e., they use a structured set of terms and concepts from a particular domain by specifying the relationships between these terms and their properties, and thus have a common reference for the use of a common vocabulary. For example, OntoBiotope [56] ontology in the food field retrieves the phenotypes and habitats of microbes from the literature based on the NCBI taxonomy. Another ontology exists, called Ontology of Microbial Phenotype [57]; it brings together a structured set of terms and concepts around microbial phenotypes, and specifies the relationships between these terms and their properties. Tools also based on machine learning such as ProTraits [58] can automatically annotate prokaryotic species on the basis of phenotypic or genomic data from scientific articles or online resources [59].

**Figure 6:**
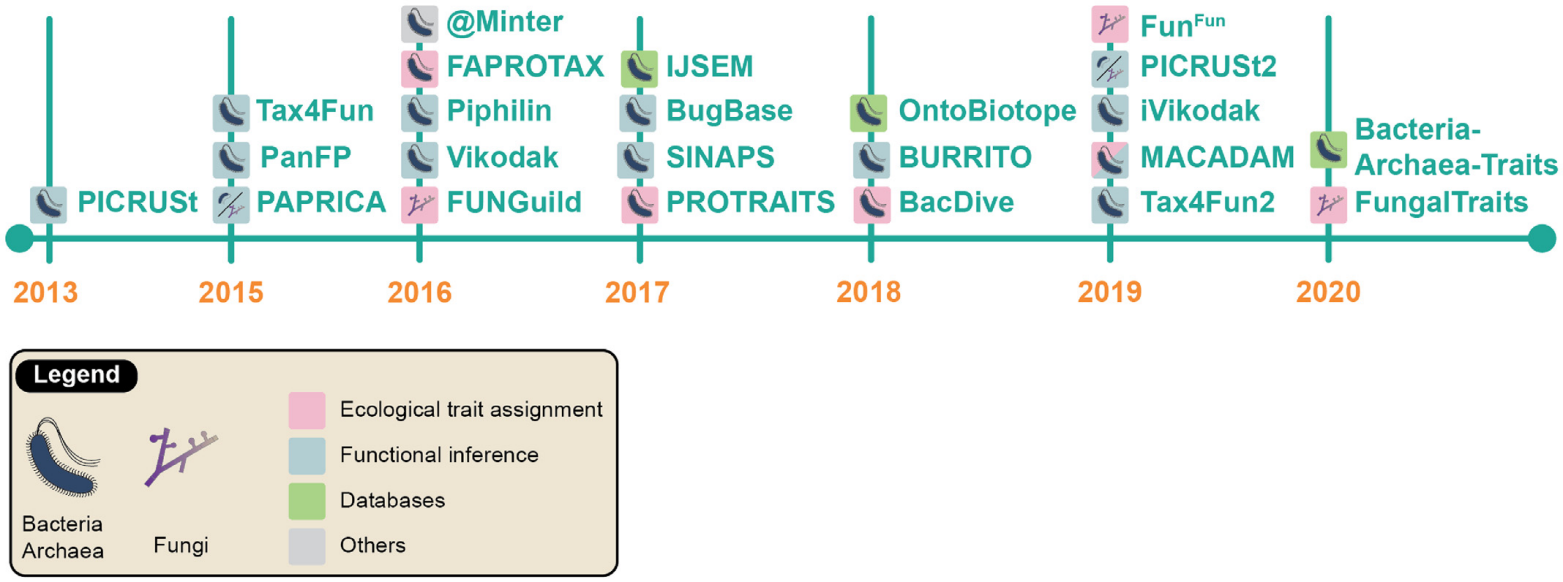
Timeline depicting the historical record of the major tools developed for functional inference or ecological trait assignment. The first version of the DEEMY database dates back to 1996; it was omitted for aesthetic reasons.

**Figure 7:**
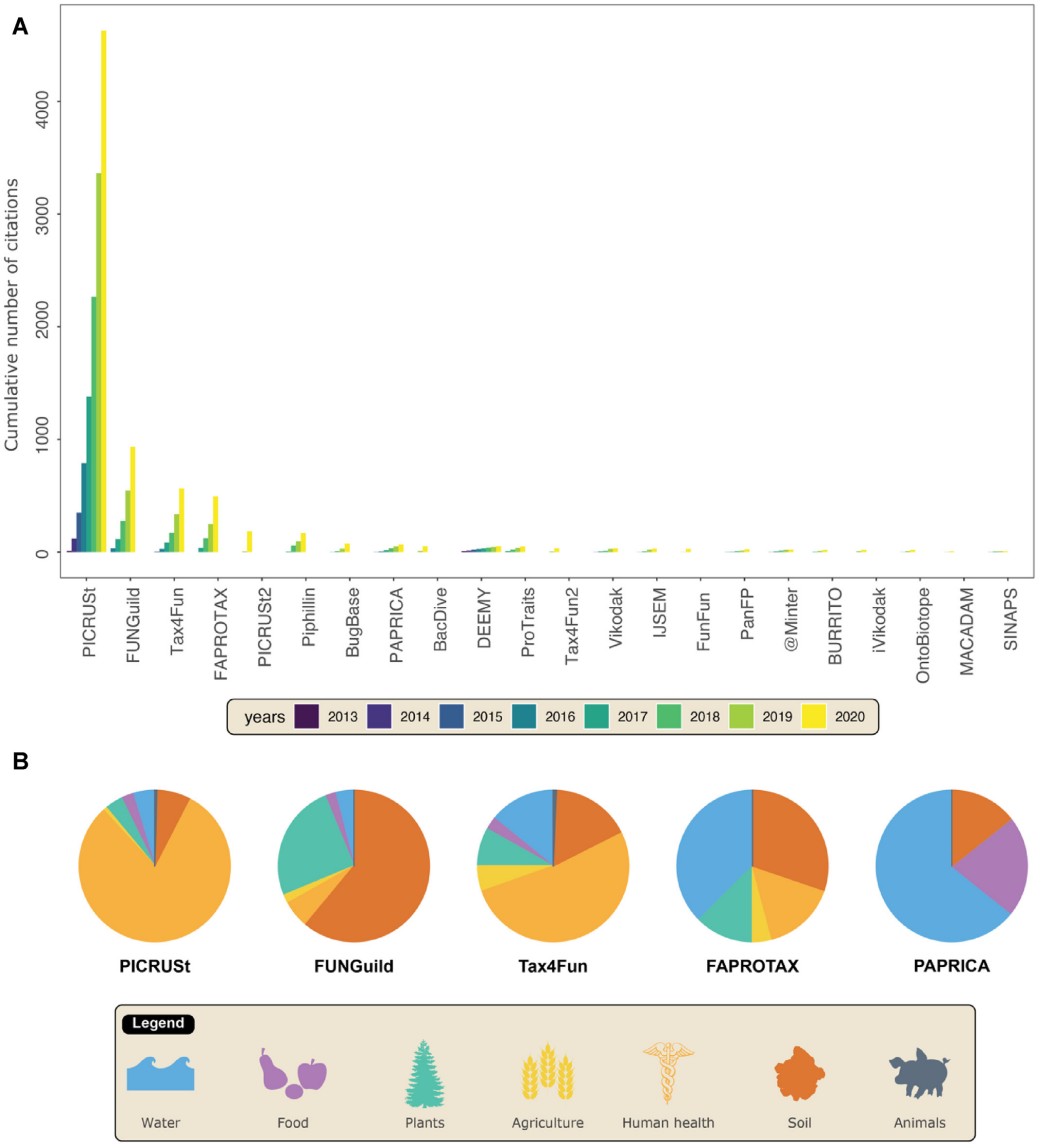
Annual cumulative number of citations of the major tools (A) and their scope (B). The keywords used for “scope” were retrieved from the titles and abstracts of the articles listed in Additional File 1.

To date, we have recorded ∼20 tools or databases that retrieve functional or ecological data from microbial taxonomic markers, with 2–4 developments per year (Fig. 6 and Table 2). The timeline shows that most of these tools (18 of 23 in total) are only dedicated to bacteria/archaea, 2 are dedicated to bacteria/archaea + fungi, and only 3 are specifically dedicated to fungal organisms. It is important to also underline that most of these tools are devoted to functional inference (13 of 23). The most cited tool is PICRUSt v1 [60], which continued to outrank all others with >4,000 citations in 2020. While FUNGuild [61], Tax4Fun v1 [62], or FAPROTAX [63] are moderately cited, with a few hundred citations, the others are much less so, with only a dozen citations (Fig. 7A). Interestingly, the articles citing functional inference and ecological trait assignment tools fall within the same scope as those for which they were initially developed (Fig 7B.): PICRUSt, FUNGuild, and PAPRICA are mainly cited in articles about human health, the soil, and marine environments, respectively.

**Table 2:** List of the functional inference tools, ecological trait assignment tools, and databases

Tool	Implementation	Targeted genes	Functional prediction	Approaches	Methods	Inputs used	Strengths and Specificities	Limitations
PanFP	Perl (recently Python)	16S rRNA	KO, Gene Ontology, Pfam, TIGRFAM	Functional inference	Builds a pangenome	NCBI taxonomy	Uses functional profile of the pangenome so could be less sensitive to horizontal gene transfer	Evolutionary models are not taken into account No confidence score generated Not yet available for microbial eukaryotes
PAPRICA	Python	16S/18S rRNA	MetaCyc ontology	Functional inference	Phylogenetic placement	Based on rDNA amplicon sequences	18S rRNA amplicons are taken into account Examples on the developer's blog	Errors may occur with sequence placement due to poor resolution of rRNA amplicons in some clades
PICRUSt	Python	16S rRNA	KO, KEGG Pathway, COG, CAZy	Functional inference	ASR (Wagner Parsimony, ACE ML, ACE REML, ACE PIC)	Greengenes taxonomy (18may2012 or v13.5/v13.8)	Evolutionary models are taken into account Confidence score generated (NSTI) Correction of OTU copy numbers	Based on specific taxonomy (Greengenes identifiers) KEGG database not updated since 2011 No pre-calculated table of fungal genomes available
PICRUSt2	Python/R	16S/18S rRNA/ITS	MetaCyc, KO, EC number, COG, Pfam, TIGRFAM	Functional inference	HSP (maximum parsimony, empirical probabilities, subtree averaging, SCP)	Based on rDNA amplicon sequences	Evolutionary models are taken into account Confidence score generated (NSTI) Twice as many KO scores Multiple HSP methods can be implemented (takes branch length weighting into account) 18S rRNA and ITS amplicons are taken into account Extensive documentation and active community	Errors may occur with sequence placement owing to poor resolution of rRNA amplicons in some clades
Piphillin	Web-based	16S rRNA	BioCyc, KEGG	Functional inference	Nearest-neighbor matching of 16S rRNA gene amplicons with genomes from reference databases	Based on rDNA amplicon sequences	Regular updates of functional databases rRNA copy number adjustment	Available online only Available for 16S rRNA only
SINAPS	USEARCH	16S rRNA	Trait annotation (e.g., energy metabolism, Gram-positive staining, presence of a flagellum)	Functional inference	Word counting	Greengenes, SILVA	Confidence is estimated by boostrapping Integrated to USEARCH tool	No peer-reviewed publication (bioRxiv preprint) Detailed explanation is missing (e.g., how was protrait input created?)
Tax4Fun	R package	16S rRNA	KO	Functional inference	Nearest-neighbor search based on a minimum 16S rRNA sequence similarity	SILVA taxonomy	Uses R (multiplatform) with pre-calculated files Confidence score generated (FTU and FSU) The algorithm could better predict poorly characterized taxa compared to approaches based on ASR with possible large distances in the tree, thanks to a minimum of similarity between sequences	Based on specific taxonomy (SILVA identifiers) KEGG database not updated since 2011
Tax4Fun2	R package	16S rRNA	KO	Functional inference	BLAST	Based on rDNA amplicon sequences	Algorithm with a minimal sequence similarity Uses R (multiplatform) with pre-calculated, highly memory-efficient platform-independent files Confidence score generated (FTU and FSU) KO update from 2018 Calculates the redundancy of specific functions directly Builds its own habitat-specific reference	Not yet available for microbial eukaryotes
Vikodak	Web-based (not longer available)	16S rRNA	KEGG pathway, EC number	Functional inference	Microbial co-existence patterns	RDP, SILVA	Pathway exclusion cut-off value is available to provide the minimum percentage of genes/enzymes belonging to a metabolic pathway required to consider the pathway as functional Compares 2 datasets	Not longer available Not yet available for microbial eukaryotes
iVikodak	Web-based	16S rRNA	KEGG, Pfam, COG, TIGRfam	Functional inference	Microbial co-inhabitance patterns	RDP, Greengenes, SILVA	User-friendly for non-expert bioinformaticians Integrated tools for statistical comparisons Graphical visualizations	Available online only Not yet available for microbial eukaryotes
FUNGuild	Python/Web-based	ITS	Guild type	Trait assignment	Not applicable	Based on UNITE taxonomy (ITS)	Trait quality for taxon assignment	No regular update 18S rRNA taxonomy with related database not included. However, the database is open-access, and a homemade wrapper can be used for 18S metabarcoding output
FAPROTAX	Python; flat file	16S rRNA	Ecological functions (e.g., nitrification, denitrification, or fermentation)	Trait assignment, Database	If all type strains of a species at the genus level share the function, FAPROTAX assumes that all uncultured organisms of this genus possess the putative function	SILVA (128, 132)	Based on the literature of cultured taxa Availability of all literature to create the database Functions easily added to the tool	Implicit assumption (see Methods column) could be false with the increase of newly cultured organisms Does not infer upper rank when taxonomic resolution is poor
BacDive	Python and R API, R package		Morphology, physiology (API®-tests), molecular data, and cultivation conditions	Database	Not applicable	NCBI taxonomy	Provides links to ENA, GenBank, SILVA, BRENDA, GBIF, ChEBI, Straininfo website data A match with 16S rRNA sequences is available from SILVA	Does not provide a tool for metabarcoding output
BugBase	R/Python	16S rRNA	KEGG	Functional inference	PICRUSt, custom trait assignment	Greengenes	Biogically interpretable traits (Gram staining, oxygen tolerance, biofilm formation, pathogenicity, mobile element content, and oxidative stress tolerance)	No peer-reviewed publication (bioRxiv preprint)
IJSEM	Flat file with R script for curation		IJSEM	Database	Not applicable	Not applicable	16S rRNA accession numbers available	Does not provide a tool for metabarcoding output
ProTraits	Web-based; flat files		Wikipedia, MicrobeWiki, HAMAP proteomes, PubMed abstracts and publications, Bacmap, Genoscope, JGI, KEGG, NCBI, Karyn's Genomes	Database	Not applicable	Not applicable	Phenotypic inference large ressource (∼545,000 phenotypes scanning 424 traits across 3,046 species) NCBI taxonomy available	Does not provide a tool for metabarcoding output
BURRITO	Web-based	16S rRNA	KO	Functional inference	PICRUSt	Greengenes	Explores simultaneous and integrative studies of taxonomic and functional profiles	Based on PICRUSt v1
MACADAM	Python/web implementation	16S rRNA	MetaCyc, MicroCyc, FAPROTAX, IJSEM	Functional inference, Trait assignment	Custom methods (provides functional information about upper-rank taxa when organism name is not found)	NCBI taxonomy	Pathway score and pathway frequency score are provided, allowing knowledge of number of enzymes present in the pathway	Not yet available for microbial eukaryotes
FunFun	R package; flat file		Ecological traits	Trait assignment	Not applicable	Based on UNITE taxonomy (ITS)	Uses R (multiplatform) Complementary to FUNGuild	
FungalTraits	Flat files		Guild type, body type, habitat	Trait assignment	Not applicable	Based on UNITE taxonomy (ITS)	Expert work to propose traits at the genus level Merges the FUNGuild and FunFun tools An excel file with vlookup function is available to assign guilds or trait data	Does not provide a tool for metabarcoding output
DEEMY	Web-based		Morphology, anatomy, potential for chemical reactions, or even ecology traits	Database	Not applicable	Not applicable	Link to tree species associated Includes images	Specialized in ectomycorrhizas only
Bacteria-archaea-traits	R package; flat file	16S rRNA	Traits, phenotypic traits, quantitative genomic traits	Database	Not applicable	NCBI taxonomy, GTDB taxonomy	Groups the major bacterial and archaeal databases into 1 database Traits and species data condensed R workflow available to retrieve condensed trait and species data	
OntoBiotope	Web-based		Habitats and phenotypes	Database	ToMap (Text to ontology mapping)	NCBI taxonomy	Term relevance is evaluated by the semantic search engine PubMedBiotope Maintained by ∼30 microbiology experts	Dedicated to the food domain
@Minter	Python		Microbial interactions	Machine learning	Support-vector machine (SVM)-based classifier	No specific taxonomy, just species level	Original approach to get information on microbial interactions rapidly	Species name required

### Functional inference

#### Definition

Functional inference consists of predicting the functional potential of a microbial community from metabarcoding data. The functional potential of a taxon or of a microbial community represents the metabolic capacities based on the presence/absence of genes involved in these pathways. Functional inference methods are based on the assumption that phylogenetic information from marker gene sequences correlates well enough with the genomic content to produce accurate predictions when associated reference genomes are available. In other words, it assumes a significant relationship between (i) the phylogenetic distance between taxonomic markers and (ii) the conservation of the genetic content, referring to vertical gene descent during the evolution of microbial genomes. This is made possible through the relationship between the phylogenetic relatedness of organisms and their gene content [64, 65] (Fig. 5B).

It should be emphasized that the presence of 1 or more genes involved in a function remains “potential” and may not be expressed under environmental conditions. From this point of view, functional inference results may be similar to shotgun metagenomics data; which is often observed in the literature, especially when focusing on a family of genes or a specific biogeochemical cycle [66]. Also, the fact that inferred metagenomes are based only on the reference genomes available in these tools (archaea, bacteria, fungi) means that the lateral gene transfer and gene loss cannot be studied, unlike shotgun metagenomics.

#### Available tools

##### PICRUSt.

Phylogenetic Investigation of Communities by Reconstruction of Unobserved States (PICRUSt) v1 [60] is the first tool to have been developed to predict potential functional genes from 16S rRNA metabarcoding and has been the most popular one since it was launched in 2013 (Fig. 5B). PICRUSt v1 needs 3 things: (i) a reference OTU, (ii) a reference genome, and (iii) a reference phylogenetic tree. As regards the reference OTU, the file (in BIOM or tabulated format) is expected to contain a standard OTU abundance table with sequences picked only against the Greengenes taxonomic reference (18 May 2012 or v13.5/v13.8). This tool based on a modified method of ancestral state reconstruction (ASR) deduces functional information for taxa without a match in the reference genomes. The reference genomes are functional proxies that provide a weighting of the functional profiles for the phylogenetically close taxa within a reference phylogenetic tree. The PICRUSt method is divided into 3 main steps that are necessary to obtain relevant information on functional profiles: (i) genome prediction, (ii) metagenome prediction, and (iii) analysis of predictions.

The genome prediction step consists of preparing the trees and checking the quality of the input datasets; then comes the reconstruction of ancestral states in the reference tree (ASR; 4 methodologies are available). Using the output files, the software program predicts traits for leaves of the phylogenetic tree lacking sequenced genomes.

During the metagenome prediction step, normalization of the abundance of each OTU is carried out on the basis of rRNA gene copy numbers (GCNs) to predict the functional category abundances of the metagenome. The user obtains an abundance table for each functional category per sample. The correcting step of the rRNA GCNs allows normalizing to correct the biases towards microorganisms with greater GCNs and improve the estimation of microbial diversity [67]. This step is recommended when the OTUs are phylogenetically closely linked to the genomes [68]. To assess the robustness of the predictions, i.e., to obtain the representativeness of the database towards a community of interest, a nearest sequenced taxon index (NSTI) is generated for each sample. It is calculated using the average of the branches that separate the sequences of interest (OTUs, amplicon sequence variants [ASVs]) in a sample from the reference microbial genome, with a weighting by their relative abundance in the sample. This confidence score is one of the major strengths of this tool. Regarding functional categories, information can be obtained at different levels (genes or metabolic pathways) with more or less detailed descriptions (EC numbers, KEGG pathway [40], cluster of orthologous groups [COG]). Information about all functional categories can also be obtained for each OTU. The last step consists of analyzing the predicted data. This step is essential for interpreting the large number of results generated from a robust statistical analysis.

The major strength of PICRUSt v1 lies in its evolutionary models that infer functions for the complete bacterial community. The portability of this tool with the support of a broad stakeholder community including a forum (Google group) and blogs are advantages that make it a central tool for functional predictions (Table 2). Despite all its benefits, PICRUSt v1 has drawbacks such as focusing only on the 16S rDNA marker and using only Greengenes taxonomy (Table 2). Several specialized tools have emerged to integrate PICRUSt as a sublayer to carry out diagnoses in the medical field [69] or directly in a pipeline [70]. PICRUSt v2 fills the gaps of the first version, with an improvement that allows inference directly based on the sequences and no longer through taxonomy. Another improvement concerns the addition of bacterial but also fungal reference genomes, thus making it possible to infer from 18S rDNA and ITS amplicons [71].

PAPRICA. Pathway Prediction by Phylogenetic Placement (PAPRICA) [72] infers the metabolic potential of prokaryotic and eukaryotic communities from metabarcoding data based on rRNA gene amplicons. It was the first tool that allowed for the functional prediction of 16S and 18S rRNA amplicons. It comes in the form of a pipeline taking the OTU reads as inputs to place them in an rRNA reference tree built from complete genomes. To build this tree, a consensus genome is found for each node in the tree, which then makes it possible to predict metabolic pathways for the sequences of interest without a match in the complete reference genomes. The abundance of metabolic pathways is weighted by rRNA GCNs from known genomes. A strength of this tool is that it also provides an indicator of genomic stability depicting the robustness of the results. However, PAPRICA, like all the tools using a reference phylogenetic tree and sequence placement methods, is dependent on the quality of rRNA resolution, and this represents a drawback when some clades may be affected (Table 2).

Tax4Fun. Tax4Fun [62] is an R [73] package published in 2015 for predicting functional profiles from targeted metagenomic 16S rRNA data. However, the algorithm and statistical efficiency based on a metabolic mixture model in terms of a mixture of pathways was developed in 2013. This R-based architecture is inherently a cross-platform tool, and it may be more accessible for a large number of users with low experience in bioinformatics. This tool uses pre-calculated functional profiles like PICRUSt v1 and taxonomic data formatted from the SILVA database. One of the differences with PICRUSt is the rRNA sequence placement in the reference genomes, which is achieved by a BLAST search (instead of a tree placement approach as for PICRUSt). It is a convenient tool because it provides a confidence score (FTU and FSU) to determine the fraction of OTUs that was not mapped to KEGG organisms or the number of sequences without KEGG Orthology (KO) hits (Table 2). Like PICRUSt v1, it cannot be used for fungal diversity predictions.

Piphillin. Piphillin [74] differs from the PICRUSt or PAPRICA approaches because it does not use a phylogenetic tree or database (16S) but directly maps the OTU sequences on the rRNA of the reference genomes using a nearest-neighbor algorithm. This specificity could avoid faulty sequence placements in the reference phylogenetic tree. It is used online only, which represents both a strength and a weakness: it benefits from computing power (a strength), whose strength depends on the hosting server (e.g., quota management, cluster configuration) (a weakness). A Piphillin sublayer also exists to complete the analysis of the results [75].

**Figure 8:**
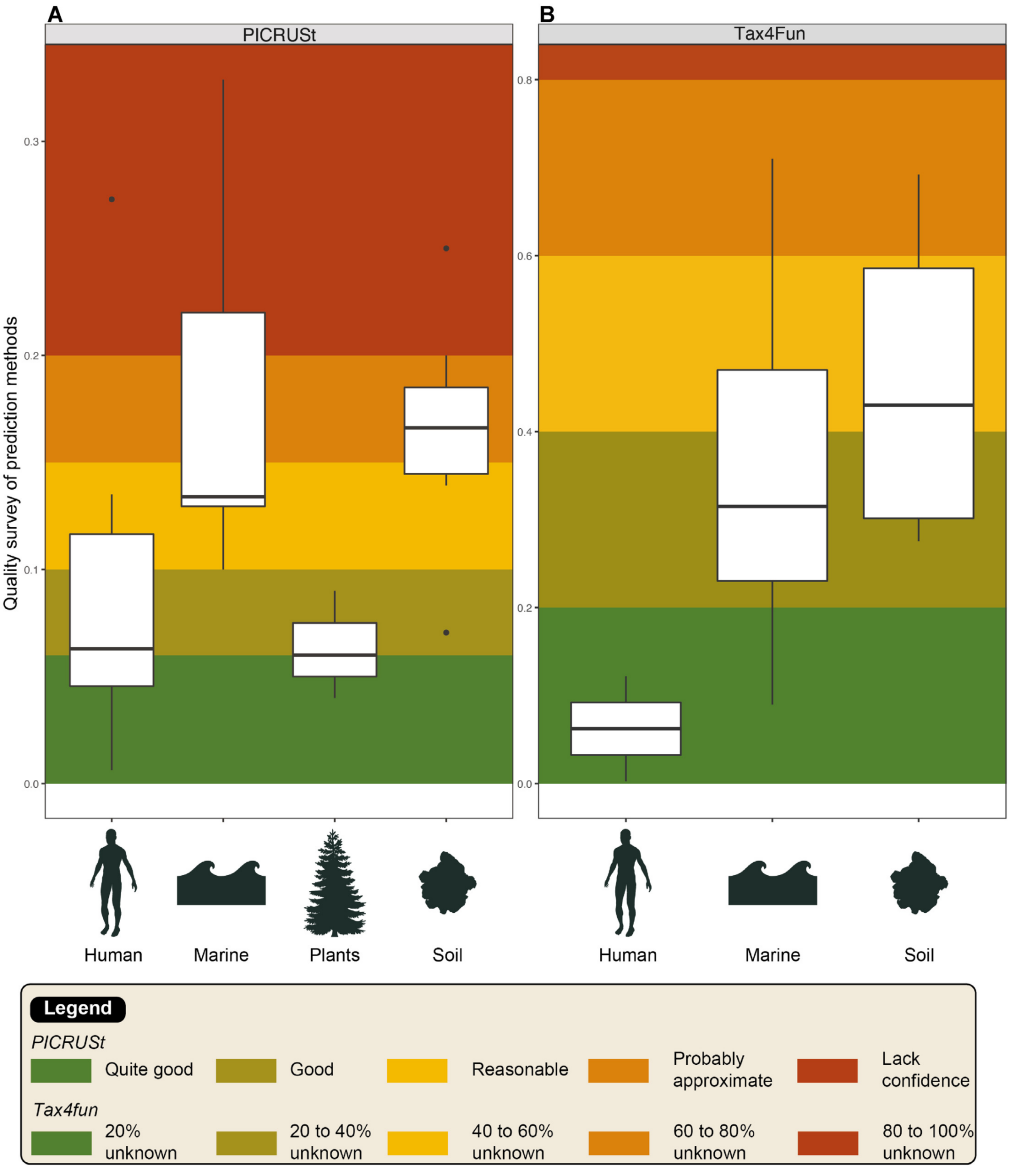
Overview of the quality of functional prediction based on a subsampling of articles for PICRUSt (A) and Tax4Fun (B) across various ecosystems. For PICRUSt, colors were assigned according NSTI results: <0.06, quite good; 0.06–0.10, good; 0.10–0.15, reasonable but probably approximate; and >0.20, probably unreliable. For Tax4Fun, we split the fraction of OTUs that could not be mapped to KEGG organisms in 5 harmonious groups. References are listed in Additional File 1. The distribution of data are displaying by boxplots and are standardized way of based on a five number summary (minimum, first quartile, median, third quartile, and maximum) and the outliers (shown as black circles).

The quality of prediction represents a prerequisite for the application of the above-presented tools to study indigenous microbial communities. It may depend on the tool but also on the type of targeted ecosystem. To test the quality of functional prediction according to the tool and the studied ecosystem, we compiled the NSTI scores for PICRUSt v1 and the FTUs for Tax4Fun from a subsampling of articles that covered a range of ecosystems—human, marine, plant, and soil (Fig. 8). Whatever the tool, the best predictions were obtained for the human microbiotas, and the most approximate ones, for the soil samples. The variability of quality scores across the different soil studies seemed to be lower with PICRUSt than with Tax4Fun. Nevertheless, some soil studies using Tax4fun indicate a high-quality survey, with only ∼30% of OTUs unmapped to a reference. This likely reflects the discrepancy between human reference genome availability and soil microbiota genome availability. In addition, microbial diversity is much more complex in soils than in the human microbiotas. In this case, it is essential that the quality scores from functional inference tools should be taken into account because it is a key to a robust interpretation of the results. Unfortunately, we found few studies indicating these quality scores.

### Ecological trait assignment

#### Definition

Ecological trait assignment differs from functional inference because it consists of obtaining information on the life strategy, phenotypic, and quantitative genomic traits (e.g., trophic modes, growth strategy) of a taxon from its nomenclature, whatever its taxonomic rank. If the taxon is not present in the database, it will not be possible to know its traits (Fig. 5C). This approach is faster than functional inference for retrieving an item of functional information, but tools dedicated to metabarcoding outputs are lacking, and only a few ecological traits are available (Table 2). The main interest is to get functional information with a possibly not so fine granularity as functional inference does, but obviously more accurate. Ecological traits are indeed often based on results with biochemical experimentations from curated databases or scientific publications. Practically speaking, only the guild will be recovered and for example the fungal sequences identified as belonging to the *Serpula* genus will be assigned to a wood saprotroph when an ecological trait tool is used; with an inference tool, the abundance of various genes related to polysaccharide degradation will be attributed to all fungal sequences.

#### Tools

##### FUNGuild.

FUNGuild [61] is the pioneer and one of the few tools that assigns ecological traits to fungi based on their taxonomy (Table 2). These assignments rely on metabarcoding data. They require providing a contingency table (OTUs or sequence counts *per* sample) and the link between each OTU and its taxonomy. To carry out the assignment, FUNGuild uses its own curated database, and searches it for the taxon. This database contains several taxonomic levels (e.g., phylum, genus, species). However, the taxonomic name at the genus or species level is necessary to assign traits to the taxa of interest. Trait information is available in 66% of the cases at the genus level, and only in 34% of the cases at the species level [61]. The user obtains a summary table of the different possible ecological traits for each taxon with a robustness indicator and a confidence range (“possible,” “probable,” and “highly probable”).

The strength of this database is that the provided data are based on the literature (primary research), or on reference websites or their own collective research experience if the datum is missing. The authors recommend the use of the UNITE database for taxonomic assignment and therefore the use of the ITS marker, but it can be easily transposed to data based on the 18S rRNA marker. It just requires creating a wrapper to make a link between the taxonomy of the data and FUNGuild to retrieve the traits of interest.

A new database called Fun^Fun^ [76] is now available. It encompasses 80 fungal ecological traits. In reality, this database is a FUNGuild database overlay with information on genetic, enzymatic, morphological, stoichiometric, life history, and physiological aspects. In addition, the authors mention that Fun^Fun^ will be updated in terms of taxonomy and associated guilds, which is not necessarily the case with FUNGuild. However, although this database is promising, a lot of information is missing because it integrates literature data for the first time ever, and its improvement relies on the progress of research, as well as the contribution of scientists. This caused an impulse leading to a community of scientists proposing a new database: FungalTraits [77] links information from FUNGuild and Fun^Fun^. It is very complete, and offers different levels of life styles. Please note that this database includes species from the fungal kingdom but also fungus-like stramenopiles (e.g., the Oomycota phylum). This may be especially useful because various species are identified as major plant pathogens within Oomycota. For example, the genus *Phytophthora* gathers several crop pathogens that cause important losses and can represent a risk to global food security [78].

To conclude, the minor drawbacks of FUNGuild, with rare updates or a tool oriented to ITS sequences, have been offset by the new Fun^Fun^ and FungalTraits databases.

To complete the tools concerning fungal communities, DEEMY [79] is an information system only available online and specialized in ectomycorrhizas [80]. This website references 554 species associated with their respective symbiotic organisms, including 104 genera. To characterize each species, a summary sheet provides taxonomic nomenclature and bibliographical references and photographs, as well as information on morphology, anatomy, potential chemical reactions, or even ecology traits.

FAPROTAX. Functional Annotation of Prokaryotic Taxa (FAPROTAX) [63] is used to assign metabolic functions, ecological traits, or large functional groups relevant to prokaryotes (Table 2). This database was built manually from the scientific literature of the *International Journal of Systematic and Evolutionary Microbiology* (IJSEM) and Bergey's *Manual of Systematic Bacteriology*. It contains ∼4,700 unique prokaryotic taxonomies (mostly at the species level) and 90 functional groups. FAPROTAX is based on the implicit assignment of a trait/function to a taxon (whether cultivated or not) if all the cultivated members display this trait/function. Its main limitation is that it is focused on marine prokaryotic organisms, so communities from other biomes can be missing. Another point to be considered is that if the taxa of interest do not have a species name, the tool cannot draw inferences at the upper levels (e.g., genus) to assign an ecological trait.

IJSEM phenotypic database. IJSEM [81] compiles phenotypic and environmental tolerance data about >5,000 bacterial strains. It is an official and unique reference for publishing and validating new strains. These strains cover ∼23 phyla from various habitats (mainly soils). The database appears as a TSV file [82], and available information can be grouped into 5 categories: ancillary data (e.g., article's DOI; taxonomic nomenclature), morphology/phenotype (e.g., Gram stain status; motility), metabolism (e.g., BIOLOG information), environmental preferences (e.g., habitat of isolation; oxygen requirement), and sequence data (e.g., 16S rRNA accession No.).

BacDive. BacDive [83] is one of the largest metadatabases [84] referencing information on bacterial and archaeal diversity (Table 2). The tool links taxonomy and phenotypic information directly, but the database can only be browsed on a website or data can be downloaded from it. However, it provides a complete API to achieve scripts and retrieve the desired information. In the first months of 2020, it offered data on 81,827 bacterial and archaeal strains, including 14,091 type strains, and thereby covered ∼90% of the described species according to their website. This database is interesting because it provides different levels of robust information on taxonomy, morphology, physiology (API®-tests), molecular data, and cultivation conditions. As for physiological data, it provides—for example—the main substrates used for culturing a species and the enzymes present (a link with the EC classification number is available). These data have been more broadly incorporated into a tool (bacteria-archaea-traits) that encompasses numerous traits of bacteria and archaea from 26 sources [51].

To complete this list, a few specialized databases target only 1 or a few traits. For example, Engqvist [85] recently grouped the growth temperatures of 21,498 non-redundant organisms across the whole tree of life. This study showed a strong correlation between the growth temperature of organisms and enzymatic optima, with temperature-dependent increases or decreases of enzymatic functions. This information can be very interesting and complementary to the interpretation of functional inference results, and can be linked—for example—to environmental conditions.

## Application of These New Approaches to the Functions of the Soil Microbial Ecosystem

### Functional inference

**Figure 9:**
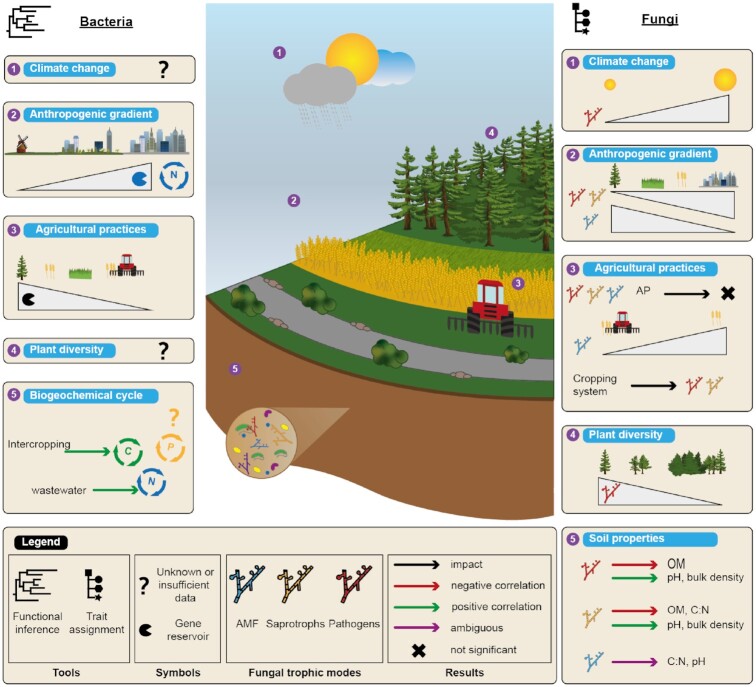
Summary diagram of the most relevant microbial soil functions results based on functional inference and ecological trait assignment. The figure is made up of 2 parts: studies on bacterial communities based on functional inference on the left and studies on fungal communities based on ecological trait assignment on the right. For all studies (climate change, anthropogenic gradient, agricultural practices, plant diversity, or the biogeochemical cycle), if an effect or a correlation was found on the gene reservoir or on microbial communities with a particular ecological trait, a colored arrow indicates the effect and a cross indicates no significant effect. A triangle indicates either a decrease or an increase of the gene reservoir or microbial communities with a particular trait. References are listed in Additional File 1.

In recent years, meta-omics approaches have been increasingly included in soil monitoring, whether in fundamental research programs or in more operational projects [86]. Most studies (∼60% on the basis of keywords in the titles or abstracts of the publications, see Fig. 7B) have focused on PICRUSt to generate functional predictions from taxonomic data of the soil microbiota. We summarized the most valuable outcomes about soils by grouping them into categories: anthropogenic gradient, agricultural practices, and biogeochemical cycle or soil properties (Fig. 9). For example, a study showed that plant-bacteria interactions in the rhizosphere were mainly related to beneficial cooperation [87] involving the release of root exudates by the plants on the one hand, and hormone production or the ability to break down toxic chemicals by bacteria on the other hand. Another study investigated the stoichiometric regulation of soil carbon cycling by comparing functional predictions by metabarcoding (via PICRUSt) and shotgun sequencing on a wide C:N:P soil gradient in a rice field [66]. A strong correlation was evidenced between the functional predictions from metabarcoding and metagenomics as regards the abundance of some metabolic families involved in the C, N, and P cycles. Still using PICRUSt, another study examined the effects of intercropping by predicting the soil microbial functional profiles. It evidenced that an intercropping system increased the functional potential in terms of carbon fixation pathways and the citrate cycle [88]. Finally, a study focused on the impact of long-term land-use practices (forest, grassland, crops) on soil bacterial communities [89] showed that forest soils harbored the largest reservoir of genes, followed by no-till soils and then grasslands. The plowed soils presented the lowest functional richness.

Based on Tax4Fun predictions, a study investigated the effect of different irrigation practices with various water qualities (freshwater, treated or untreated wastewater) along with the different land use systems in drylands [90]. The authors compared the potential functional and taxonomic profiles of bacteria. Irrigation with wastewater had an effect on bacterial responses by shaping communities and functional profiles. By bringing more nitrogen, wastewater favored the response of certain genera, in particular *Nitrosospira*, and increased the relative abundance of the genes involved in nitrification and denitrification.

Among all the functional inference tools available today, 2 of them stand out, i.e., PICRUSt and Tax4Fun. A benchmark study of these tools found no major differences in terms of performance, especially for soil samples [91]. Another benchmark study indicated that these 2 tools provided similar functional profiles but could be complementary for certain gene families found only in one or the other [92]. Moreover, the characterization of the fungal functional potential by PICRUSt2 is too recent for us to have any insights into its robustness concerning soil communities. Compared to trait assignment, the links between diversity and functions still remain tenuous concerning certain biogeochemical cycles or the impact of climate change and plant diversity (Fig. 9).

### Ecological trait assignment

The complexity of microbial traits is variable, with simple traits like organic phosphate utilization and more complex ones like methanogenesis [24, 93]. The conservation of prokaryotic traits or core genes varies according to phylogenetic depth [64]. For example, the complex methanogenesis trait seems to be very conserved at the order and family levels, which contrasts with the resistance to specific bacteriophages, which seems to vary at the species level owing to particular point mutations [24]. Below are a few examples of the possible benefits of ecological traits to the analysis of the diversity of soil microbial communities (Fig. 9).

Regarding the assignment of fungal traits, FUNGuild is currently and by far the most implemented tool, if not the only tool implemented by ecologists wishing to supplement their diversity analyses with data on the ecological traits of fungal communities, and mainly in studies on soil fungal communities [94–97]. A study on fungal communities in subtropical forest soils highlighted a negative relationship between the abundance of pathogenic fungi and the phylogenetic diversity of plant communities [98]. Another study showed a positive correlation between soil fungal community dissimilarities (plant pathogens, saprotrophs, and ectomycorrhizas) and plant phylogenetic distances in forest soils [99]. Tropical land uses also affect the functional guild. A massive shift of fungal trophic modes has been shown—notably a decrease in mycorrhizal fungi and an increase in saprophytic and pathogenic fungi—along with increased anthropization levels [100]. Interestingly, several large-scale (national or global) studies have characterized the distribution of trophic types while identifying the environmental parameters that influence them [94, 101–103]. The distribution of these trophic modes seems to vary greatly depending on temperature and precipitation [103]. This supports a recent global study focused on the distribution of pathogens and indicating higher abundance in warm regions [102]. A recent study compared the trophic modes (synonym: life strategies) assigned to the ITS and 18S rDNA molecular markers by FUNGuild [94]. This study indicated that the saprotroph and pathotroph richness levels were directly and negatively correlated with the organic matter content and elevation, and positively correlated with the pH and bulk density. For symbiotroph richness, the relationship differed depending on the molecular marker used: it was positively correlated with the C:N ratio when ITS sequences were used but negatively correlated when 18S rDNA sequences were used. Similarly, the pH was positively correlated on the basis of 18S rDNA data but negatively correlated on the basis of ITS data [94]. These differences may come from the fact that the 2 molecular markers do not cover the same taxonomic range. Therefore, the choice of molecular markers and primers is essential because it affects the global picture obtained by possibly enhancing or decreasing the representation of particular functional groups in the community. For example, arbuscular mycorrhizal fungi are better represented, in particular the Glomeromycota group, when the 18S rDNA marker is used [104, 105]. A study at a smaller scale also showed that saprotroph richness was directly driven by the soil physico-chemical parameters and confirmed the aforementioned results. The authors showed a positive correlation with the pH but a negative one with the C:N ratio [106]. All these studies used the FUNGuild tool dedicated to characterizing fungal community traits.

Regarding the assignment of bacterial traits, various databases exist but few tools have been developed to assign ecological traits from metabarcoding datasets. Only FAPROTAX stands out as a powerful tool for analyzing the functional potential of soil communities [107], although it is dedicated to marine organisms.

## Technical and Conceptual Limitations and Biases

The metabarcoding approaches have significant advantages for characterizing indigenous prokaryotic and eukaryotic microbial communities. Standard protocols now exist, from sample preparation to bioinformatic and statistical analyses, and scientists have acquired an important feedback on biases, costs, and efficiency [108–110].

A fundamental limitation of functional inference tools, represented by gene gain and loss, is mainly due to horizontal gene transfer but also gene duplication, gene loss, and *de novo* gene birth [111–114], which is addressed in the literature and taken into account to some extent in these tools. However, horizontal gene transfer remains difficult to consider accurately for functional prediction, and its influence on microbial communities is hard to estimate. Moreover, the horizontal gene transfer rate varies substantially within the tree of life and according to gene families/pathways [24, 93, 111]. This process is mainly described in prokaryotes but is also found to a lesser extent in eukaryotes, in particular fungi [115]. Microorganisms can gain a function through plasmid transfer, but no information was found in the literature about functional prediction [60]. However, plasmids are extrachromosomal DNA molecules that play a role in the rapid adaptation of microbial communities to environmental changes across all microbiomes [116, 117]. In particular, they are transferred between phylogenetically distant populations for them to acquire genes and beneficial traits for their adaptation (e.g., resistance to antibiotics, biocides, pollutants). This is key for all environments, especially soils, where biotic and abiotic fluctuations are tremendous [118]. The transfer of plasmids is also introduced from phages or viruses into microbial genomes [119].

**Figure 10:**
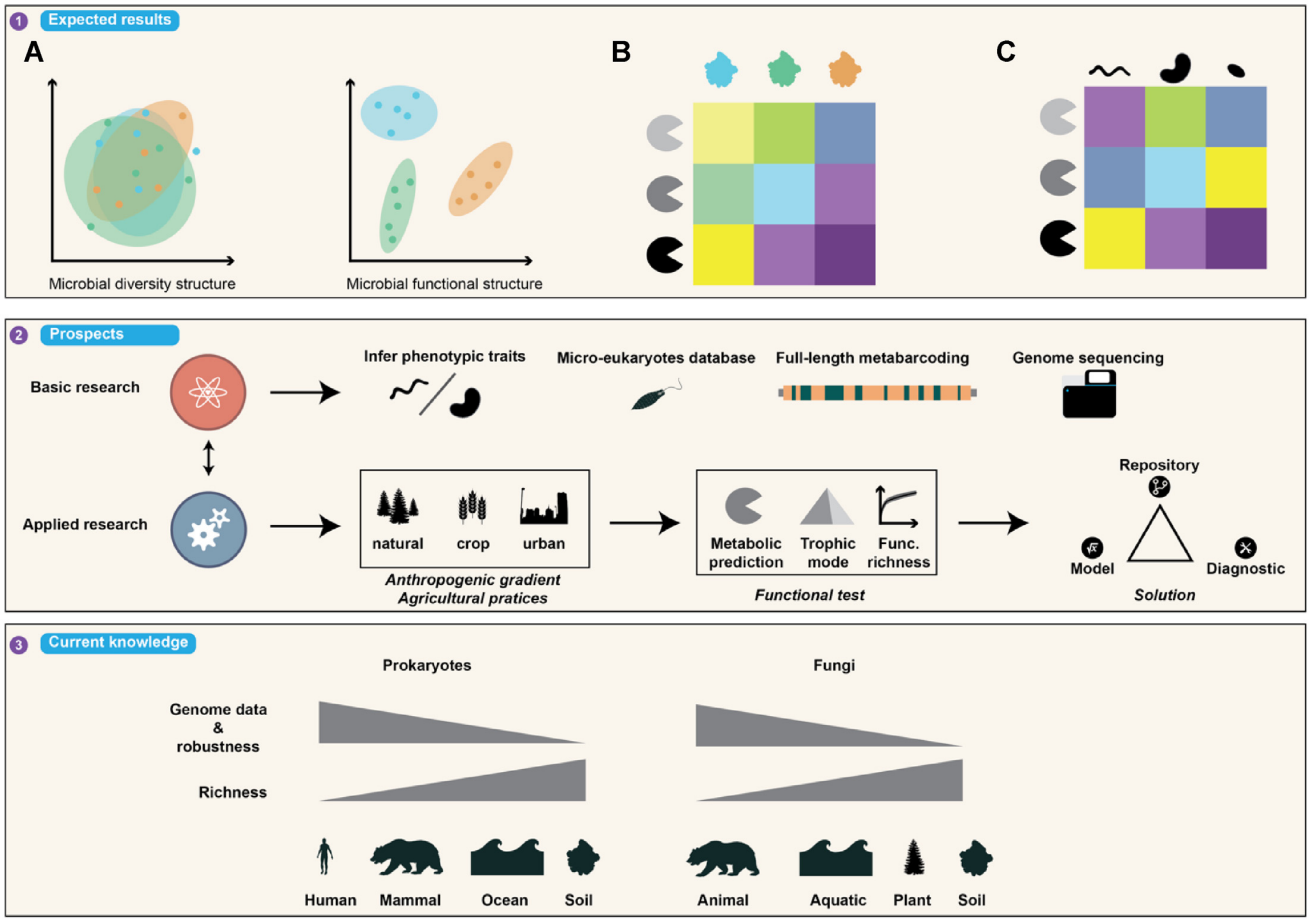
Summary diagram of the expected results (first box), the functional prediction prospects (second box), and the limits of the microbial genomic data available for different habitats (third box). The first box illustrates a comparative example of data results of community structures and functional structures through a PCA (A). This example illustrates the case when the functional community structure differentiates experimental conditions better than it differentiates the microbial community structure. Illustrative heat maps showing the relative abundance of genes per sample (B) or per OTU (C).

From a technical point of view, most of the studies on microbial diversity using metabarcoding approaches are based on the sequencing of 1 or more hypervariable regions and remain limited by the size of the amplicon to be sequenced. The most commonly used Illumina sequencing platforms (MiSeq, HiSeq, and NovaSeq) can provide maximum readings of 600 bp (∼550 bp after adapter/tag/primer trimming). Several studies have questioned the most suitable regions for obtaining the best taxonomic resolution [120, 121]; the use of full-length rRNA (∼1,800 bp) seems to be the most appropriate solution [122]. It would significantly enhance phylogenetic resolution for prokaryotic and eukaryotic microorganisms [123] (Fig. 10, second box). Short reads do not allow good enough resolution in taxonomic assignment either (i.e., not down to the species level), although this point is crucial for placing sequences/taxa in the phylogenetic tree to achieve functional inference. With third-generation HTS platforms (e.g., PacBio, Oxford Nanopore), full-length molecular markers can be sequenced, e.g., 16S/18S rRNA genes or the full ITS1 and ITS2 sequences [124, 125]. This will considerably improve taxonomic assignment and make it possible to assign sequences at the species or even the strain level in certain cases [125]. This way, functional inference and ecological trait assignment will be improved. However, if the objective is to obtain the best taxonomic resolution possible, the study of ecological traits at high taxonomic ranks (e.g., the phylum) remains very promising, especially for highly conserved traits [126]. For example, the carbon mineralization rate was positively (e.g., Bacteroidetes) or negatively (e.g., Acidobacteria) correlated with their relative abundance [127].

A good practice complementary to the use of full-length amplicon sequencing would be the use of ASVs (also called ZOTUs) to increase the rate of inference with a better sequence placement on the reference tree [71, 128]. Indeed, for those using an OTU clustering approach with a similarity threshold, 1 solution would be to use all the sequences within the OTUs instead of 1 representative sequence for each OTU seed, which could be less accurate. However, this would also increase the analysis time.

## Importance of Taxonomy and Genome References: From Accuracy to Resolution

Many tools use taxonomic data to obtain information about microbial functions through a metabarcoding approach. Therefore, it is important to check the bioinformatic strategy used to analyze the amplicon sequences, from the filtering steps to OTU clustering or not (see ASV), including taxonomic assignment.

The use of tools on ecological traits is highly dependent on taxonomic resolution. For example, when using FUNGuild, special attention must also be paid to the fact that a sequence assigned at the genus level may be associated with several trophic types, and that plant-pathogenic fungi are highly host-specific and may be non-pathogenic in the context of the study. For the sequences (or OTUs) without any taxonomic assignment, functions cannot be obtained using tools on ecological traits (Fig. 10, second box). To improve this point, especially for fungal communities, inferences may be drawn on the basis of phylogeny, as done for bacteria, archaea, or macroorganisms [129–133]. One of the avenues to be explored is the use of ASR tools such as PICANTE [34] or CASTOR [36], which infer traits for taxa devoid of ecological data from a phylogenetic tree.

Functional inference tools depend on the reference genomes to establish predictions, so the accuracy of the results can vary among samples. Samples with well-described host-associated communities such as the human microbiome have many reference genomes available and allow good predictive accuracy (Figs 8 and 10 third box). Contrastingly, in more complex and highly biodiverse environments like soils [38], the genomes representing the total taxonomic diversity are much more difficult to obtain. The proportion of cultivable terrestrial strains remains very low (∼25%) compared to the human microbiotas (80%) [134]. Thus, the results estimated for the communities from complex biomes are approximate and debatable.

To improve functional prediction results, it is advisable to provide genomes specific to the habitat of interest [135]. Considerable efforts have to be made to increase the number of habitat-specific reference genomes (animal/human, water, plant, soil), with special attention to the most complex and unknown environments [136]. Tools to routinely update the databases will also need to be developed [137]. This is an ongoing dynamic at the international scale. For example, the annotation of reference genomes in databases is not yet representative of soil microbial diversity [138]. To fill this gap, an effort has been made by creating the Refsoil database [138] (which does not seem to be maintained [139]) or a Refsoil + plasmid database [117].

## Discussion and Future Prospects

The possible retrieval of a putative functional potential or ecological traits directly from taxonomic markers and metabarcoding approaches opens new perspectives for our understanding of microbial communities, both from a fundamental and/or an operational point of view (e.g., functional redundancies, diagnostic tool) [69, 140]. This information can be used to (i) understand the main functions potentially expressed in a given environment and identify the possible drivers, (ii) examine the distribution of functions among taxonomic groups, or (iii) supplement the classic diversity metrics used to evaluate the ecological state of environmental matrices (Fig. 10, first box). Beyond providing an overview of the putative functions of an ecosystem, prediction tools could also provide more detailed information than taxonomic markers do for users to significantly distinguish sample groups from each other in certain habitats [122] (Fig. 10A, first box).

A new generation of tools solves the main limitations of the previous generation tools by including improvements in terms of taxonomic marker targeting, methodology, and flexibility.

## Future Prospects with Second-Generation Tools

Second-generation tools are currently emerging, e.g., PICRUSt2 [71], Tax4Fun2 [135], or iVikodak [141] (Fig. 6). Indeed, Langille's team of developers bridged the gap for the scientific community working on fungal ecology. PICRUSt2 now includes 18S rDNA and ITS amplicons from the fungal kingdom. Another great improvement is flexibility: the sequence can be used directly, instead of taxonomy based on Greengenes nomenclature. Users are no longer dependent on taxonomy to infer functions; this is a great comfort and provides better robustness of the analyses. However, users should be wary of the results because the number of sequenced fungal genomes currently integrated in the tool is much lower than the number of bacterial genomes. It is recommended to check the quality score (e.g., NSTI) for the robustness of the results and interpretation. However, this limitation can be lifted. For example, the 1000 Fungal Genomes Project [142] is aimed at high-quality sequencing and annotation of fungal genomes so as to build a reference dataset to be used for meta-omics data analysis.

Another downside of these tools is the absence of data support for micro-eukaryotic communities, which are essential to the soil ecosystem. Protists are abundant and diverse, with a large range of functional diversity, and are highly involved in soil food webs and functioning [143, 144]. It would be particularly useful to develop tools dedicated to protists from data on ecological traits available in the literature [145].

## Challenges: From Fundamental Research to Diagnosis

Switching from fundamental research to practical applications would be interesting because although operational microbial diversity bioindicators are increasingly emerging, there is a huge gap in the functional information of microbial communities. Even if the number of species can be an indicator of the impact of biotic and abiotic factors [146, 147], the need to characterize the associated functions at the ecosystem level has become obvious to obtain a complete diagnosis with functional information on the soil microbial quality [148, 149].

As regards human health, identifying taxonomic and functional changes to estimate the contributions of taxa associated with a disease is an emerging topic [150], as, e.g., in research into gene markers involved in colorectal or oral cancers [151, 152].

Some interesting examples exist in the biomonitoring and bioassessment of water quality [153, 154], but examples for the soil microbial quality are still scarce. The huge complexity and diversity of the soil microbial community probably still limits such applications to the soil ecosystem, along with a lack of genome references. However, initiatives at the global level are in progress to access soil biodiversity using taxonomic, functional, and environmental data [147, 155]. We can also note that a real dynamic seems to be developing at the international scale to collect, standardize, and disseminate traits through the tree of life via an open science tool called the Open Traits Network (OTN) [92].

To our knowledge, providing robust and operational indicators based on putative functions derived from metabarcoding data is impossible today. The main challenges are to (i) aggregate and summarize the mass of data currently generated, (ii) test the predictions on datasets and compare them with “real” functional measurements, (iii) validate these indicators on datasets under diverse experimental conditions (e.g., land use gradient, agricultural practices) at the local and global scales, and (iv) develop representative repositories to ensure the validity of the diagnosis made from these new tools.

Regarding aggregation and data reduction (item i), a track would be to use a constrained non-negative matrix factorization approach [156], an alternative to the concept of community-aggregated traits [157]. This method has already been used to aggregate functional traits from metagenomes [156]. The authors demonstrated that significant data reduction made it possible to propose simple models to describe a set of complex functions at the scale of an ecosystem (here the potential for fiber degradation in the human intestinal microbiota) while preserving biological data quality [156]. Concerning item ii, it will be interesting, for example, to confront functional predictions with volatile organic compound (VOCs) emissions or microbial respiration rates from soil measurements. Indeed, the very diverse microbial VOCs are secondary metabolites playing various roles, in particular making it possible to carry out more or less long-distance interactions and communication (e.g., growth, motility, antibiotic resistance, expression of stress response genes) [158]. Moreover, to suggest these tools as robust indicators of the soil quality (item iii), it will be essential to use large datasets to determine the best metrics (e.g., functional richness, relative gene abundance, aggregation of traits) and the most sensitive genes or groups of genes depending on the various scientific issues. Once these limitations have been lifted, these tools will provide results of great interest to the scientific community at relatively affordable human, technological, and financial costs. However, maintaining the associated scientific expertise will be essential to support their transfer for operational applications and avoid erroneous interpretations that could potentially have disastrous consequences for soil users and soil policy makers (item iv). For example, interpreting trophic types requires strong expertise, with particular attention to the exploitation of potential pathogenicity information—a highly sensitive task. The responses of the traits vary according to the disturbances applied to the ecosystem [159], and the results must be contextualized to ensure correct interpretation.

## Conclusion

The exploration of microbial functional diversity based on taxonomic marker genes in order to improve our knowledge of microbial diversity and functions is just starting. As highlighted in this review, various solutions have emerged over a number of years and are being improved quickly thanks to technological advances. Functional inference results are already robust and representative for some ecosystems with low diversity (specific richness) and with well-characterized genomes such as the human microbiotas. Progress now needs to be made for more complex environments. The upcoming challenge, notably for environmental samples, will be to establish the link between functional predictions on reference datasets and environmental measurements. The new network SoilBON dedicated to monitoring soil biodiversity and functional ecosystems at a global scale, with particular attention to microbial diversity, is a step in this direction [3]. This ambitious framework aims to collect and analyze soil diversity on the basis of soil ecological indicators (i.e., essential biodiversity variables [160]). One purpose of this framework is to inform policy makers and stakeholders so that they can adopt measures to preserve this biodiversity.

## Data Availability

Not applicable.

## Additional File

Additional file 1

## Abbreviations

API: application programming interface; ASV: amplicon sequence variant; BLAST: Basic Local Alignment Search Tool; bp: base pairs; CAT: community-aggregated trait; CAZy: carbohydrate-active enzymes; C, N, and P: carbon, nitrogen, and phosphorus; COG: cluster of orthologous groups; DOI: digital object identifier; EC number: enzyme commission number; FTU: fraction of OTUs; GCN: gene copy number; HTS: high-throughput sequencing; IJSEM: *International Journal of Systematic and Evolutionary Microbiology*; ITS: internal transcribed spacer; INSD: International Nucleotide Sequence Database; JGI: Joint Genome Institute; KEGG: Kyoto Encyclopedia of Genes and Genomes; KO: KEGG orthology; NCBI: National Center for Biotechnology Information; NSTI: nearest sequenced taxon index; OTN: open traits network; OTU: operational taxonomic unit; qPCR: quantitative PCR; RDP: Ribosomal Database Project; rDNA: ribosomal DNA; rRNA: ribosomal RNA; SRA: Sequence Read Archive; VOC: volatile organic compound; ZOTU: zero-radius OTU.

## Competing Interests

The authors declare that they have no competing interests.

## Funding

No funding to declare.

## Authors' Contributions

C.D. and L.R. conceptualized the manuscript. C.D. drafted the manuscript with contributions from S.T., S.D., A.C., P.-A.M., and L.R. All authors read and approved the final manuscript.

## Supplementary Material

giab090_GIGA-D-21-00316_Original_Submission

giab090_GIGA-D-21-00316_Revision_1

giab090_Response_to_Reviewer_Comments_Original_Submission

giab090_Reviewer_1_Report_Original_Submission Trevor Charles -- 10/31/2021 Reviewed

giab090_Reviewer_2_Report_Original_Submission Axel KÃ¼nsnter, PhD -- 11/2/2021 Reviewed

giab090_Supplemental_File
