# Supplementary material for: Inferring microbiota functions from taxonomic genes: a review
Source: Gigascience. 2022 Jan 12;11:giab090. doi: 10.1093/gigascience/giab090 (PMC8756179; doi:10.1093/gigascience/giab090)
Supplement: giab090_GIGA-D-21-00316_Revision_1 [file giab090_giga-d-21-00316_revision_1.pdf]

GigaScience

Inferring microbiota functions from taxonomic genes: a review

--Manuscript Draft--

**Manuscript Number:**	GIGA-D-21-00316R1
**Full Title:**	Inferring microbiota functions from taxonomic genes: a review
**Article Type:**	Review
**Funding Information:**	
**Abstract:**	Deciphering microbiota functions is crucial to predict ecosystem sustainability in response to global change. High-throughput sequencing at the individual or community level has revolutionized our understanding of microbial ecology, leading to the big data era and improving our ability to link microbial diversity with microbial functions. Recent advances in bioinformatics have been key for developing functional prediction tools based on DNA metabarcoding data and using taxonomic gene information. This cheaper approach in every aspect serves as an alternative to shotgun sequencing. Although these tools are increasingly used by ecologists, an objective evaluation of their modularity, portability and robustness is lacking. Here, we reviewed one hundred scientific papers on functional inference and ecological trait assignment to rank the advantages, specificities and drawbacks of these tools, using a scientific benchmarking. To date, inference tools have been mainly devoted to bacterial functions, and ecological trait assignment tools to fungal functions. A major limitation is the lack of reference genomes – compared with the human microbiota –, especially for complex ecosystems like soils. In fine, we explore applied research prospects. These tools are very promising and already provide relevant information on ecosystem functioning, but standardized indicators and corresponding repositories are still lacking for them to be used for operational diagnosis.
**Corresponding Author:**	Lionel Ranjard, Ph.D.<br>INRA UMR 1347: Agroecologie<br>Dijon, FRANCE
**Corresponding Author Secondary Information:**	
**Corresponding Author's Institution:**	INRA UMR 1347: Agroecologie
**Corresponding Author's Secondary Institution:**	
**First Author:**	Christophe Djemiel, Ph.D.
**First Author Secondary Information:**	
**Order of Authors:**	Christophe Djemiel, Ph.D.
	Pierre-Alain Maron, Ph.D.
	Sébastien Terrat, Ph.D.
	Samuel Dequiedt
	Aurélien Cottin, Ph.D.
	Lionel Ranjard, Ph.D.
**Order of Authors Secondary Information:**	
**Response to Reviewers:**	Dear Editor,<br><br>Please find enclosed the revised version of the paper Submission ID GIGA-D-21-00316 Inferring microbiota functions from taxonomic genes: a review by Christophe Djemiel, Pierre-Alain Maron, Sébastien Terrat, Samuel Dequiedt, Aurélien Cottin, Lionel Ranjard.<br>We answered carefully to the all comments of the reviewer 1 and 2. We thank the 2 reviewers, for their helpful comments during the reviewing process. We hope that the paper is now suitable for publication in GigaScience, and we look forward to hearing

Powered by Editorial Manager® and ProduXion Manager® from Aries Systems Corporation

from you.
Kind regards
Christophe Djemiel & Lionel Ranjard

Reviewer reports:
Reviewer #1: This review manuscript provides an overview of the various options that have been developed for inferring function from taxonomic data, for Bacteria, Archaea and Fungi. It should be a good resource and introduction to the topic. One aspect that could be developed a bit more is that of specificity in functional prediction.

First of all, we want to thank the Reviewer 1 for these commentaries and he pinpoints some drawbacks to help us improving it.

The specific corrections, and modifications advised by the Reviewer 1 will be highlighted in Green in the manuscript.

Throughout, please change "consists in" to "consists of"
As requested, we did the correction, and checked in the whole manuscript (lines 205, 239, 258, 330).

Line 113: It is unclear why soil quality is mentioned here
Indeed, we chose to delete the "soil quality" term to avoid focusing our sentence only just on soil diagnosis, keeping only the global point of view in this background section. We also changed the next sentence in order to ensure consistency (“A repository” to “Repositories”).

Line 145: Change "ocean" to "marine" or "aquatic"?
As requested, we did the correction in the sentence.

Line 148: Change "rDNA" to "rRNA gene"
As requested, we did the correction in the sentence.

Line 219: The comparison here to shotgun metagenomics should consider the impact of horizontal gene transfer and gene loss.
As requested, we have added a sentence to indicate that inferred metagenomes do not allow the study of lateral gene transfer and gene loss unlike shotgun metagenomics. In addition, only archaea, bacteria, and fungi can be studied directly by these tools, unlike shotgun metagenomics which provides an overview of all microbial communities.

Similarly, Line 545 should consider gene deletion in addition to horizontal gene transfer.
As requested, we have completed our sentence to indicate that gene gain and loss was also due to gene duplication, gene loss, and de novo gene birth in addition to predominant mode HGT. We have also added bibliographic references regarding the identification of HGT from shotgun metagenomic data.

Line 552: In addition to plasmid transfer, should consider phage / viruses
As requested, we have added a sentence to complete our paragraph on the transfer of plasmids by phages or viruses.

Line 665: It could be instructive to provide more examples of practical applications
Indeed, we were in the same expectation as reviewer 1 but there are very few examples of practical applications. Moreover, this is what we underline throughout our manuscript. However, as requested, we added two examples in the human health on the search for cancer biomarkers and two examples on the biomonitoring of water quality. We have also added bibliographic references regarding these practical applications.

Line 699: It is not clear why these particular soil measurements are of interest
Yes, we fully agree with the reviewer 1. As requested, we added a sentence to precise the definition of the volatile organic compound in order clear why it is interesting to use these soil measurements. We also added VOC abbreviation in the section Abbreviations.

Powered by Editorial Manager® and ProduXion Manager® from Aries Systems Corporation

	Figure 1: Change "Functional inferance" to "Functional inference"<br>As requested, we did the correction in the figure 1.<br><br>Figure 2: Add data for most recent years?<br>Yes, we fully agree, it is not intended and we added data for 2018, 2019 and 2020 years in the figure 2.<br><br>Figure 9: Change "Unknow or few data" to "Unknown or insufficient data"<br>As requested, we did the correction in the figure 9.<br><br><br>Reviewer: 2<br>Reviewer #2: The authors presented a review about inferring microbiota functions from taxonomic genes. In general, this topic is very important to gain more insights from amplicon based sequencing strategies to decipher the role of the microbiome. The manuscript is clearly written and the authors present nicely the state of current tools. I have just a couple of minor comments that should be addressed.<br><br>First of all, we want to thank the Reviewer 2 for these commentaries and he pinpoints some drawbacks to help us improving it.<br><br>The specific corrections, and modifications advised by the Reviewer 2 will be highlighted in Blue in the manuscript.<br><br><br>I wonder whether the author can mention PICRUSt2 earlier in the manuscript as it is a great improvement compared to v1. I would suggest to add one sentence in the section about PICRUSt v1.<br>As requested, we have added sentence to describe the main improvements of PICRUSt2 following the paragraph of PICRUSt1 (L271).<br><br>[L176] @MInter should be spelled correctly.<br>As requested, we did the correction in the sentence.<br><br>[L224] Picrust should be written PICRUSt<br>As requested, we did the correction in the sentence.<br><br>[L421] The author write 'No tool...' but I guess it should be 'The tool...'<br>Indeed, it is a clerical error. We did the correction in the sentence.<br><br>[L508] A space is missing ('...fungi - along with...')<br>As requested, we added the space.
**Additional Information:**	
**Question**	**Response**
Are you submitting this manuscript to a special series or article collection?	No
**Experimental design and statistics**<br><br><br>Full details of the experimental design and statistical methods used should be given in the Methods section, as detailed in our Minimum Standards Reporting Checklist. Information essential to interpreting the data presented should be made available in the figure legends.	Yes

Powered by Editorial Manager® and ProduXion Manager® from Aries Systems Corporation

Have you included all the information requested in your manuscript?	
**Resources**<br><br>A description of all resources used, including antibodies, cell lines, animals and software tools, with enough information to allow them to be uniquely identified, should be included in the Methods section. Authors are strongly encouraged to cite Research Resource Identifiers (RRIDs) for antibodies, model organisms and tools, where possible.<br><br>Have you included the information requested as detailed in our Minimum Standards Reporting Checklist?	Yes
**Availability of data and materials**<br><br>All datasets and code on which the conclusions of the paper rely must be either included in your submission or deposited in publicly available repositories (where available and ethically appropriate), referencing such data using a unique identifier in the references and in the "Availability of Data and Materials" section of your manuscript.<br><br>Have you have met the above requirement as detailed in our Minimum Standards Reporting Checklist?	Yes

Manuscript with reviewing Click here to access/download;Manuscript;final_manuscript.docx

Inferring microbiota functions from taxonomic genes: a review

Christophe Djemiel[1], Pierre-Alain Maron[1], Sébastien Terrat[1], Samuel Dequiedt[1], Aurélien Cottin[1], Lionel Ranjard[1]

Authors' affiliations

[1] Agroécologie, AgroSup Dijon, INRAE, Univ. Bourgogne, Univ. Bourgogne Franche-Comté, F-21000 Dijon, France.

Corresponding author

Correspondence to Lionel Ranjard: lionel.ranjard@inrae.fr

Abstract

Deciphering microbiota functions is crucial to predict ecosystem sustainability in response to global change. High-throughput sequencing at the individual or community level has revolutionized our understanding of microbial ecology, leading to the big data era and improving our ability to link microbial diversity with microbial functions. Recent advances in bioinformatics have been key for developing functional prediction tools based on DNA metabarcoding data and using taxonomic gene information. This cheaper approach in every aspect serves as an alternative to shotgun sequencing. Although these tools are increasingly used by ecologists, an objective evaluation of their modularity, portability and robustness is lacking. Here, we reviewed one hundred scientific papers on functional inference and ecological trait assignment to rank the advantages, specificities and drawbacks of these tools, using a scientific benchmark-

ing. To date, inference tools have been mainly devoted to bacterial functions, and ecological trait assignment tools to fungal functions. A major limitation is the lack of reference genomes – compared with the human microbiota –, especially for complex ecosystems like soils. *In fine*, we explore applied research prospects. These tools are very promising and already provide relevant information on ecosystem functioning, but standardized indicators and corresponding repositories are still lacking for them to be used for operational diagnosis.

Keywords

Microbiota; Metabarcoding; Taxonomy; Functional inference; Ecological traits; Soil

1. **Background**

Microorganisms are present in all habitats on Earth and are essential for animals, plants, and therefore for the sustainability of human activities [1]. The extraordinary diversity of microbial communities plays an essential role in the various biogeochemical cycles, allows aquatic and terrestrial ecosystems to function properly and ensures their ability to provide ecological services (*e.g.,* soil structuring, organic matter renewal, nutrient recycling, pollution control, regulation of / barrier to pathogens, or even plant productivity) [2–4]. Their fabulous capacity to adapt to different environmental stresses over time is now well known, and the regulation process of their diversity is better and better deciphered. Despite these tremendous improvements in the approaches targeting indigenous microbiotas, our understanding of the link between microbes and their associated functions remains limited [5]. A workshop hosted by the British Ecological Society's Microbial Ecology Special Interest Group (June 2016) recently identified fifty important research questions in microbial

ecology. One of the main ones was “What methods can we use to marry microbial diversity with function; how do we link transcriptomics, proteomics and metabolomics?" [6]. This sums up the future challenges facing the scientific community when it comes to improving our understanding of the regulation of the microbiome diversity and functions [7].

Microbial functions can be characterized from genomic, proteomic or metabolic data (Fig. 1) [8–10]. Considering genomics, quantitative PCR (qPCR) and microarrays were the first technologies used to describe functional genes or taxa from complex environmental samples [11]. Initially designed to determine the absolute copy number of a single given gene, the latest technical advances can analyze thousands of combinations of samples and targets in parallel [12]. Standardized methods even make it possible to quantify genes of interest (*e.g.,* involved in biogeochemical cycles, pesticide degradation, etc.) to estimate soil quality [13]. DNA microarrays were the first high-throughput technologies giving access to gene expression profiles at the individual or community levels [11,14]. There exist different kinds of microarrays (*e.g.,* PhyloChip, GeoChip; PathoChip; StressChip; CAZyChip). They provide a snapshot of microbial diversity (bacteria, fungi, viruses) and / or of the functional genes present in a given sample (*e.g.,* genes coding for enzymes involved in polysaccharide degradation) [15–18]. Some of these microarrays have become diagnostic tools in many fields, in particular for targeting viruses, bacterial or fungal pathogens or harmful organisms [19]. More recent and cheaper, various high-throughput sequencing (HTS) alternatives have been developed to explore microbial communities (Fig. 1) [20]. Genome and metagenome sequencing have changed the microbial ecology field: thanks to genome sequencing and meta-omics approaches, gene catalogs can be assessed, and new microorganisms can be discovered [21,22].

For example, by implementing a metabarcoding approach, microbial ecologists were first very enthusiastic about such huge taxonomic information, but quickly pointed out the lack of associated functional information [22]. Taxonomic profiles can indeed change to varying degrees among samples, and predicting to what extent these changes impact the overall functional capacity of the community has remained a technical and scientific challenge to date [6,23,24]. Metabarcoding may well be used to directly target functional genes and classify them by taxonomic group, but applications remain limited to a few families [25–29]. In the face of these limitations, two solutions have emerged to indirectly obtain functional information from taxonomic profiles, *i.e*. (i) functional inference, and (ii) ecological trait assignment, using (meta)genome and microbiome big data (Fig. 1). Functional inference predicts the putative functions (*e.g*., gene catalogs, metabolic pathways) of microbial communities, while ecological trait assignment directly retrieves a trait common to all taxa by linking taxonomic names with a dedicated database. The major difference between these two solutions for obtaining functional information is that functional inference retrieves functions even for OTUs without a taxonomic name thanks to phylogenetic placement of sequences (taxonomic markers) in a reference tree and different evolutionary models.

Many bioinformatic tools have been developed since the first publication about a functional prediction tool using metabarcoding data. To date, only one review has addressed functional inference tools; it is focused on aquaculture and on a limited subset of all the tools available to predict functions from 16S rDNA metabarcoding datasets [30]. Therefore, in the present context where new solutions are proposed regularly to predict putative function profiles, the state of the art needs to be scrutinized more exhaustively to build a scientific and technical benchmark. More precisely, we

provide a detailed description of each tool and evaluate their advantages, specificities and drawbacks by paying special attention to their methods, modularity, portability, and robustness. One of the main objectives of this review is to provide a rationale on the use of the different tools currently available for prokaryote and fungal communities and draw perspectives, with a few suggestions to enhance their usefulness in microbial ecology. Finally, we illustrate the application of these methods with studies focusing on the soil environment. The choice of this particular system is justified by the fact that it is the most diverse and complex one in terms of microbial diversity, ecology and functional reservoir [4,31]; therefore, it represents the most challenging environmental matrix for linking diversity and functions. We believe that this work will help scientists working on microbial communities make choices to best take advantage of their high amount of microbial data. This work also shows that although those approaches are promising, they still need improvements to make them operational tools for microbial diagnosis. Repositories using standardized and robust metrics are still lacking when it comes to interpreting the results.

2. Historical and recent increase of microbial datasets

The emergence of HTS in the mid 2000's generated a huge volume of data, leading to a revolution in our way of describing biodiversity. This rise of microbial data can be directly linked to the improvement of high-throughput sequencing technologies, concomitantly with a tremendous drop of sequencing costs (Fig. 2). This was reflected, with a small time lag, by an increase in the number of sequence read archives (SRAs) linked to metabarcoding data deposited on the NCBI website (Fig. 2).

Thanks to the contribution of ecologists, microbiologists, taxonomists and computer scientists, the databases are continuously enriched and are key to enhance our

knowledge about the description and determinism of environmental and human microbiotas [32,33]. For example, the 16S rDNA sequences data available to analyze bacterial/archaeal diversity was multiplied by 4 and 10 in the RDP and SILVA databases, respectively, between 2007 and 2019 (Fig. 3A). The trend is the same for fungal diversity, with a doubling of ITS sequences in the UNITE/INSD database within the last five years (Fig. 3B). 16S rDNA sequences are much more numerous than ITS sequences. However, there were 30 times more fungal species referenced than bacterial ones in 2017 (Fig. 3A, 3B). The numbers of microbial genomes available, in particular in the JGI platform, have increased continuously, and they outpaced Moore's Law mostly from 2013 for bacteria and archaea (Fig. 3C, 3D).

The number of known microbial genes, enzymes or metabolic pathways available in specialized databases has also considerably increased in the last few years [34–36]. Thousands of functional information files are currently accessible in the KEGG, CAZy or MetaCyc databases (Table 1). A recent survey predicted the total global estimated bacterial and fungal functions based on KEGG Orthology to reach 35.5 and 3.2 million, respectively [37]. The authors also indicated that only a tiny fraction of these functions is known today, representing 0.02% and 0.14% for bacteria and fungi, respectively. Although the characterization of gene catalogs using metagenomic approaches was recently criticized [38], the number of non-redundant genes provides an overview of the potential functional reservoir available across various ecosystems [39]. The soil by far appears to harbor the largest pool of functions, followed by the marine, and then animal microbiomes (Fig. 4).

The rapid growth of available genomes is a unique opportunity to predict the putative microbial functions from metabarcoding data by linking taxonomic markers (*i.e.,* rRNA gene amplicons) and their reference genomes or ecological traits. Therefore, the next

section is devoted to the different tools and databases dedicated to functional inference and ecological trait assignment for bacterial and fungal communities.

3. **Overview of the available tools for predicting the potential functions of the microbiotas**

HTS and the presently increasing collection of functional or ecological traits on a more regular and rigorous basis are promising cues for linking biodiversity and associated functions in the near future [24,40]. In the literature, the term "function" is used in different ways depending on the study model, the time scale, or even the habitat [41–44]. The notion of function may refer to genes, enzymes, or metabolic pathways, but may also represent ecological traits that bring together phenotypic and biochemical notions [45–47].

Based on the analysis of twenty papers since 2013, we classified the databases and tools according to the granularity of the results (Fig. 5A), from general information such as ecological traits to more detailed information such as genes or metabolic pathways (Fig. 5). The tools used to obtain fine results, *i.e.,* at the metabolic pathway or gene levels for any taxonomic resolution, are known as functional inference tools (Fig. 5B). On the other hand, we grouped existing tools or databases under the term “ecological trait assignment” when functional information referred to phenotypic or ecological traits and was accessible only for a specific taxonomic rank (Fig. 5C). Indeed, there is a wealth of information often linked to ecological traits in published scientific articles, or of partially formatted metadata (*i.e.,* partial taxonomy or data not linked to the ID of a taxonomic database) [48].

Tools or methods exist, known under the term “text mining”, to automatically collect data from various sources (*e.g.,* a website, a document in pdf format) through

automatic language processing (*e.g.,* natural language processing (NLP)) [49]. For example, @MInter [50] retrieves information related to microbial interactions from abstracts of papers thanks to a supervised machine learning model. Other tools are based on ontologies, *i.e.,* they use a structured set of terms and concepts from a particular domain by specifying the relationships between these terms and their properties, and thus have a common reference for the use of a common vocabulary. For example, OntoBiotope [51] ontology in the food field retrieves the phenotypes and habitats of microbes from the literature based on the NCBI taxonomy. Another ontology exists, called Ontology of Microbial Phenotype [52]; it brings together a structured set of terms and concepts around microbial phenotypes, and specifies the relationships between these terms and their properties. Tools also based on machine learning such as ProTraits [53] can automatically annotate prokaryotic species based on phenotypic or genomic data from scientific articles or online resources (http://protraits.irb.hr).

To date, we have recorded about twenty tools or databases that retrieve functional or ecological data from microbial taxonomic markers, with two to four developments *per* year (Fig. 6 and Table 2). The timeline shows that most of these tools (18/23 in total) are only dedicated to bacteria/archaea, two are dedicated to bacteria/archaea + fungi, and only three are specifically dedicated to fungal organisms. It is important to also underline that most of these tools are devoted to functional inference (13/23). The most cited tool is PICRUSt v1 [54], which remains on top of all others with more than 4,000 citations in 2020. While FUNGuild [55], Tax4Fun v1 [56] or FAPROTAX [57] are reasonably cited with a few hundred citations, the others are very less so with only a dozen citations (Fig. 7A). Interestingly, the articles citing functional inference and ecological trait assignment tools fall within the same scope as the scopes for which they were initially developed (Fig 7B.): PICRUSt, FUNGuild and PAPRICA are mainly

cited in papers about human health, the soil and the marine environments, respectively.

3.1. **Functional Inference**

3.1.1. Definition

Functional inference consists of predicting the functional potential of a microbial community from metabarcoding data. The functional potential of a taxon or of a microbial community represents the metabolic capacities based on the presence / absence of genes involved in these pathways. Functional inference methods are based on the assumption that phylogenetic information from marker gene sequences correlates well enough with the genomic content to produce accurate predictions when associated reference genomes are available. In other words, it assumes a significant relationship between (i) the phylogenetic distance between taxonomic markers and (ii) the conservation of the genetic content, referring to vertical gene descent during the evolution of microbial genomes. This is made possible through the relationship between the phylogenetic relatedness of organisms and their gene content [58,59] (Fig. 5B).

It should be emphasized that the presence of one or more genes involved in a function remains “potential” and may not be expressed under environmental conditions. From this point of view, functional inference results may be similar to shotgun metagenomics data, which is often observed in the literature, especially when focusing on a family of genes or a specific biogeochemical cycle [60]. Also, the fact that inferred metagenomes are based only on the reference genomes available in these tools (archaea, bacteria, fungi), the lateral gene transfer and gene loss cannot be studied unlike shotgun metagenomics.

3.1.2. Available tools

PICRUSt

Phylogenetic Investigation of Communities by Reconstruction of Unobserved States (PICRUSt) v1 [54] is the first tool to have been developed to predict potential functional genes from 16S rRNA metabarcoding and has been the most popular one since it was launched in 2013 (Fig. 5B). PICRUSt v1 needs three things: (i) a reference OTU, (ii) a reference genome, and (iii) a reference phylogenetic tree. As regards the reference OTU, the file (in BIOM or tabulated format) is expected to contain a standard OTU abundance table with sequences picked only against the Greengenes taxonomic reference (18 May 2012 or v13.5/v13.8). This tool based on a modified method of ancestral state reconstruction (ASR) deduces functional information for taxa without a match in the reference genomes. The reference genomes are functional proxies that provide a weighting of the functional profiles for the phylogenetically close taxa within a reference phylogenetic tree. The PICRUSt method is divided into three main steps that are necessary to obtain relevant information on functional profiles: (i) genome prediction, (ii) metagenome prediction, and (iii) analysis of predictions.

The genome prediction step consists of preparing the trees and checking the quality of the input datasets; then comes the reconstruction of ancestral states in the reference tree (ASR, 4 methodologies are available). Using the output files, the software program predicts traits for leaves of the phylogenetic tree lacking sequenced genomes.

During the metagenome prediction step, normalization of the abundance of each OTU is carried out based on rRNA gene copy numbers to predict the functional category abundances of the metagenome. The user obtains an abundance table for each functional category *per* sample. The correcting step of the rRNA gene copy numbers

(GCNs) allows normalizing to correct the biases towards microorganisms with greater GCNs and improve the estimation of microbial diversity [61]. This step is recommended when the OTUs are phylogenetically closely linked to the genomes [62]. To assess the robustness of the predictions, *i.e.,* to obtain the representativeness of the database towards a community of interest, a nearest sequenced taxon index (NSTI) is generated for each sample. It is calculated using the average of the branches that separate the sequences of interest (OTUs, ASVs) in a sample from the reference microbial genome, with a weighting by their relative abundance in the sample. This confidence score is one of the major strengths of this tool. Regarding functional categories, information can be obtained at different levels (genes or metabolic pathways) with more or less detailed descriptions (EC numbers, KEGG pathway [35], COG). Information about all functional categories can also be obtained for each OTU. The last step consists of analyzing the predicted data. This step is essential for interpreting the large number of results generated from a robust statistical analysis.

The major strength of PICRUSt v1 lies in its evolutionary models that infer functions for the complete bacterial community. The portability of this tool with the support of a broad stakeholder community including a forum (google group), blogs, are advantages that make it a central tool for functional predictions (Table 2). Despite all its benefits, PICRUSt v1 has drawbacks such as focusing only on the 16S rDNA marker and using only Greengenes taxonomy (Table 2). Several specialized tools have emerged to integrate PICRUSt as a sub-layer in order to carry out diagnoses in the medical field [63] or directly in a pipeline [64]. PICRUSt v2 fills the gaps of the first version, with an improvement that allows inference directly based on the sequences and no longer through taxonomy. Another improvement concerns the addition of bacterial but also

fungal reference genomes, thus making it possible to infer from 18S rDNA and ITS amplicons [65].

PAPRICA

Pathway Prediction by Phylogenetic Placement (PAPRICA) [66] infers the metabolic potential of prokaryotic and eukaryotic communities from metabarcoding data based on rRNA gene amplicons. It was the first tool that allowed for the functional prediction of 16S and 18S rRNA amplicons. It comes in the form of a pipeline taking the OTU reads as inputs to place them in an rRNA reference tree built from complete genomes. To build this tree, a consensus genome is found for each node in the tree, which then makes it possible to predict metabolic pathways for the sequences of interest without a match in the complete reference genomes. The abundance of metabolic pathways is weighted by rRNA gene copy numbers from known genomes. A strength of this tool is that it also provides an indicator of genomic stability depicting the robustness of the results. However, PAPRICA, like all the tools using a reference phylogenetic tree and sequence placement methods, is dependent on the quality of rRNA resolution, and this represents a drawback when some clades may be affected (Table 2).

Tax4Fun

Tax4Fun [56] is an R [67] package published in 2015 for predicting functional profiles from targeted metagenomic 16S rRNA data. However, the algorithm and statistical efficiency based on a metabolic mixture model in terms of a mixture of pathways (MoP) was developed in 2013. This R-based architecture is inherently a cross-platform tool, and it may be more accessible for a large number of users with low experience in bioinformatics. This tool uses pre-calculated functional profiles like PICRUSt v1 and

taxonomic data formatted from the SILVA database. One of the differences with PICRUSt the rRNA sequence placement in the reference genomes, which is achieved by a BLAST search (instead of a tree placement approach for PICRUSt). It is a very convenient tool because it provides a confidence score (FTU and FSU) to determine the fraction of OTUs that was not mapped to KEGG organisms or the number of sequences without KEGG Orthology (KO) hits (Table 2). Like PICRUSt v1, it cannot be used for fungal diversity predictions.

Piphillin

Piphillin [68] differs from the PICRUSt or PAPRICA approaches because it does not use a phylogenetic tree or database (16S) but directly maps the OTU sequences on the rRNA of the reference genomes using a nearest-neighbor algorithm. This specificity could avoid faulty sequence placements in the reference phylogenetic tree. It is used online only, which represents both a strength and a weakness: it benefits from computing power (a strength), whose strength depends on the hosting server (*e.g.* quota management, cluster configuration) (a weakness). A Piphillin sub-layer also exists to complete the analysis of the results [69].

The quality of prediction represents a prerequisite for the application of the above-presented tools to study indigenous microbial communities. It may depend on the tool, but also on the type of targeted ecosystem. To test the quality of functional prediction according to the tool and the studied ecosystem, we compiled the NSTI scores for PICRUSt v1 and the FTUs for Tax4Fun from a subsampling of articles that covered a range of ecosystems – human, marine, plant, and soil (Fig. 8). Whatever the tool, the best predictions were obtained for the human microbiotas, and the most approximate

ones for the soil samples. The variability of quality scores across the different soil studies seemed to be lower with PICRUSt than with Tax4Fun. Nevertheless, some soil studies using Tax4fun indicate a good-quality survey with only about 30% of OTUs unmapped to a reference. This likely reflects the discrepancy between human reference genome availability and soil microbiota genome availability. In addition, microbial diversity is much more complex in soils than in the human microbiotas. In this case, it is essential that the quality scores from functional inference tools should be taken into account because it is a key to a robust interpretation of the results. Unfortunately, we found few studies indicating these quality scores.

3.2. **Ecological trait assignment**

3.2.1. Definition

Ecological trait assignment differs from functional inference since it consists of obtaining information on the life strategy, phenotypic and quantitative genomic traits (*e.g.,* trophic modes, growth strategy) of a taxon from its nomenclature, whatever its taxonomic rank. If the taxon is not present in the database, it will not be possible to know its traits (Fig. 5C). This approach is faster than functional inference for retrieving an item of functional information, but tools dedicated to metabarcoding outputs are lacking, and only a few ecological traits are available (Table 2). The main interest is to get functional information with a possibly not so fine granularity as functional inference does, but obviously more accurate. Ecological traits are indeed often based on results with biochemical experimentations from curated databases or scientific publications. Practically speaking, only the guild will be recovered and for example the fungal sequences identified as belonging to the *Serpula* genus will be assigned to a wood

saprotroph when an ecological trait tool is used; with an inference tool, the abundance of various genes related to polysaccharide degradation will be attributed to all fungal sequences.

3.2.2. Tools

FUNGuild

FUNGuild [55] is the pioneer and one of the few tools that assigns ecological traits to fungi based on their taxonomy (Table 2). These assignments rely on metabarcoding data. They require providing a contingency table (OTUs or sequence counts *per* sample) and the link between each OTU and its taxonomy. To carry out the assignment, FUNGuild uses its own curated database, and searches it for the taxon. This database contains several taxonomic levels (*e.g.,* phylum, genus, species). However, the taxonomic name at the genus or species level is necessary to assign traits to the taxa of interest. Trait information is available in 66% of the cases at the genus level, and only in 34% of the cases at the species level [55]. The user obtains a summary table of the different possible ecological traits for each taxon with a robustness indicator and a confidence range ("possible", "probable", and "highly probable").

The strength of this database is that the provided data are based on the literature (primary research), or on reference websites or their own collective research experience if the datum is missing. The authors recommend the use of the UNITE database for taxonomic assignment and therefore the use of the internal transcribed spacer (ITS) marker, but it can be easily transposed to data based on the 18S rRNA marker. It just requires creating a wrapper to make a link between the taxonomy of the data and FUNGuild to retrieve the traits of interest.

A new database called Fun^{Fun} [70] is now available. It encompasses 80 fungal ecological traits. In reality, this database is a FUNGuild database overlay with information on genetic, enzymatic, morphological, stoichiometric, life history, and physiological aspects. In addition, the authors mention that Fun^{Fun} will be updated in terms of taxonomy and associated guilds, which is not necessarily the case with FUNGuild. However, although this database is promising, a lot of information is missing because it integrates literature data for the first time ever, and its improvement relies on the progress of research as well as the contribution of scientists. This caused an impulse leading to a community of scientists proposing a new database: FungalTraits [71] links information from FUNGuild and Fun^{Fun}. It is very complete, and offers different levels of life styles. Please note that this database includes species from the fungal kingdom but also fung-like stramenopiles (*e.g.,* the Oomycota phylum). This may be especially useful because various species are identified as major plant pathogens within Oomycota. For example, the genus Phytophthora gathers several crop pathogens causing important losses and can represent a risk to global food security [72].

To conclude, the minor drawbacks of FUNGuild, with rare updates or a tool oriented to ITS sequences, have been offset by the new Fun^{Fun} and FungalTraits databases.

To complete the tools concerning fungal communities, DEEMY [73] is an information system only available online and specialized in ectomycorrhizas (http://www.deemy.de). This website references 554 species associated with their respective symbiotic organisms, including 104 genera. To characterize each species, a summary sheet provides taxonomic nomenclature, bibliographical references and photographs, as well as information on morphology, anatomy, potential chemical reactions, or even ecology traits.

FAPROTAX

Functional Annotation of Prokaryotic Taxa (FAPROTAX) [57] is used to assign metabolic functions, ecological traits or large functional groups relevant to prokaryotes (Table 2). This database was built manually from the scientific literature of the *International Journal of Systematic and Evolutionary Microbiology* (IJSEM) and Bergey's *Manual of Systematic Bacteriology*. It contains about 4,700 unique prokaryotic taxonomies (mostly at the species level) and 90 functional groups. FAPROTAX is based on the implicit assignment of a trait / function to a taxon (whether cultivated or not) if all the cultivated members display this trait / function. Its main limitation is that it is focused on marine prokaryotic organisms, so that communities from other biomes can be missing. Another point to be considered is that if the taxa of interest do not have a species name, the tool cannot draw inferences at the upper levels (*e.g*., genus) to assign an ecological trait.

IJSEM phenotypic database

IJSEM [74] compiles phenotypic and environmental tolerance data about more than 5,000 bacterial strains. It is an official and unique reference for publishing and validating new strains. These strains cover about 23 phyla from various habitats (mainly soils). The database appears as a TSV file (https://figshare.com/articles/International_Journal_of_Systematic_and_Evolutionary_Microbiology_IJSEM_phenotypic_database/4272392), and available information can be grouped into five categories: ancillary data (*e.g.*, article’s digital object identifier; taxonomic nomenclature), morphology/phenotype (*e.g.,* Gram stain status; motility),

metabolism (*e.g.*, BIOLOG information), environmental preferences (*e.g.*, habitat of isolation; oxygen requirement), and sequence data (*e.g.*, 16S rRNA accession no.).

BacDive

BacDive [75] is one of the largest metadatabases (https://bacdive.dsmz.de) referencing information on bacterial and archaeal diversity (Table 2). The tool links taxonomy and phenotypic information directly but the database can only be browsed on a website or data can be downloaded from it. However, it provides a complete application programming interface (API) to achieve scripts and retrieve the desired information. In the first months of 2020, it offered data on 81,827 bacterial and archaeal strains, including 14,091 type strains, and thereby covered approximately 90% of the described species according to their website. This database is very interesting because it provides different levels of robust information on taxonomy, morphology, physiology (API®-tests), molecular data, and cultivation conditions. As for physiological data, it provides – for example – the main substrates used for culturing a species and the enzymes present (a link with the EC classification number is available). These data have been more broadly incorporated into a tool (bacteria-archaea-traits) that encompasses numerous traits of bacteria and archaea from 26 sources [46].

To complete this list, a few specialized databases target only one or a few traits. For example, Engqvist [76] recently grouped the growth temperatures of 21,498 non-redundant organisms across the whole tree of life. This study showed a strong correlation between the growth temperature of organisms and enzymatic optima, with temperature-dependent increases or decreases of enzymatic functions. This information can be very interesting and complementary to the interpretation of

functional inference results, and can be linked – for example – to environmental conditions.

4. Application of these new approaches to the functions of the soil microbial ecosystem

4.1. Functional Inference

In recent years, meta-omics approaches have been increasingly included in soil monitoring, whether in fundamental research programs or in more operational projects [77]. Most studies (about 60% based on keywords in the titles or abstracts of the publications, see Fig. 7B) have focused on PICRUSt to generate functional predictions from taxonomic data of the soil microbiota. We summarized the most valuable outcomes about soils by grouping them into categories: anthropogenic gradient, agricultural practices, and biogeochemical cycle or soil properties (Fig. 9). For example, a study showed that plant-bacteria interactions in the rhizosphere were mainly related to beneficial cooperation [78] involving the release of root exudates by the plants on the one hand, and hormone production or the ability to break down toxic chemicals by bacteria on the other hand. Another study investigated the stoichiometric regulation of soil carbon cycling by comparing functional predictions by metabarcoding (*via* PICRUSt) and shotgun sequencing on a wide C:N:P soil gradient in a rice field [60]. A strong correlation was evidenced between the functional predictions from metabarcoding and metagenomics as regards the abundance of some metabolic families involved in the C, N and P cycles. Still using PICRUSt, another study examined the effects of intercropping by predicting the soil microbial functional profiles. It evidenced that an intercropping system increased the functional potential in terms of carbon fixation pathways and the citrate cycle [79]. Finally, a study focused on the

impact of long-term land-use practices (forest, grassland, crops) on soil bacterial communities [80] showed that forest soils harbored the largest reservoir of genes, followed by no-till soils and then grasslands. The plowed soils presented the lowest functional richness.

Based on Tax4Fun predictions, a study investigated the impact of different irrigation practices with various water qualities (freshwater, treated or untreated wastewater) along with the different land use systems in drylands [81]. The authors compared the potential functional and taxonomic profiles of bacteria. Irrigation with wastewater had an effect on bacterial responses by shaping communities and functional profiles. By bringing more nitrogen, wastewater favored the response of certain genera, in particular Nitrosospira, and increased the relative abundance of the genes involved in nitrification and denitrification.

Among all the functional inference tools available today, two of them stand out, *i.e.,* PICRUSt and Tax4Fun. A benchmark study of these tools found no major differences in terms of performance, especially for soil samples [82]. Another benchmark study indicated that these two tools provided similar functional profiles but could be complementary for certain gene families found only in one or the other [83]. Moreover, the characterization of the fungal functional potential by PICRUSt2 is too recent for us to have any insights into its robustness concerning soil communities. Compared to trait assignment, the links between diversity and functions still remain tenuous concerning certain biogeochemical cycles or the impact of climate change and plant diversity (Fig. 9).

4.2. Ecological trait assignment

The complexity of microbial traits is variable, with simple traits like organic phosphate utilization, and more complex ones like methanogenesis [24,84]. The conservation of prokaryotic traits or core genes varies according to phylogenetic depth [58]. For example, the complex methanogenesis trait appears to be very conserved at the order and family levels, while contrastingly with the resistance to specific bacteriophages appears to vary at the species level due to particular point mutations [24]. Below are a few examples of the possible benefits of ecological traits to the analysis of the diversity of soil microbial communities (Fig. 9).

Regarding the assignment of fungal traits, FUNGuild is currently and by far the most implemented tool, if not the only tool implemented by ecologists wishing to supplement their diversity analyses with data on the ecological traits of fungal communities, and mainly in studies on soil fungal communities [85–88]. A study on fungal communities in subtropical forest soils highlighted a negative relationship between the abundance of pathogenic fungi and the phylogenetic diversity of plant communities [89]. Another study showed a positive correlation between soil fungal community dissimilarities (plant pathogens, saprotrophs and ectomycorrhizas) and plant phylogenetic distances in forest soils [90]. Tropical land uses also impact the functional guild. A massive shift of fungal trophic modes has been showed – notably a decrease in mycorrhizal fungi and an increase in saprophytic and pathogenic fungi – along with increased anthropization levels [91]. Interestingly, several large-scale (national or global) studies have characterized the distribution of trophic types while identifying the environmental parameters that influence them [85,92–94]. The distribution of these trophic modes seems to vary greatly depending on temperature and precipitation [94]. This supports a recent global study focused on the distribution of pathogens and indicating higher abundance in warm regions [93]. A recent study compared the trophic modes

(synonym: life strategies) assigned to the ITS and 18S rDNA molecular markers by FUNGuild [85]. This study indicated that the saprotroph and pathotroph richness levels were directly and negatively correlated with the organic matter content and elevation, and positively correlated with the pH and bulk density. For symbiotroph richness, the relationship differed depending on the molecular marker used: it was positively correlated with the C:N ratio when ITS sequences were used, but negatively correlated when 18S rDNA sequences were used. Similarly, the pH was positively correlated based on 18S rDNA data, but negatively correlated based on ITS data [85]. These differences may come from the fact that the two molecular markers do not cover the same taxonomic range. Therefore, the choice of molecular markers and primers is essential because it impacts the global picture obtained by possibly enhancing or decreasing the representation of particular functional groups in the community. For example, arbuscular mycorrhizal fungi are better represented, in particular the *Glomeromycota* group, when the 18S rDNA marker is used [95,96]. A study at a smaller scale also showed that saprotroph richness was directly driven by the soil physico-chemical parameters and confirmed the results mentioned above. The authors showed a positive correlation with the pH but a negative one with the C:N ratio [97]. All these studies used the FUNGuild tool dedicated to characterizing fungal community traits.

Regarding the assignment of bacterial traits, various databases exist but few tools have been developed to assign ecological traits from metabarcoding datasets. Only FAPROTAX stands out as a powerful tool for analyzing the functional potential of soil communities [98], although it is dedicated to marine organisms.

5. Technical and conceptual limitations and biases

The metabarcoding approaches have significant advantages for characterizing indigenous prokaryotic and eukaryotic microbial communities. Standard protocols now exist, from sample preparation to bioinformatic and statistical analyses, and scientists have acquired an important feedback on biases, costs, and efficiency [99–101].

A fundamental limitation of functional inference tools, represented by gene gain and loss, is mainly due to horizontal gene transfer but also gene duplication, gene loss, and de novo gene birth [102–105], which is addressed in the literature and taken into account to some extent in these tools. However, horizontal gene transfer remains difficult to consider accurately for functional prediction, and its influence on microbial communities is hard to estimate. Moreover, the horizontal gene transfer rate varies substantially within the tree of life and according to gene families / pathways [24,84,102]. This process is mainly described in prokaryotes, but is also found to a lesser extent in eukaryotes, in particular fungi [106]. Microorganisms can gain a function through plasmid transfer, but no information was found in the literature about functional prediction [54]. However, plasmids are extrachromosomal DNA molecules that play a role in the rapid adaptation of microbial communities to environmental changes across all microbiomes [107,108]. In particular, they are transferred between phylogenetically distant populations for them to acquire genes and beneficial traits for their adaptation (*e.g.,* resistance to antibiotics, biocides, pollutants). This is key for all environments, especially soils where biotic and abiotic fluctuations are tremendous [109]. The transfer of plasmids is also introduced from phages or viruses into microbial genomes [110].

From a technical point of view, most of the studies on microbial diversity using metabarcoding approaches are based on the sequencing of one or more hypervariable regions and remain limited by the size of the amplicon to be sequenced. The most

commonly used Illumina sequencing platforms (MiSeq, HiSeq and NovaSeq) can provide maximum readings of 600 bp (~550 bp after adapter/tag/primer trimming). Several studies have questioned the most suitable regions for obtaining the best taxonomic resolution [111,112]; the use of full-length rRNA (~1,800 bp) seems to be the most appropriate solution [113]. It would significantly enhance phylogenetic resolution for prokaryotic and eukaryotic microorganisms [114] (Fig. 10, second box). Short reads do not allow good enough resolution in taxonomic assignment either (*i.e.,* not down to the species level) although this point is crucial for placing sequences/taxa in the phylogenetic tree to achieve functional inference. With third-generation HTS platforms (*e.g.,* PacBio, Oxford Nanopore), full-length molecular markers can be sequenced, *e.g*., 16S/18S rRNA genes or the full ITS1 and ITS2 sequences [115,116]. This will considerably improve taxonomic assignment, and make it possible to assign sequences at the species or even the strain level in certain cases [116]. This way, functional inference and ecological trait assignment will be improved. However, if the objective is to obtain the best taxonomic resolution possible, the study of ecological traits at high taxonomic ranks (*e.g.,* the phylum) remains very promising, especially for highly conserved traits [117]. For example, the carbon mineralization rate was positively (*e.g.,* Bacteroidetes) or negatively (*e.g*., Acidobacteria) correlated with their relative abundance [118].

A good practice complementary to the use of full-length amplicon sequencing would be the use of amplicon sequence variants (ASVs, also called ZOTUs) to increase the rate of inference with a better sequence placement on the reference tree [65,119]. Indeed, for those using an OTU clustering approach with a similarity threshold, one solution would be to use all the sequences within the OTUs instead of one

representative sequence for each OTU seed, which could be less accurate. However, this would also increase the analysis time.

6. Importance of taxonomy and genome references: from accuracy to resolution

Many tools use taxonomic data to obtain information about microbial functions through a metabarcoding approach. Therefore, it is very important to check the bioinformatic strategy used to analyze the amplicon sequences, from the filtering steps to OTU clustering or not (see ASV), including taxonomic assignment.

The use of tools on ecological traits is highly dependent on taxonomic resolution. For example, when using FUNGuild, special attention must also be paid to the fact that a sequence assigned at the genus level may be associated with several trophic types, and that plant-pathogenic fungi are highly host-specific and may be non-pathogenic in the context of the study. For the sequences (or OTUs) without any taxonomic assignment, functions cannot be obtained using tools on ecological traits (Fig. 10, second box). In order to improve this point, especially for fungal communities, inferences may be drawn based on phylogeny, as done for bacteria, archaea or macroorganisms [120–124]. One of the avenues to be explored is the use of ASR tools such as PICANTE [125] or CASTOR [126], which infer traits for taxa devoid of ecological data from a phylogenetic tree.

Functional inference tools depend on the reference genomes to establish predictions, so that the accuracy of the results can vary among samples. Samples with well described host-associated communities such as the human microbiome have many reference genomes available, and allow good predictive accuracy (Fig. 8, Fig. 10 third box). Contrastingly, in more complex and highly biodiverse environments like soils [127], the genomes representing the total taxonomic diversity are much more difficult

to obtain. The proportion of cultivable terrestrial strains remains very low (approximately 25%) compared to the human microbiotas (80%) [128]. Thus, the results estimated for the communities from complex biomes are approximate and debatable.

In order to improve functional prediction results, it is advisable to provide genomes specific to the habitat of interest [129]. Considerable efforts have to be made to increase the number of habitat-specific reference genomes (animal / human, water, plant, soil), with special attention to the most complex and unknown environments [130]. Tools to routinely update the databases will also need to be developed [131]. This is an ongoing dynamic at the international scale. For example, the annotation of reference genomes in databases is not yet representative of soil microbial diversity [132]. To fill this gap, an effort has been made by creating the Refsoil database (which does not seem to be maintained (https://github.com/germs-lab/ref_soil)) [132] or a Refsoil + plasmid database [108].

7. **Discussion and future prospects**

The possible retrieval of a putative functional potential or ecological traits directly from taxonomic markers and metabarcoding approaches opens new perspectives for our understanding of microbial communities, both from a fundamental and/or operational point of view (*e.g.,* functional redundancies, diagnostic tool) [63,133]. This information can be used to (i) understand the main functions potentially expressed in a given environment and identify the possible drivers, (ii) examine the distribution of functions among taxonomic group, or (iii) supplement the classical diversity metrics used to evaluate the ecological state of environmental matrices (Fig. 10, first box). Beyond providing an overview of the putative functions of an ecosystem, prediction

tools could also provide more detailed information than taxonomic markers do for users to significantly distinguish sample groups from each other in certain habitats [113] (Fig. 10A, first box).

A new generation of tools solves the main limitations of the previous generation tools by including improvements in terms of taxonomic marker targeting, methodology and flexibility.

Future prospects with second-generation tools

Second-generation tools are currently emerging, *e.g.* PICRUSt2 [65], Tax4Fun2 [129] or iVikodak [134] (Fig. 6). Indeed, Langille's team of developers bridged the gap for the scientific community working on fungal ecology. PICRUSt2 now includes 18S rDNA and ITS amplicons from the fungal kingdom. Another great improvement is flexibility: the sequence can be used directly, instead of taxonomy based on Greengenes nomenclature. Users are no longer dependent on taxonomy to infer functions; this is a great comfort, and provides better robustness of the analyses. However, users should be wary of the results because the number of sequenced fungal genomes currently integrated in the tool is much lower than the number of bacterial genomes. It is recommended to check the quality score (*e.g.,* NSTI) for the robustness of the results and interpretation. However, this limitation can be lifted. For example, the 1000 Fungal Genomes Project [135] is aimed at high-quality sequencing and annotation of fungal genomes so as to build a reference dataset to be used for meta-omics data analysis.

Another downside of these tools is the absence of data support for micro-eukaryotic communities, which are essential to the soil ecosystem. Protists are abundant and diverse, with a large range of functional diversity, and are highly involved in soil food

webs and functioning [136,137]. It would be particularly useful to develop tools dedicated to protists from data on ecological traits available in the literature [138].

Challenges: from fundamental research to diagnosis

Switching from fundamental research to practical applications would be really interesting because although operational microbial diversity bioindicators are increasingly emerging, there is a huge gap in the functional information of microbial communities. Even if the number of species can be an indicator of the impact of biotic and abiotic factors [139,140], the need to characterize the associated functions at the ecosystem level has become obvious to obtain a complete diagnosis with functional information on the soil microbial quality [141,142].

As regards human health, identifying taxonomic and functional changes to estimate the contributions of taxa associated with a disease is an emerging topic [143], as for example in research into gene markers involved in colorectal or oral cancers [144,145]. Some interesting examples exist in the biomonitoring and bioassessment of water quality [146,147] but examples for the soil microbial quality are still scarce. The huge complexity and diversity of the soil microbial community probably still limits such applications to the soil ecosystem, along with a lack of genome references. However, initiatives at the global level are in progress to access the soil biodiversity using taxonomic, functional and environmental data [140]. We can also note that a real dynamic seems to be developing at the international scale to collect, standardize and disseminate traits through the tree of life via an open science tool called the Open Traits Network (OTN) [83].

The huge complexity and diversity of the soil microbial community probably still limits such applications to the soil ecosystem, along with a lack of genome references. However, initiatives at the global level are in progress to access the soil biodiversity using taxonomic, functional and environmental data [148]. We can also note that a real dynamic seems to be developing at the international scale to collect, standardize and disseminate traits through the tree of life *via* an open science tool called the Open Traits Network (OTN) [83].

To our knowledge, providing robust and operational indicators based on putative functions derived from metabarcoding data is impossible today. The main challenges are to (i) aggregate and summarize the mass of data currently generated, (ii) test the predictions on datasets and compare them with “real” functional measurements, (iii) validate these indicators on datasets under diverse experimental conditions (*e.g.,* land use gradient, agricultural practices) at the local and global scales, and (iv) develop representative repositories to ensure the validity of the diagnosis made from these new tools.

Regarding aggregation and data reduction [(i)], a track would be to use a constrained non-negative matrix factorization approach [149], an alternative to the concept of community-aggregated traits (CATs) [150]. This method has already been used to aggregate functional traits from meta-genomes [149]. The authors demonstrated that significant data reduction made it possible to propose simple models to describe a set of complex functions at the scale of an ecosystem (here the potential for fiber degradation in the human intestinal microbiota) while preserving biological data quality [149]. Concerning [(ii)], it will be interesting, for example, to confront functional predictions with volatile organic compound (VOCs) emissions or microbial respiration rates from soil measurements. Indeed, the very diverse microbial VOCs are secondary

metabolites playing various roles in particular making it possible to carry out more or less long-distance interactions and communication (*e.g.,* growth, motility, antibiotic resistance, expression of stress response genes) [151]. Moreover, to suggest these tools as robust indicators of the soil quality [(iii)], it will be essential to use large datasets in order to determine the best metrics (*e.g.,* functional richness, relative gene abundance, aggregation of traits) and the most sensitive genes or groups of genes depending on the various scientific issues. Once these limitations have been lifted, these tools will provide results of great interest to the scientific community at relatively affordable human, technological and financial costs. However, maintaining the associated scientific expertise will be essential to support their transfer for operational applications and avoid erroneous interpretations that could potentially have disastrous consequences for soil users and soil policy makers [(iv)]. For example, interpreting trophic types requires strong expertise, with particular attention to the exploitation of potential pathogenicity information – a highly sensible task. The responses of the traits vary according to the disturbances applied to the ecosystem [152], and the results must be contextualized to ensure correct interpretation.

Conclusion

The exploration of the microbial functional diversity based on taxonomic marker genes in order to improve our knowledge of microbial diversity and functions is just starting. As highlighted in this review, various solutions have emerged over a number of years and are being improved quickly thanks to technological advances. Functional inference results are already robust and representative for some ecosystems with low diversity (specific richness) and with well characterized genomes such as the human microbiotas. Progress now needs to be made for more complex environments. The

upcoming challenge, notably for environmental samples, will be to establish the link between functional predictions on reference datasets and environmental measurements. The new network SoilBON dedicated to monitoring soil biodiversity and functional ecosystems at a global scale, with particular attention to microbial diversity, is a step in this direction [3]. This ambitious framework aims to collect and analyze soil diversity based on soil ecological indicators (*i.e.,* essential biodiversity variables [153]). One purpose of this framework is to inform policy makers and stakeholders for them to adapt measures and preserve this biodiversity.

Abbreviations

DNA: Deoxyribonucleic acid; qPCR: quantitative polymerase chain reaction; HTS: high-throughput sequencing; rDNA ribosomal DNA; rRNA: ribosomal ribonucleic acid; SRA: sequence read archive; NCBI: National Center for Biotechnology Information; RDP: Ribosomal Database Project; ITS: internal transcribed spacer; INSD: International Nucleotide Sequence Database; JGI: Joint Genome Institute; KEGG: Kyoto Encyclopedia of Genes and Genomes; CAZy: carbohydrate-active enzymes; NLP: natural language processing; OTU: operational taxonomic unit; GCN: gene copy number; NSTI: nearest sequenced taxon index; FTU: fraction of OTUs; EC number: enzyme commission number; COG: cluster of orthologous groups; KO: KEGG orthology; IJSEM: *International Journal of Systematic and Evolutionary Microbiology*; API: application programming interface; C, N and P cycles: carbon, nitrogen and phosphorus cycles; bp: base pairs; ASV: amplicon sequence variant; ZOTU: zero-radius OTU; OTN: open traits network; CAT: community-aggregated trait. VOC: Volatile Organic Compound.

Competing interests

The authors declare that they have no competing interests.

Funding

This work was funded by the ADEME (French Environment and Energy Management Agency).

Author contributions

C.D and L.R conceptualized the manuscript. C.D drafted the manuscript with contributions from S.T, S.D, A.C, P-A.M and L.R. All authors read and approved the final manuscript.

Acknowledgements

Thanks to Annie Buchwalter for correction and improvement of English language in the manuscript.

References

1. Cavicchioli R, Ripple WJ, Timmis KN, Azam F, Bakken LR, Baylis M, et al. Scientists' warning to humanity: microorganisms and climate change. Nat Rev Microbiol [Internet]. 2019;17:569–86. Available from: http://www.embase.com/search/results?subaction=viewrecord&from=export&id=L628318774%0Ahttp://dx.doi.org/10.1038/s41579-019-0222-5

2. Maron PA, Mougel C, Ranjard L. Soil microbial diversity: Methodological strategy, spatial overview and functional interest. Comptes Rendus - Biol [Internet]. Academie des sciences; 2011;334:403–11. Available from:

http://dx.doi.org/10.1016/j.crvi.2010.12.003

3. Guerra CA, Bardgett RD, Caon L, Crowther TW, Delgado-Baquerizo M, Montanarella L, et al. Tracking, targeting, and conserving soil biodiversity: A monitoring and indicator system can inform policy. Science (80-) [Internet]. 2021;371:239–41. Available from: https://www.sciencemag.org/lookup/doi/10.1126/science.abd7926

4. Bardgett RD, Van Der Putten WH. Belowground biodiversity and ecosystem functioning. Nature [Internet]. 2014;515:505–11. Available from: http://www.nature.com/articles/nature13855

5. Rivett DW, Bell T. Abundance determines the functional role of bacterial phylotypes in complex communities. Nat Microbiol [Internet]. 2018;3:767–72. Available from: http://www.nature.com/articles/s41564-018-0180-0

6. Antwis RE, Griffiths SM, Harrison XA, Aranega-Bou P, Arce A, Bettridge AS, et al. Fifty important research questions in microbial ecology. FEMS Microbiol Ecol [Internet]. 2017;93. Available from: https://academic.oup.com/femsec/article-lookup/doi/10.1093/femsec/fix044

7. Sergaki C, Lagunas B, Lidbury I, Gifford ML, Schäfer P. Challenges and Approaches in Microbiome Research: From Fundamental to Applied. Front Plant Sci [Internet]. 2018;9:1–12. Available from: https://www.frontiersin.org/article/10.3389/fpls.2018.01205/full

8. Starr AE, Deeke SA, Li L, Zhang X, Daoud R, Ryan J, et al. Proteomic and Metaproteomic Approaches to Understand Host-Microbe Interactions. Anal Chem [Internet]. 2018;90:86–109. Available from: https://pubs.acs.org/doi/10.1021/acs.analchem.7b04340

9. Aldridge BB, Rhee KY. Microbial metabolomics: Innovation, application, insight.

Curr Opin Microbiol [Internet]. 2014;19:90–6. Available from: https://linkinghub.elsevier.com/retrieve/pii/S1369527414000794

10. Knight R, Vrbanac A, Taylor BC, Aksenov A, Callewaert C, Debelius J, et al. Best practices for analysing microbiomes. Nat Rev Microbiol [Internet]. 2018;16:410–22. Available from: http://www.nature.com/articles/s41579-018-0029-9

11. Porter TM, Hajibabaei M. Scaling up: A guide to high-throughput genomic approaches for biodiversity analysis. Mol Ecol. 2018;27:313–38.

12. Mehle N, Dreo T. Quantitative Analysis with Droplet Digital PCR. Notes Greek Text Genes [Internet]. 2019. p. 171–86. Available from: http://link.springer.com/10.1007/978-1-4939-8837-2_14

13. Thiele-Bruhn S, Schloter M, Wilke BM, Beaudette LA, Martin-Laurent F, Cheviron N, et al. Identification of new microbial functional standards for soil quality assessment. Soil [Internet]. 2020;6:17–34. Available from: https://soil.copernicus.org/articles/6/17/2020/

14. Sessitsch A, Hackl E, Wenzl P, Kilian A, Kostic T, Stralis-Pavese N, et al. Diagnostic microbial microarrays in soil ecology. New Phytol. 2006;171:719–36.

15. He Z, Gentry TJ, Schadt CW, Wu L, Liebich J, Chong SC, et al. GeoChip: A comprehensive microarray for investigating biogeochemical, ecological and environmental processes. ISME J. 2007;1:67–77.

16. Lee YJ, Van Nostrand JD, Tu Q, Lu Z, Cheng L, Yuan T, et al. The PathoChip, a functional gene array for assessing pathogenic properties of diverse microbial communities. ISME J [Internet]. Nature Publishing Group; 2013;7:1974–84. Available from: http://dx.doi.org/10.1038/ismej.2013.88

17. Zhou A, He Z, Qin Y, Lu Z, Deng Y, Tu Q, et al. StressChip as a high-throughput tool for assessing microbial community responses to environmental stresses. Environ

Sci Technol. 2013;47:9841–9.

18. Abot A, Arnal G, Auer L, Lazuka A, Labourdette D, Lamarre S, et al. CAZyChip: dynamic assessment of exploration of glycoside hydrolases in microbial ecosystems. BMC Genomics [Internet]. BMC Genomics; 2016;17:671. Available from: http://www.ncbi.nlm.nih.gov/pubmed/27552843%5Cnhttp://www.pubmedcentral.nih.gov/articlerender.fcgi?artid=PMC4994258

19. Tedersoo L, Drenkhan R, Anslan S, Morales-Rodriguez C, Cleary M. High-throughput identification and diagnostics of pathogens and pests: Overview and practical recommendations. Mol Ecol Resour [Internet]. 2019;19:47–76. Available from: https://onlinelibrary.wiley.com/doi/abs/10.1111/1755-0998.12959

20. Franzosa E a., Hsu T, Sirota-Madi A, Shafquat A, Abu-Ali G, Morgan XC, et al. Sequencing and beyond: integrating molecular “omics” for microbial community profiling. Nat Rev Microbiol [Internet]. Nature Publishing Group; 2015;13:360–72. Available from: http://www.nature.com/doifinder/10.1038/nrmicro3451%5Cnhttp://www.ncbi.nlm.nih.gov/pubmed/25915636

21. Segata N, Boernigen D, Tickle TL, Morgan XC, Garrett WS, Huttenhower C. Computational meta’omics for microbial community studies. Mol Syst Biol [Internet]. Nature Publishing Group; 2013;9:1–15. Available from: http://dx.doi.org/10.1038/msb.2013.22

22. Frioux C, Singh D, Korcsmaros T, Hildebrand F. From bag-of-genes to bag-of-genomes: metabolic modelling of communities in the era of metagenome-assembled genomes. Comput Struct Biotechnol J [Internet]. 2020;18:1722–34. Available from: https://linkinghub.elsevier.com/retrieve/pii/S2001037020303172

23. Fierer N. Embracing the unknown: Disentangling the complexities of the soil

microbiome. Nat Rev Microbiol [Internet]. Nature Publishing Group; 2017;15:579–90. Available from: http://dx.doi.org/10.1038/nrmicro.2017.87

24. Martiny JBH, Jones SE, Lennon JT, Martiny AC. Microbiomes in light of traits: A phylogenetic perspective. Science (80-) [Internet]. 2015;350:aac9323–aac9323. Available from: https://www.sciencemag.org/lookup/doi/10.1126/science.aac9323

25. Penton CR, Johnson TA, Quensen JF, Iwai S, Cole JR, Tiedje JM. Functional genes to assess nitrogen cycling and aromatic hydrocarbon degradation: primers and processing matter. Front Microbiol [Internet]. 2013;4. Available from: http://journal.frontiersin.org/article/10.3389/fmicb.2013.00279/abstract

26. Hannula SE, van Veen JA. Primer sets developed for functional genes reveal shifts in functionality of fungal community in soils. Front Microbiol. 2016;7.

27. Barbi F, Bragalini C, Vallon L, Prudent E, Dubost A, Fraissinet-Tachet L, et al. PCR primers to study the diversity of expressed fungal genes encoding lignocellulolytic enzymes in soils using high-throughput sequencing. PLoS One [Internet]. 2014 [cited 2015 Jan 5];9:e116264. Available from: http://journals.plos.org/plosone/article?id=10.1371/journal.pone.0116264#pone-0116264-g004

28. Fish JA, Chai B, Wang Q, Sun Y, Brown CT, Tiedje JM, et al. FunGene: the functional gene pipeline and repository. Front Microbiol [Internet]. 2013;4. Available from: http://journal.frontiersin.org/article/10.3389/fmicb.2013.00291/abstract

29. Angel R, Nepel M, Panhölzl C, Schmidt H, Herbold CW, Eichorst SA, et al. Evaluation of primers targeting the diazotroph functional gene and development of NifMAP - A bioinformatics pipeline for analyzing nifH amplicon data. Front Microbiol [Internet]. 2018;9. Available from: http://journal.frontiersin.org/article/10.3389/fmicb.2018.00703/full

30. Ortiz-Estrada ÁM, Gollas-Galván T, Martínez-Córdova LR, Martínez-Porchas M. Predictive functional profiles using metagenomic 16S rRNA data: a novel approach to understanding the microbial ecology of aquaculture systems. Rev Aquac [Internet]. 2019;11:234–45. Available from: http://doi.wiley.com/10.1111/raq.12237

31. Bahram M, Hildebrand F, Forslund SK, Anderson JL, Soudzilovskaia NA, Bodegom PM, et al. Structure and function of the global topsoil microbiome. Nature [Internet]. 2018;560:233–7. Available from: http://www.nature.com/articles/s41586-018-0386-6

32. Hahn AS, Konwar KM, Louca S, Hanson NW, Hallam SJ. The information science of microbial ecology. Curr Opin Microbiol [Internet]. 2016;31:209–16. Available from: https://linkinghub.elsevier.com/retrieve/pii/S1369527416300492

33. Farley SS, Dawson A, Goring SJ, Williams JW. Situating ecology as a big-data science: Current advances, challenges, and solutions. Bioscience. 2018;68:563–76.

34. Caspi R, Billington R, Keseler IM, Kothari A, Krummenacker M, Midford PE, et al. The MetaCyc database of metabolic pathways and enzymes - a 2019 update. Nucleic Acids Res [Internet]. 2020;48:D445–53. Available from: https://academic.oup.com/nar/article/48/D1/D445/5581728

35. Kanehisa M, Sato Y, Kawashima M, Furumichi M, Tanabe M. KEGG as a reference resource for gene and protein annotation. Nucleic Acids Res. 2016;44:D457–62.

36. Lombard V, Golaconda Ramulu H, Drula E, Coutinho PM, Henrissat B. The carbohydrate-active enzymes database (CAZy) in 2013. Nucleic Acids Res [Internet]. 2014 [cited 2014 Jul 10];42:D490-5. Available from: http://www.pubmedcentral.nih.gov/articlerender.fcgi?artid=3965031&tool=pmcentrez&rendertype=abstract

37. Starke R, Capek P, Morais D, Callister SJ, Jehmlich N. The total microbiome functions in bacteria and fungi. J Proteomics [Internet]. 2020;213:103623. Available from: https://linkinghub.elsevier.com/retrieve/pii/S1874391919303951

38. Commichaux S, Shah N, Ghurye J, Stoppel A, Goodheart JA, Luque GG, et al. A critical assessment of gene catalogs for metagenomic analysis. Birol I, editor. Bioinformatics [Internet]. 2021; Available from: https://academic.oup.com/bioinformatics/advance-article/doi/10.1093/bioinformatics/btab216/6207961

39. Zhou Y, Coventry DR, Gupta VVSR, Fuentes D, Merchant A, Kaiser BN, et al. The preceding root system drives the composition and function of the rhizosphere microbiome. Genome Biol [Internet]. 2020;21:89. Available from: https://genomebiology.biomedcentral.com/articles/10.1186/s13059-020-01999-0

40. Baldrian P. The known and the unknown in soil microbial ecology. FEMS Microbiol Ecol [Internet]. 2019;95. Available from: https://academic.oup.com/femsec/article/doi/10.1093/femsec/fiz005/5281230

41. Blondel J. Guilds or functional groups: Does it matter? Oikos [Internet]. 2003;100:223–31. Available from: http://doi.wiley.com/10.1034/j.1600-0706.2003.12152.x

42. Mlambo MC. Not all traits are “functional”: Insights from taxonomy and biodiversity-ecosystem functioning research. Biodivers Conserv [Internet]. 2014;23:781–90. Available from: http://link.springer.com/10.1007/s10531-014-0618-5

43. Volaire F, Gleason SM, Delzon S. What do you mean “functional” in ecology? Patterns versus processes. Ecol Evol [Internet]. 2020;10:11875–85. Available from: https://onlinelibrary.wiley.com/doi/10.1002/ece3.6781

44. Escalas A, Hale L, Voordeckers JW, Yang Y, Firestone MK, Alvarez-Cohen L, et

al. Microbial functional diversity: From concepts to applications. Ecol Evol [Internet]. 2019;9:12000–16. Available from: https://onlinelibrary.wiley.com/doi/abs/10.1002/ece3.5670

45. Malik AA, Martiny JBH, Brodie EL, Martiny AC, Treseder KK, Allison SD. Defining trait-based microbial strategies with consequences for soil carbon cycling under climate change. ISME J [Internet]. 2020;14:1–9. Available from: http://www.nature.com/articles/s41396-019-0510-0

46. Madin JS, Nielsen DA, Brbic M, Corkrey R, Danko D, Edwards K, et al. A synthesis of bacterial and archaeal phenotypic trait data. Sci Data [Internet]. 2020;7:170. Available from: http://www.nature.com/articles/s41597-020-0497-4

47. Lajoie G, Kembel SW. Making the Most of Trait-Based Approaches for Microbial Ecology. Trends Microbiol [Internet]. 2019;27:814–23. Available from: https://linkinghub.elsevier.com/retrieve/pii/S0966842X19301581

48. Reimer LC, Söhngen C, Vetcininova A, Overmann J. Mobilization and integration of bacterial phenotypic data—Enabling next generation biodiversity analysis through the Bac Dive metadatabase. J Biotechnol [Internet]. 2017;261:187–93. Available from: https://linkinghub.elsevier.com/retrieve/pii/S0168165617302067

49. Endara L, Cui H, Burleigh JG. Extraction of phenotypic traits from taxonomic descriptions for the tree of life using natural language processing. Appl Plant Sci [Internet]. 2018;6:e1035. Available from: http://doi.wiley.com/10.1002/aps3.1035

50. Lim KMK, Li C, Chng KR, Nagarajan N. @MInter: Automated text-mining of microbial interactions. Bioinformatics [Internet]. 2016;32:2981–7. Available from: https://academic.oup.com/bioinformatics/article-lookup/doi/10.1093/bioinformatics/btw357

51. Chaix E, Deléger L, Bossy R, Nédellec C. Text mining tools for extracting

information about microbial biodiversity in food. Food Microbiol [Internet]. 2019;81:63–75. Available from: https://linkinghub.elsevier.com/retrieve/pii/S0740002017310638

52. Chibucos MC, Zweifel AE, Herrera JC, Meza W, Eslamfam S, Uetz P, et al. An ontology for microbial phenotypes. BMC Microbiol [Internet]. 2014;14:294. Available from: http://bmcmicrobiol.biomedcentral.com/articles/10.1186/s12866-014-0294-3

53. Brbić M, Piškorec M, Vidulin V, Kriško A, Šmuc T, Supek F. Phenotype Inference from Text and Genomic Data. Lect Notes Comput Sci (including Subser Lect Notes Artif Intell Lect Notes Bioinformatics) [Internet]. 2017. p. 373–7. Available from: http://link.springer.com/10.1007/978-3-319-71273-4_34

54. Langille MG, Zaneveld J, Caporaso JG, McDonald D, Knights D, Reyes JA, et al. Predictive functional profiling of microbial communities using 16S rRNA marker gene sequences. Nat Biotechnol [Internet]. 2013;31:814–21. Available from: http://www.ncbi.nlm.nih.gov/pubmed/23975157

55. Nguyen NH, Song Z, Bates ST, Branco S, Tedersoo L, Menke J, et al. FUNGuild: An open annotation tool for parsing fungal community datasets by ecological guild. Fungal Ecol [Internet]. Elsevier Ltd; 2016;20:241–8. Available from: http://dx.doi.org/10.1016/j.funeco.2015.06.006

56. Aßhauer KP, Wemheuer B, Daniel R, Meinicke P. Tax4Fun: Predicting functional profiles from metagenomic 16S rRNA data. Bioinformatics [Internet]. 2015 [cited 2015 May 13];31:2882–4. Available from: http://www.pubmedcentral.nih.gov/articlerender.fcgi?artid=4547618&tool=pmcentrez&rendertype=abstract

57. Louca S, Parfrey LW, Doebeli M. Decoupling function and taxonomy in the global ocean microbiome. Science (80-) [Internet]. 2016;353:1272–7. Available from:

https://www.sciencemag.org/lookup/doi/10.1126/science.aaf4507

58. Segata N, Huttenhower C. Toward an Efficient Method of Identifying Core Genes for Evolutionary and Functional Microbial Phylogenies. Gibas C, editor. PLoS One [Internet]. 2011;6:e24704. Available from: https://dx.plos.org/10.1371/journal.pone.0024704

59. Snel B, Bork P, Huynen MA. Genome phylogeny based on gene content. Nat Genet [Internet]. 1999;21:108–10. Available from: http://www.nature.com/articles/ng0199_108

60. Hartman WH, Ye R, Horwath WR, Tringe SG. A genomic perspective on stoichiometric regulation of soil carbon cycling. ISME J [Internet]. 2017;11:2652–65. Available from: http://www.nature.com/articles/ismej2017115

61. Kembel SW, Wu M, Eisen JA, Green JL. Incorporating 16S Gene Copy Number Information Improves Estimates of Microbial Diversity and Abundance. von Mering C, editor. PLoS Comput Biol [Internet]. 2012;8:e1002743. Available from: https://dx.plos.org/10.1371/journal.pcbi.1002743

62. Louca S, Doebeli M, Parfrey LW. Correcting for 16S rRNA gene copy numbers in microbiome surveys remains an unsolved problem. Microbiome [Internet]. 2018;6:41. Available from: http://www.embase.com/search/results?subaction=viewrecord&from=export&id=L620952556%0Ahttp://dx.doi.org/10.1186/s40168-018-0420-9

63. Woloszynek S, Mell JC, Zhao Z, Simpson G, O’Connor MP, Rosen GL. Exploring thematic structure and predicted functionality of 16S rRNA amplicon data. Loor JJ, editor. PLoS One [Internet]. 2019;14:e0219235. Available from: https://dx.plos.org/10.1371/journal.pone.0219235

64. Dhariwal A, Chong J, Habib S, King IL, Agellon LB, Xia J. MicrobiomeAnalyst: a

web-based tool for comprehensive statistical, visual and meta-analysis of microbiome data. Nucleic Acids Res [Internet]. 2017;45:W180–8. Available from: https://academic.oup.com/nar/article-lookup/doi/10.1093/nar/gkx295

65. Douglas GM, Maffei VJ, Zaneveld JR, Yurgel SN, Brown JR, Taylor CM, et al. PICRUSt2 for prediction of metagenome functions. Nat Biotechnol [Internet]. 2020;38:685–8. Available from: http://www.nature.com/articles/s41587-020-0548-6

66. Bowman JS, Ducklow HW. Microbial communities can be described by metabolic structure: A general framework and application to a seasonally variable, depth-stratified microbial community from the coastal West Antarctic Peninsula. PLoS One. 2015;10:1–18.

67. R Core Team. R: A Language and Environment for Statistical Computing [Internet]. Vienna, Austria; 2020. Available from: https://www.r-project.org/

68. Iwai S, Weinmaier T, Schmidt BL, Albertson DG, Poloso NJ, Dabbagh K, et al. Piphillin: Improved Prediction of Metagenomic Content by Direct Inference from Human Microbiomes. He Z, editor. PLoS One [Internet]. 2016;11:e0166104. Available from: https://dx.plos.org/10.1371/journal.pone.0166104

69. Mitchell K, Ronas J, Dao C, Freise AC, Mangul S, Shapiro C, et al. PUMAA: A Platform for Accessible Microbiome Analysis in the Undergraduate Classroom. Front Microbiol [Internet]. 2020;11. Available from: https://www.frontiersin.org/article/10.3389/fmicb.2020.584699/full

70. Zanne AE, Abarenkov K, Afkhami ME, Aguilar-Trigueros CA, Bates S, Bhatnagar JM, et al. Fungal functional ecology: bringing a trait-based approach to plant-associated fungi. Biol Rev [Internet]. 2020;95:409–33. Available from: https://onlinelibrary.wiley.com/doi/abs/10.1111/brv.12570

71. Põlme S, Abarenkov K, Henrik Nilsson R, Lindahl BD, Clemmensen KE,

Kauserud H, et al. FungalTraits: a user-friendly traits database of fungi and fungus-like stramenopiles. Fungal Divers [Internet]. 2020;105:1–16. Available from: http://link.springer.com/10.1007/s13225-020-00466-2

72. Fones HN, Bebber DP, Chaloner TM, Kay WT, Steinberg G, Gurr SJ. Threats to global food security from emerging fungal and oomycete crop pathogens. Nat Food [Internet]. 2020;1:332–42. Available from: http://www.nature.com/articles/s43016-020-0075-0

73. Agerer R, Rambold G. DEEMY—an information system for characterization and determination of ectomycorrhizae. München, Ger [Internet]. 2004; Available from: http://www.deemy.de/

74. Barberán A, Caceres Velazquez H, Jones S, Fierer N. Hiding in Plain Sight: Mining Bacterial Species Records for Phenotypic Trait Information. Hallam SJ, editor. mSphere [Internet]. 2017;2. Available from: https://msphere.asm.org/content/2/4/e00237-17

75. Reimer LC, Vetcininova A, Carbasse JS, Söhngen C, Gleim D, Ebeling C, et al. Bac Dive in 2019: bacterial phenotypic data for High-throughput biodiversity analysis. Nucleic Acids Res [Internet]. 2019;47:D631–6. Available from: https://academic.oup.com/nar/article/47/D1/D631/5106998

76. Engqvist MKM. Correlating enzyme annotations with a large set of microbial growth temperatures reveals metabolic adaptations to growth at diverse temperatures. BMC Microbiol [Internet]. 2018;18:177. Available from: https://bmcmicrobiol.biomedcentral.com/articles/10.1186/s12866-018-1320-7

77. Nkongolo KK, Narendrula-Kotha R. Advances in monitoring soil microbial community dynamic and function. J Appl Genet [Internet]. 2020;61:249–63. Available from: http://link.springer.com/10.1007/s13353-020-00549-5

78. Jin T, Wang Y, Huang Y, Xu J, Zhang P, Wang N, et al. Taxonomic structure and functional association of foxtail millet root microbiome. Gigascience. 2017;6:1–12.

79. Lian T, Mu Y, Jin J, Ma Q, Cheng Y, Cai Z, et al. Impact of intercropping on the coupling between soil microbial community structure, activity, and nutrient-use efficiencies. PeerJ [Internet]. 2019;7:e6412. Available from: https://peerj.com/articles/6412

80. Sengupta A, Hariharan J, Grewal PS, Dick WA. Bacterial community dissimilarity in soils is driven by long-term land-use practices. Agrosystems, Geosci Environ. 2020;3.

81. Lüneberg K, Schneider D, Siebe C, Daniel R. Drylands soil bacterial community is affected by land use change and different irrigation practices in the Mezquital Valley, Mexico. Sci Rep [Internet]. 2018;8:1413. Available from: http://www.nature.com/articles/s41598-018-19743-x

82. Sun S, Jones RB, Fodor AA. Inference-based accuracy of metagenome prediction tools varies across sample types and functional categories. Microbiome [Internet]. 2020;8:46. Available from: https://microbiomejournal.biomedcentral.com/articles/10.1186/s40168-020-00815-y

83. Koo H, Hakim JA, Morrow CD, Eipers PG, Davila A, Andersen DT, et al. Comparison of two bioinformatics tools used to characterize the microbial diversity and predictive functional attributes of microbial mats from Lake Obersee, Antarctica. J Microbiol Methods [Internet]. 2017;140:15–22. Available from: https://linkinghub.elsevier.com/retrieve/pii/S0167701217301781

84. Martiny AC, Treseder K, Pusch G. Phylogenetic conservatism of functional traits in microorganisms. ISME J [Internet]. 2013;7:830–8. Available from: http://www.nature.com/articles/ismej2012160

85. George PBL, Creer S, Griffiths RI, Emmett BA, Robinson DA, Jones DL. Primer and Database Choice Affect Fungal Functional but Not Biological Diversity Findings in a National Soil Survey. Front Environ Sci [Internet]. 2019;7. Available from: https://www.frontiersin.org/article/10.3389/fenvs.2019.00173/full

86. Yang T, Adams JM, Shi Y, He JS, Jing X, Chen L, et al. Soil fungal diversity in natural grasslands of the Tibetan Plateau: associations with plant diversity and productivity. New Phytol [Internet]. 2017;215:756–65. Available from: https://onlinelibrary.wiley.com/doi/10.1111/nph.14606

87. Makiola A, Dickie IA, Holdaway RJ, Wood JR, Orwin KH, Glare TR. Land use is a determinant of plant pathogen alpha- but not beta-diversity. Mol Ecol [Internet]. 2019;28:3786–98. Available from: https://onlinelibrary.wiley.com/doi/abs/10.1111/mec.15177

88. Buscardo E, Souza RC, Meir P, Geml J, Schmidt SK, da Costa ACL, et al. Effects of natural and experimental drought on soil fungi and biogeochemistry in an Amazon rain forest. Commun Earth Environ [Internet]. 2021;2:55. Available from: http://www.nature.com/articles/s43247-021-00124-8

89. Liang M, Liu X, Parker IM, Johnson D, Zheng Y, Luo S, et al. Soil microbes drive phylogenetic diversity-productivity relationships in a subtropical forest. Sci Adv [Internet]. 2019;5:eaax5088. Available from: https://advances.sciencemag.org/lookup/doi/10.1126/sciadv.aax5088

90. Yang T, Tedersoo L, Soltis PS, Soltis DE, Gilbert JA, Sun M, et al. Phylogenetic imprint of woody plants on the soil mycobiome in natural mountain forests of eastern China. ISME J [Internet]. 2019;13:686–97. Available from: http://www.nature.com/articles/s41396-018-0303-x

91. Brinkmann N, Schneider D, Sahner J, Ballauff J, Edy N, Barus H, et al. Intensive

tropical land use massively shifts soil fungal communities. Sci Rep [Internet]. 2019;9:3403. Available from: http://www.nature.com/articles/s41598-019-39829-4

92. Egidi E, Delgado-Baquerizo M, Plett JM, Wang J, Eldridge DJ, Bardgett RD, et al. A few Ascomycota taxa dominate soil fungal communities worldwide. Nat Commun [Internet]. 2019;10:2369. Available from: http://www.nature.com/articles/s41467-019-10373-z

93. Delgado-Baquerizo M, Guerra CA, Cano-Díaz C, Egidi E, Wang J-T, Eisenhauer N, et al. The proportion of soil-borne pathogens increases with warming at the global scale. Nat Clim Chang [Internet]. 2020;10:550–4. Available from: http://www.nature.com/articles/s41558-020-0759-3

94. Větrovský T, Kohout P, Kopecký M, Machac A, Man M, Bahnmann BD, et al. A meta-analysis of global fungal distribution reveals climate-driven patterns. Nat Commun [Internet]. 2019;10:5142. Available from: http://www.nature.com/articles/s41467-019-13164-8

95. Öpik M, Davison J, Moora M, Zobel M. DNA-based detection and identification of Glomeromycota: the virtual taxonomy of environmental sequences. Botany [Internet]. 2014;92:135–47. Available from: http://www.nrcresearchpress.com/doi/10.1139/cjb-2013-0110

96. Berruti A, Desirò A, Visentin S, Zecca O, Bonfante P. ITS fungal barcoding primers versus 18S AMF-specific primers reveal similar AMF-based diversity patterns in roots and soils of three mountain vineyards. Environ Microbiol Rep [Internet]. 2017;9:658–67. Available from: http://doi.wiley.com/10.1111/1758-2229.12574

97. Anthony MA, Frey SD, Stinson KA. Fungal community homogenization, shift in dominant trophic guild, and appearance of novel taxa with biotic invasion. Ecosphere [Internet]. 2017;8:e01951. Available from: http://doi.wiley.com/10.1002/ecs2.1951

98. Sansupa C, Wahdan SFM, Hossen S, Disayathanoowat T, Wubet T, Purahong W. Can we use functional annotation of prokaryotic taxa (Faprotax) to assign the ecological functions of soil bacteria? Appl Sci [Internet]. 2021;11:1–17. Available from: https://www.mdpi.com/2076-3417/11/2/688

99. Nilsson RH, Anslan S, Bahram M, Wurzbacher C, Baldrian P, Tedersoo L. Mycobiome diversity: high-throughput sequencing and identification of fungi. Nat Rev Microbiol [Internet]. 2019;17:95–109. Available from: http://www.nature.com/articles/s41579-018-0116-y

100. Comeau AM, Douglas GM, Langille MGI. Microbiome Helper: a Custom and Streamlined Workflow for Microbiome Research. Eisen J, editor. mSystems [Internet]. 2017;2. Available from: https://msystems.asm.org/content/2/1/e00127-16

101. Piper AM, Batovska J, Cogan NOI, Weiss J, Cunningham JP, Rodoni BC, et al. Prospects and challenges of implementing DNA metabarcoding for high-throughput insect surveillance. Gigascience [Internet]. 2019;8:1–22. Available from: https://academic.oup.com/gigascience/article/doi/10.1093/gigascience/giz092/5541630

102. Gogarten JP, Townsend JP. Horizontal gene transfer, genome innovation and evolution. Nat Rev Microbiol [Internet]. 2005;3:679–87. Available from: http://www.nature.com/articles/nrmicro1204

103. Douglas GM, Langille MGI. Current and Promising Approaches to Identify Horizontal Gene Transfer Events in Metagenomes. Dagan T, editor. Genome Biol Evol [Internet]. 2019;11:2750–66. Available from: https://academic.oup.com/gbe/article/11/10/2750/5554466

104. Seiler E, Trappe K, Renard BY. Where did you come from, where did you go: Refining metagenomic analysis tools for horizontal gene transfer characterisation.

Dessimoz C, editor. PLoS Comput Biol [Internet]. 2019;15:e1007208. Available from: https://dx.plos.org/10.1371/journal.pcbi.1007208

105. van Dijk B, Hogeweg P, Doekes HM, Takeuchi N. Slightly beneficial genes are retained by bacteria evolving DNA uptake despite selfish elements. Elife [Internet]. 2020;9:1–36. Available from: https://elifesciences.org/articles/56801

106. Treseder KK, Lennon JT. Fungal Traits That Drive Ecosystem Dynamics on Land. Microbiol Mol Biol Rev [Internet]. 2015;79:243–62. Available from: http://mmbr.asm.org/lookup/doi/10.1128/MMBR.00001-15

107. Smalla K, Jechalke S, Top EM. Plasmid Detection, Characterization, and Ecology. Microbiol Spectr [Internet]. 2015;3. Available from: http://www.asmscience.org/content/journal/microbiolspec/10.1128/microbiolspec.PLAS-0038-2014

108. Dunivin TK, Choi J, Howe A, Shade A. RefSoil+: a Reference Database for Genes and Traits of Soil Plasmids. Sharpton TJ, editor. mSystems [Internet]. 2019;4. Available from: https://msystems.asm.org/content/4/1/e00349-18

109. Aminov RI. Horizontal Gene Exchange in Environmental Microbiota. Front Microbiol [Internet]. 2011;2. Available from: http://journal.frontiersin.org/article/10.3389/fmicb.2011.00158/abstract

110. Brito IL. Examining horizontal gene transfer in microbial communities. Nat Rev Microbiol [Internet]. 2021;19:442–53. Available from: http://www.nature.com/articles/s41579-021-00534-7

111. Banos S, Lentendu G, Kopf A, Wubet T, Glöckner FO, Reich M. A comprehensive fungi-specific 18S rRNA gene sequence primer toolkit suited for diverse research issues and sequencing platforms. BMC Microbiol [Internet]. 2018;18:190. Available from:

https://bmcmicrobiol.biomedcentral.com/articles/10.1186/s12866-018-1331-4

112. Bukin YS, Galachyants YP, Morozov I V., Bukin S V., Zakharenko AS, Zemskaya TI. The effect of 16S rRNA region choice on bacterial community metabarcoding results. Sci Data [Internet]. 2019;6:190007. Available from: http://www.nature.com/articles/sdata20197

113. Xu Z, Malmer D, Langille MGI, Way SF, Knight R. Which is more important for classifying microbial communities: who’s there or what they can do? ISME J [Internet]. International Society for Microbial Ecology; 2014 [cited 2015 Aug 31];8:2357–9. Available from: http://dx.doi.org/10.1038/ismej.2014.157

114. Karst SM, Dueholm MS, McIlroy SJ, Kirkegaard RH, Nielsen PH, Albertsen M. Retrieval of a million high-quality, full-length microbial 16S and 18S rRNA gene sequences without primer bias. Nat Biotechnol [Internet]. Nature Publishing Group; 2018;36:190–5. Available from: http://dx.doi.org/10.1038/nbt.4045

115. Tedersoo L, Anslan S. Towards PacBio-based pan-eukaryote metabarcoding using full-length ITS sequences. Environ Microbiol Rep [Internet]. 2019;11:659–68. Available from: https://onlinelibrary.wiley.com/doi/abs/10.1111/1758-2229.12776

116. Johnson JS, Spakowicz DJ, Hong B-Y, Petersen LM, Demkowicz P, Chen L, et al. Evaluation of 16S rRNA gene sequencing for species and strain-level microbiome analysis. Nat Commun [Internet]. 2019;10:5029. Available from: http://www.nature.com/articles/s41467-019-13036-1

117. Philippot L, Andersson SGE, Battin TJ, Prosser JI, Schimel JP, Whitman WB, et al. The ecological coherence of high bacterial taxonomic ranks. Nat Rev Microbiol [Internet]. 2010;8:523–9. Available from: http://www.nature.com/articles/nrmicro2367

118. Fierer N, Bradford MA, Jackson RB. TOWARD AN ECOLOGICAL CLASSIFICATION OF SOIL BACTERIA. Ecology [Internet]. 2007;88:1354–64.

Available from: http://doi.wiley.com/10.1890/05-1839

119. Callahan BJ, Wong J, Heiner C, Oh S, Theriot CM, Gulati AS, et al. High-throughput amplicon sequencing of the full-length 16S rRNA gene with single-nucleotide resolution. Nucleic Acids Res [Internet]. 2019;47:e103–e103. Available from: https://academic.oup.com/nar/article/47/18/e103/5527971

120. Feldbauer R, Schulz F, Horn M, Rattei T. Prediction of microbial phenotypes based on comparative genomics. BMC Bioinformatics [Internet]. 2015;16:S1. Available from: http://bmcbioinformatics.biomedcentral.com/articles/10.1186/1471-2105-16-S14-S1

121. Weimann A, Mooren K, Frank J, Pope PB, Bremges A, McHardy AC. From Genomes to Phenotypes: Traitar, the Microbial Trait Analyzer. Segata N, editor. mSystems [Internet]. 2016;1:043315. Available from: https://msystems.asm.org/content/1/6/e00101-16

122. Goberna M, Verdú M. Predicting microbial traits with phylogenies. ISME J [Internet]. 2016;10:959–67. Available from: http://www.nature.com/articles/ismej2015171

123. Levatić J, Brbić M, Perdih TS, Kocev D, Vidulin V, Šmuc T, et al. Phenotype Prediction with Semi-supervised Classification Trees. Lect Notes Comput Sci (including Subser Lect Notes Artif Intell Lect Notes Bioinformatics) [Internet]. 2018. p. 138–50. Available from: http://link.springer.com/10.1007/978-3-319-78680-3_10

124. Zanne AE, Powell JR, Flores-Moreno H, Kiers ET, van ’t Padje A, Cornwell WK. Finding fungal ecological strategies: Is recycling an option? Fungal Ecol [Internet]. 2020;46:100902. Available from: https://linkinghub.elsevier.com/retrieve/pii/S1754504819302181

125. Kembel SW, Cowan PD, Helmus MR, Cornwell WK, Morlon H, Ackerly DD, et

al. Picante: R tools for integrating phylogenies and ecology. Bioinformatics [Internet]. 2010;26:1463–4. Available from: http://www.ncbi.nlm.nih.gov/sites/entrez?Db=pubmed&DbFrom=pubmed&Cmd=Link &LinkName=pubmed_pubmed&LinkReadableName=Related Articles&IdsFromResult=20395285&ordinalpos=3&itool=EntrezSystem2.PEntrez.Pub med.Pubmed_ResultsPanel.Pubmed_RVDocSum http://www.ncbi.n

126. Louca S, Doebeli M. Efficient comparative phylogenetics on large trees. Valencia A, editor. Bioinformatics [Internet]. 2018;34:1053–5. Available from: https://academic.oup.com/bioinformatics/article/34/6/1053/4582279

127. Walters KE, Martiny JBH. Alpha-, beta-, and gamma-diversity of bacteria varies across habitats. Nabout JC, editor. PLoS One [Internet]. 2020;15:e0233872. Available from: https://dx.plos.org/10.1371/journal.pone.0233872

128. Martiny AC. High proportions of bacteria are culturable across major biomes. ISME J [Internet]. Springer US; 2019;3–6. Available from: http://dx.doi.org/10.1038/s41396-019-0410-3

129. Wemheuer F, Taylor JA, Daniel R, Johnston E, Meinicke P, Thomas T, et al. Tax4Fun2: prediction of habitat-specific functional profiles and functional redundancy based on 16S rRNA gene sequences. Environ Microbiome [Internet]. 2020;15:11. Available from: https://environmentalmicrobiome.biomedcentral.com/articles/10.1186/s40793-020-00358-7

130. Cheifet B. Where is genomics going next? Genome Biol [Internet]. 2019;20:17. Available from: https://genomebiology.biomedcentral.com/articles/10.1186/s13059-019-1626-2

131. Piro VC, Dadi TH, Seiler E, Reinert K, Renard BY. ganon: precise

metagenomics classification against large and up-to-date sets of reference sequences. Bioinformatics [Internet]. 2020;36:i12–20. Available from: https://academic.oup.com/bioinformatics/article/36/Supplement_1/i12/5870470

132. Choi J, Yang F, Stepanauskas R, Cardenas E, Garoutte A, Williams R, et al. Strategies to improve reference databases for soil microbiomes. ISME J [Internet]. 2017;11:829–34. Available from: http://www.nature.com/articles/ismej2016168

133. Louca S, Jacques SMS, Pires APF, Leal JS, Srivastava DS, Parfrey LW, et al. High taxonomic variability despite stable functional structure across microbial communities. Nat Ecol Evol [Internet]. 2017;1:0015. Available from: http://www.nature.com/articles/s41559-016-0015

134. Nagpal S, Haque MM, Singh R, Mande SS. iVikodak—A Platform and Standard Workflow for Inferring, Analyzing, Comparing, and Visualizing the Functional Potential of Microbial Communities. Front Microbiol [Internet]. 2019;9. Available from: https://www.frontiersin.org/article/10.3389/fmicb.2018.03336/full

135. Grigoriev I V., Nikitin R, Haridas S, Kuo A, Ohm R, Otillar R, et al. MycoCosm portal: Gearing up for 1000 fungal genomes. Nucleic Acids Res. 2014;42:699–704.

136. Bonkowski M, Dumack K, Fiore-Donno AM. The Protists in Soil—A Token of Untold Eukaryotic Diversity. Mod Soil Microbiol [Internet]. Third edition. | Boca Raton : Taylor & Francis, 2019.: CRC Press; 2019. p. 125–40. Available from: https://www.taylorfrancis.com/books/9780429607929/chapters/10.1201/9780429059186-8

137. Xiong W, Jousset A, Guo S, Karlsson I, Zhao Q, Wu H, et al. Soil protist communities form a dynamic hub in the soil microbiome. ISME J [Internet]. 2018;12:634–8. Available from: http://www.nature.com/articles/ismej2017171

138. Fiore-Donno AM, Richter-Heitmann T, Degrune F, Dumack K, Regan KM,

Marhan S, et al. Functional Traits and Spatio-Temporal Structure of a Major Group of Soil Protists (Rhizaria: Cercozoa) in a Temperate Grassland. Front Microbiol [Internet]. 2019;10. Available from: https://www.frontiersin.org/article/10.3389/fmicb.2019.01332/full

139. Delgado-Baquerizo M, Trivedi P, Trivedi C, Eldridge DJ, Reich PB, Jeffries TC, et al. Microbial richness and composition independently drive soil multifunctionality. Bennett A, editor. Funct Ecol [Internet]. 2017;31:2330–43. Available from: https://onlinelibrary.wiley.com/doi/abs/10.1111/1365-2435.12924

140. Terrat S, Horrigue W, Dequietd S, Saby NPA, Lelièvre M, Nowak V, et al. Mapping and predictive variations of soil bacterial richness across France. PLoS One. 2017;12:5–8.

141. Schloter M, Nannipieri P, Sørensen SJ, van Elsas JD. Microbial indicators for soil quality. Biol Fertil Soils. Biology and Fertility of Soils; 2018;54:1–10.

142. Hariharan J, Sengupta A, Grewal P, Dick WA. Functional Predictions of Microbial Communities in Soil as Affected by Long-term Tillage Practices. Agric Environ Lett [Internet]. 2017;2:170031. Available from: https://onlinelibrary.wiley.com/doi/10.2134/ael2017.09.0031

143. Manor O, Borenstein E. Systematic Characterization and Analysis of the Taxonomic Drivers of Functional Shifts in the Human Microbiome. Cell Host Microbe [Internet]. 2017;21:254–67. Available from: https://linkinghub.elsevier.com/retrieve/pii/S1931312816305261

144. Zhang Q, Zhao H, Wu D, Cao D, Ma W. A comprehensive analysis of the microbiota composition and gene expression in colorectal cancer. BMC Microbiol [Internet]. 2020;20:308. Available from: https://bmcmicrobiol.biomedcentral.com/articles/10.1186/s12866-020-01938-w

145. Zhang L, Liu Y, Zheng HJ, Zhang CP. The Oral Microbiota May Have Influence on Oral Cancer. Front Cell Infect Microbiol [Internet]. 2020;9. Available from: https://www.frontiersin.org/article/10.3389/fcimb.2019.00476/full

146. Ji P, Rhoads WJ, Edwards MA, Pruden A. Impact of water heater temperature setting and water use frequency on the building plumbing microbiome. ISME J [Internet]. 2017;11:1318–30. Available from: http://www.nature.com/articles/ismej201714

147. Maguvu TE, Bezuidenhout CC, Kritzinger R, Tsholo K, Plaatjie M, Molale-Tom LG, et al. Combining physicochemical properties and microbiome data to evaluate the water quality of South African drinking water production plants. Aziz RK, editor. PLoS One [Internet]. 2020;15:e0237335. Available from: https://dx.plos.org/10.1371/journal.pone.0237335

148. Ramirez KS, Döring M, Eisenhauer N, Gardi C, Ladau J, Leff JW, et al. Toward a global platform for linking soil biodiversity data. Front Ecol Evol [Internet]. 2015;3. Available from: http://journal.frontiersin.org/Article/10.3389/fevo.2015.00091/abstract

149. Raguideau S, Plancade S, Pons N, Leclerc M, Laroche B. Inferring Aggregated Functional Traits from Metagenomic Data Using Constrained Non-negative Matrix Factorization: Application to Fiber Degradation in the Human Gut Microbiota. Maranas CD, editor. PLOS Comput Biol [Internet]. 2016;12:e1005252. Available from: https://dx.plos.org/10.1371/journal.pcbi.1005252

150. Fierer N, Barberán A, Laughlin DC. Seeing the forest for the genes: Using metagenomics to infer the aggregated traits of microbial communities. Front Microbiol [Internet]. 2014;5. Available from: http://journal.frontiersin.org/article/10.3389/fmicb.2014.00614/abstract

151. Weisskopf L, Schulz S, Garbeva P. Microbial volatile organic compounds in

intra-kingdom and inter-kingdom interactions. Nat Rev Microbiol [Internet]. 2021;19:391–404. Available from: http://www.nature.com/articles/s41579-020-00508-1

152. Allison SD, Martiny JBH. Resistance, resilience, and redundancy in microbial communities. Proc Natl Acad Sci [Internet]. 2008;105:11512–9. Available from: http://www.pnas.org/cgi/doi/10.1073/pnas.0801925105

153. Navarro LM, Fernández N, Guerra C, Guralnick R, Kissling WD, Londoño MC, et al. Monitoring biodiversity change through effective global coordination. Curr Opin Environ Sustain [Internet]. 2017;29:158–69. Available from: https://linkinghub.elsevier.com/retrieve/pii/S1877343517301665

Tables

Table 1: **Numbers of organisms, genes, enzymes and metabolic pathways available in the CAZy, KEGG and MetaCyc databases.** When possible, we detailed the number of organisms for the three domains of the tree of life. CAZy includes glycoside hydrolases (GH), glycosyl transferases (GT), carbohydrate esterases (CE), polysaccharide lyases (PL), and auxiliary activities (AA).

Databases	Organisms	Metabolic Pathways	Enzymes/Genes
CAZy (Carbohydrate-Active Enzymes)	Eukaryotes (344), Bacteria (20,421), Archaea (413)	NA	GH (171), GT (114), PL (41), CE (19), AA (16)
KEGG (Kyoto Encyclopedia of Genes and Genomes)	Eukaryotes (557), Bacteria (6,317), Archaea (344)	547	KEGG Orthology (KO) groups 24,402
MetaCyc (metabolic path-ways and enzymes)	Total (3,295)	2,937	13,356

Table 2: **List of the functional inference tools, ecological trait assignment tools and databases.**

Tools	Implementation	Targeted genes	Functional Prediction	Approaches	Methods	Inputs used	Strengths and Specificities	Limitations
PanFP	Perl (recently Python)	16S rRNA	KEGG Orthology; Gene Ontology; Pfam; TIGRFAM	Functional inference	builds a pangenome	NCBI taxonomy	- uses functional profile of the pangenome so could be less sensitive to horizontal gene transfer	- evolutionary models are not taken into account<br>- no confidence score generated<br>- not yet available for microbial eukaryotes
PAPRICA	Python	16S/18S rRNA	MetaCyc ontology	Functional inference	phylogenetic placement	based on rDNA amplicon sequences	- 18S rRNA amplicons are taken into account<br>- examples on the developer's blog	- errors may occur with sequence placement due to poor resolution of rRNA amplicons in some clades
PICRUSt	Python	16S rRNA	KEGG Orthology; KEGG Pathway; COG; CAZy*	Functional inference	ASR (Wagner Parsimony, ACE ML, ACE REML, ACE PIC)	Greengenes taxonomy (18may2012 or v13.5/v13.8)	- evolutionary models are taken into account<br>- confidence score generated (NSTI)<br>- correction of OTU copy numbers	- based on specific taxonomy (GreenGenes identifiers)<br>- KEGG database not updated since 2011<br>- no pre-calculated table of fungal genomes available
PICRUSt2	Python / R	16S/18S rRNA/ ITS	MetaCyc; KEGG Orthology; EC number, COGS, Pfam, TIGRFAM	Functional inference	HSP (maximum parcimony, empirical probabilities, subtree averaging, SCP)	based on rDNA amplicon sequences	- evolutionary models are taken into account<br>- confidence score generated (NSTI)<br>- twice as many KO scores<br>- multiple HSP methods can be implemented (takes branch length weighting into account)<br>- 18S rRNA and ITS amplicons are taken into account	- errors may occur with sequence placement due to poor resolution of rRNA amplicons in some clades

							- extensive documentation and active community	
Piphillin	Web-based	16S rRNA	BioCyc; KEGG	Functional inference	Nearest-neighbor matching of 16S rRNA gene amplicons with genomes from reference databases	based on rDNA amplicon sequences	- regular updates of functional databases<br>- rRNA copy number adjustement	- available online only<br>- available for 16S rRNA only
SINAPS	USEARCH	16S rRNA	Trait annotation (e.g., energy metabolism, Gram-positive staining, presence of a flagellum)	Functional inference	word counting	Greengenes; SILVA	- confidence is estimated by boostrapping<br>- integrated to USEARCH tool	- no peer-reviewed publication (biorxiv preprint)<br>- detailed explanation is missing (e.g., how was protrait input created?)
Tax4Fun	R package	16S rRNA	KEGG Orthology	Functional inference	nearest-neighbour search based on a minimum 16S rRNA sequence similarity	SILVA taxonomy	- uses R (multiplatform) with pre-calculated files<br>- confidence score generated (FTU and FSU)<br>- the algorithm could better predict poorly characterized taxa compared to approaches based on ASR with possible large distances in the tree, thanks to a minimum of similarity between sequences	- based on specific taxonomy (SILVA identifiers)<br>- KEGG database not updated since 2011

Tax4Fun2	R package	16S rRNA	KEGG Orthology	Functional inference	BLAST	based on rDNA amplicon sequences	- algorithm with a minimal sequence similarity<br>- uses R (multiplatform) with pre-calculated, highly memory-efficient platform-independent files<br>- confidence score generated (FTU and FSU)<br>- KO update from 2018<br>- calculates the redundancy of specific functions directly<br>- builds its own habitat-specific reference	- not yet available for microbial eukaryotes
Vikodak	Web-based (not longer available)	16S rRNA	KEGG pathway, EC number	Functional inference	microbial co-existence patterns	RDP, SILVA	- pathway exclusion cut-off value is available to provide the minimum percentage of genes/enzymes belonging to a metabolic pathway required to consider the pathway as functional.<br>- compares two datasets	- not longer available<br>- not yet available for microbial eukaryotes
iVikodak	Web-based	16S rRNA	KEGG; Pfam; COG; TIGRfam	Functional inference	microbial co-inhabitance patterns	RDP, Greengenes, SILVA	- user-friendly for non-expert bioinformaticians<br>- integrated tools for statistical comparisons<br>- graphical visualizations	- available online only<br>- not yet available for microbial eukaryotes
FUNGuild	Python / Web-based	ITS	Guild type	Trait assignment	not applicable	based on UNITE taxonomy (ITS)	- trait quality for taxon assignment	- no regular update<br>- 18S rRNA taxonomy with related database not included. However, the database is open-access, and a homemade wrapper can be used for 18S metabarcoding output

FAPRO-TAX	Python; flat file	16S rRNA	Ecological functions (e.g., nitrification, denitrification or fermentation)	Trait assignment; Database	If all type strains of a species at the genus level share the function, FAPROTAX assumes that all uncultured organisms of this genus possess the putative function	SILVA (128, 132)	- based on the literature of cultured taxa<br>- availability of all literature to create the database<br>- functions easily added to the tool	- implicit assumption (see algorithm column) could be false with the increase of newly cultured organisms<br>- does not infer upper rank when taxonomic resolution is poor
BacDive	Python and R API, R package	/	Morphology, physiology (API®-tests), molecular data, and cultivation conditions	Database	not applicable	NCBI taxonomy	- provides links to ENA, GenBank, SILVA, BRENDA, GBIF, ChEBI, Straininfo website data<br>- a match with 16S rRNA sequences is available from SILVA	- does not provide a tool for metabarcoding output
BugBase	R / Python	16S rRNA	KEGG	Functional inference	PICRUSt; custom trait assignment	Greengenes	- biologically interpretable traits (Gram staining, oxygen tolerance, biofilm formation, pathogenicity, mobile element content and oxidative stress tolerance)	- no peer-reviewed publication (biorxiv preprint)
IJSEM	flat file with R script for curation	/	IJSEM	Database	not applicable	not applicable	- 16S rRNA accession numbers available	- does not provide a tool for metabarcoding output
ProTraits	Web-based; flat files	/	Wikipedia; MicrobeWiki; HAMAP proteomes; PubMed abstracts and publications; Bacmap; Genoscope; JGI, KEGG, NCBI; Karyn's Genomes	Database	not applicable	not applicable	- phenotypic inference<br>- large ressource (~545,000 phenotypes scanning 424 traits across 3,046 species)<br>- NCBI taxonomy available	- does not provide a tool for metabarcoding output

BURRITO	Web-based	16S rRNA	KEGG Orthology	Functional inference	PICRUSt	Greengenes	- explores simultaneous and integrative studies of taxonomic and functional profiles	- based on PICRUSt v1
MACA-DAM	Python / web implementation	16S rRNA	MetaCyc, MicroCyc, FAPROTAX; IJSEM	Functional inference; Trait assignment	custom methods (provides functional information about upper-rank taxa when organism name is not found)	NCBI taxonomy	- pathway score and pathway frequency score are provided, allowing knowledge of number of enzymes present in the pathway	- not yet available for microbial eukaryotes
FunFun	R package; flat file	/	Ecological traits	Trait assignment	not applicable	based on UNITE taxonomy (ITS)	- uses R (multiplatform)<br>- complementary to FUNGuild	
Fungal-Traits	flat files	/	Guild type, body type, habitat	Trait assignment	not applicable	based on UNITE taxonomy (ITS)	- expert work to propose traits at the genus level<br>- merges the FUNGuild and FunFun tools<br>- an excel file with vlookup function is available to assign guilds or trait data	- does not provide a tool for metabarcoding output
DEEMY	Web-based	/	Morphology, anatomy, potential for chemical reactions, or even ecology traits	Database	not applicable	not applicable	- link to tree species associated<br>- includes images	- specialized in ectomycorrhizas only
Bacteria-archaea-traits	R package; flat file	16S rRNA	Traits, phenotypic traits, quantitative genomic traits	Database	not applicable	NCBI taxonomy, GTDB taxonomy	- groups the major bacterial and archaeal databases into one database<br>- traits and species data condensed<br>- R workflow available to retrieve condensed trait and species data	

OntoBio-tope	Web-based	/	Habitats and pheno-types	Database	ToMap (Text to on-tology mapping)	NCBI taxonomy	- term relevance is eval-uated by the semantic search engine PubMed-Biotope<br>- maintained by around 30 microbiology experts	- dedicated to the food domain
@Minter	Python	/	Microbial interac-tions	Machine learning	Support-vector ma-chine (SVM)-based classifier	No specific tax-onomy, just species level	- original approach to get information on microbial interactions rapidly	- species name required

1367

1368

Click here to access/download;Table;final_tables.xlsx

Table 1

Databases	Organisms
CAZy (Carbohydrate-Active Enzymes)	Eukaryotes (344), Bacteria (20,421), Archaea (413)
KEGG (Kyoto Encyclopedia of Genes and Genomes	Eukaryotes (557), Bacteria (6,317), Archaea (344)
MetaCyc (metabolic pathways and enzymes)	Total (3,295)

Table 1

Metabolic Pathways	Enzymes/Genes
NA	GH (171), GT (114), PL (41), CE (19), AA (16)
547	KEGG Orthology (KO) groups 24,402
2.937	13.356

Table 2

Tools	Implementation	Targeted genes
PanFP	Perl (recently Python)	16S rRNA
PAPRICA	Python	16S/18S rRNA
PICRUSt	Python	16S rRNA
PICRUSt2	Python / R	16S/18S rRNA/ ITS
Piphillin	Web-based	16S rRNA
SINAPS	USEARCH	16S rRNA
Tax4Fun	R package	16S rRNA
Tax4Fun2	R package	16S rRNA
Vikodak	Web-based (not longer available)	16S rRNA
iVikodak	Web-based	16S rRNA
FUNGuild	Python / Web-based	ITS
FAPROTAX	Python; flat file	16S rRNA

Table 2

BacDive	Python and R API, R package	/
BugBase	R / Python	16S rRNA
IJSEM	flat file with R script for curation	/
ProTraits	Web-based; flat files	/
BURRITO	Web-based	16S rRNA
MACADAM	Python / web implementation	16S rRNA
Fun^{Fun}	R package; flat file	/
FungalTraits	flat files	/
DEEMY	Web-based	/
Bacteria-archaea-traits	R package; flat file	16S rRNA
OntoBiotope	Web-based	/
@Minter	Python	/

Table 2

Functional Prediction	Approaches
KEGG Orthology; Gene Ontology; Pfam; TIGRFAM	Functional inference
MetaCyc ontology	Functional inference
KEGG Orthology; KEGG Pathway; COG; CAZy*	Functional inference
MetaCyc; KEGG Orthology; EC number, COGS, Pfam, TIGRFAM	Functional inference
BioCyc; KEGG	Functional inference
Trait annotation (*e.g*., energy metabolism, Gram-positive staining, presence of a flagellum)	Functional inference
KEGG Orthology	Functional inference
KEGG Orthology	Functional inference
KEGG pathway, EC number	Functional inference
KEGG; Pfam; COG; TIGRfam	Functional inference
Guild type	Trait assignment
Ecological functions (*e.g*., nitrification, denitrification or fermentation)	Trait assignment; Database

Table 2

Morphology, physiology (API®-tests), molecular data, and cultivation conditions	Database
KEGG	Functional inference
IJSEM	Database
Wikipedia; MicrobeWiki; HAMAP proteomes; PubMed abstracts and publications; Bacmap; Genoscope; JGI, KEGG, NCBI; Karyn's Genomes	Database
KEGG Orthology	Functional inference
MetaCyc, MicroCyc, FAPROTAX; IJSEM	Functional inference; Trait assignment
Ecological traits	Trait assignment
Guild type, body type, habitat	Trait assignment
Morphology, anatomy, potential for chemical reactions, or even ecology traits	Database
Traits, phenotypic traits, quantitative genomic traits	Database
Habitats and phenotypes	Database
Microbial interactions	Machine learning

Table 2

Methods
builds a pangenome
phylogenetic placement
ASR (Wagner Parsimony, ACE ML, ACE REML, ACE PIC)
HSP (maximum parcimony, empirical probabilities, subtree averaging, SCP)
Nearest-neighbor matching of 16S rRNA gene amplicons with genomes from reference databases
word counting
nearest-neighbour search based on a minimum 16S rRNA sequence similarity
BLAST
microbial co-existence patterns
microbial co-inhabitance patterns
not applicable
If all type strains of a species at the genus level share the function, FAPROTAX assumes that all uncultured organisms of this genus possess the putative function

Table 2

not applicable
PICRUSt; custom trait assignment
not applicable
not applicable
PICRUSt
custom methods (provides functional information about upper-rank taxa when organism name is not found)
not applicable
not applicable
not applicable
not applicable
ToMap (Text to ontology mapping)
Support-vector machine (SVM)-based classifier

Table 2

Inputs used	Strengths and Specificities
NCBI taxonomy	- uses functional profile of the pangenome so could be less sensitive to horizontal gene transfer
based on rDNA amplicon sequences	- 18S rRNA amplicons are taken into account<br>- examples on the developer's blog
Greengenes taxonomy (18may2012 or v13.5/v13.8)	- evolutionary models are taken into account<br>- confidence score generated (NSTI)<br>- correction of OTU copy numbers
based on rDNA amplicon sequences	- evolutionary models are taken into account<br>- confidence score generated (NSTI)<br>- twice as many KO scores<br>- multiple HSP methods can be implemented (takes branch length weighting into account)<br>- 18S rRNA and ITS amplicons are taken into account<br>- extensive documentation and active community
based on rDNA amplicon sequences	- regular updates of functional databases<br>- rRNA copy number adjustement
Greengenes; SILVA	- confidence is estimated by boostrapping<br>- integrated to USEARCH tool
SILVA taxonomy	- uses R (multiplatform) with pre-calculated files<br>- confidence score generated (FTU and FSU)<br>- the algorithm could better predict poorly characterized taxa compared to approaches based on ASR with possible large distances in the tree, thanks to a minimum of similarity between sequences
based on rDNA amplicon sequences	- algorithm with a minimal sequence similarity<br>- uses R (multiplatform) with pre-calculated, highly memory-efficient platform-independent files<br>- confidence score generated (FTU and FSU)<br>- KO update from 2018<br>- calculates the redundancy of specific functions directly<br>- builds its own habitat-specific reference
RDP, SILVA	- pathway exclusion cut-off value is available to provide the minimum percentage of genes/enzymes belonging to a metabolic pathway required to consider the pathway as functional.<br>- compares two datasets
RDP, Greengenes, SILVA	- user-friendly for non-expert bioinformaticians<br>- integrated tools for statistical comparisons<br>- graphical visualizations
based on UNITE taxonomy (ITS)	- trait quality for taxon assignment
SILVA (128, 132)	- based on the literature of cultured taxa<br>- availability of all literature to create the database<br>- functions easily added to the tool

Table 2

NCBI taxonomy	- provides links to ENA, GenBank, SILVA, BRENDA, GBIF, ChEBI, Straininfo website data<br>- a match with 16S rRNA sequences is available from SILVA
Greengenes	- biologically interpretable traits **(**Gram staining, oxygen tolerance, biofilm formation, pathogenicity, mobile element content and oxidative stress tolerance**)**
not applicable	- 16S rRNA accession numbers available
not applicable	- phenotypic inference<br>- large ressource (~545,000 phenotypes scanning 424 traits across 3,046 species)<br>- NCBI taxonomy available
Greengenes	- explores simultaneous and integrative studies of taxonomic and functional profiles
NCBI taxonomy	- pathway score and pathway frequency score are provided, allowing knowledge of number of enzymes present in the pathway
based on UNITE taxonomy (ITS)	- uses R (multiplatform)<br>- complementary to FUNGuild
based on UNITE taxonomy (ITS)	- expert work to propose traits at the genus level<br>- merges the FUNGuild and FunFun tools<br>- an excel file with vlookup function is available to assign guilds or trait data
not applicable	- link to tree species associated<br>- includes images
NCBI taxonomy, GTDB taxonomy	- groups the major bacterial and archaeal databases into one database<br>- traits and species data condensed<br>- R workflow available to retrieve condensed trait and species data
NCBI taxonomy	- term relevance is evaluated by the semantic search engine PubMedBiotope<br>- maintained by around 30 microbiology experts
No specific taxonomy, just species level	- original approach to get information on microbial interactions rapidly

Limitations
- evolutionary models are not taken into account<br>- no confidence score generated<br>- not yet available for microbial eukaryotes
- errors may occur with sequence placement due to poor resolution of rRNA amplicons in some clades
- based on specific taxonomy (GreenGenes identifiers)<br>- KEGG database not updated since 2011<br>- no pre-calculated table of fungal genomes available
- errors may occur with sequence placement due to poor resolution of rRNA amplicons in some clades
- available online only<br>- available for 16S rRNA only
- no peer-reviewed publication (biorxiv preprint)<br>- detailed explanation is missing (*e.g* ., how was protrait input created?)
- based on specific taxonomy (SILVA identifiers)<br>- KEGG database not updated since 2011
- not yet available for microbial eukaryotes
- not longer available<br>- not yet available for microbial eukaryotes
- available online only<br>- not yet available for microbial eukaryotes
- no regular update<br>- 18S rRNA taxonomy with related database not included. However, the database is open-access, and a homemade wrapper can be used for 18S metabarcoding output
- implicit assumption (see algorithm column) could be false with the increase of newly cultured organisms<br>- does not infer upper rank when taxonomic resolution is poor

Table 2

- does not provide a tool for metabarcoding output
- no peer-reviewed publication (biorxiv preprint)
- does not provide a tool for metabarcoding output
- does not provide a tool for metabarcoding output
- based on PICRUSt v1
- not yet available for microbial eukaryotes
- does not provide a tool for metabarcoding output
- specialized in ectomycorrhizas only
- dedicated to the food domain
- species name required

Figures

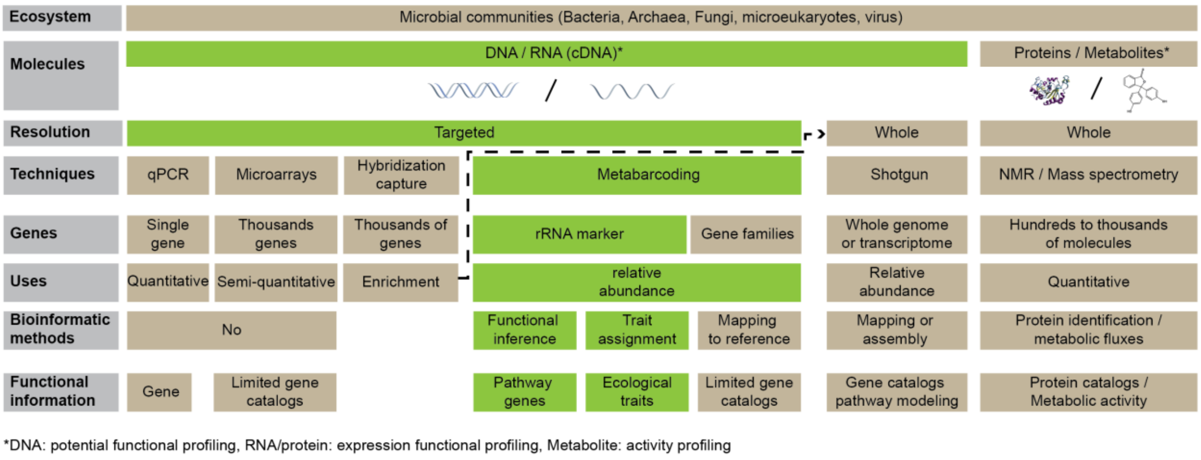

Figure 1: **Schematic diagram of the various strategies available for exploring the functional diversity of the microbiota**. Green frames, metabarcoding approaches for retrieving putative functions from taxonomic genes by functional inference and ecological trait assignment.

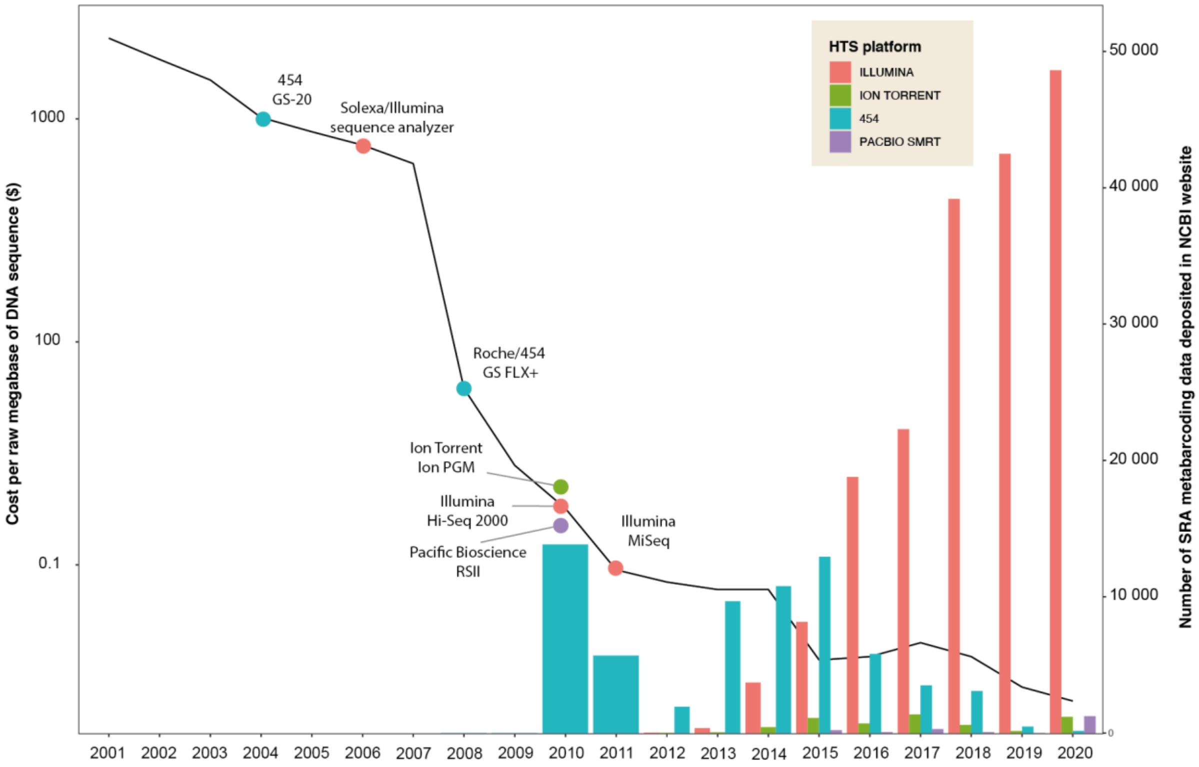

Figure 2: **Evolution of costs (dollars) *per* raw megabase of DNA sequence (black line with logarithmic scale), and evolution of the number of SRA metabarcoding data deposited in the NCBI website.** The data used to draw this figure is described in Additional file 1.

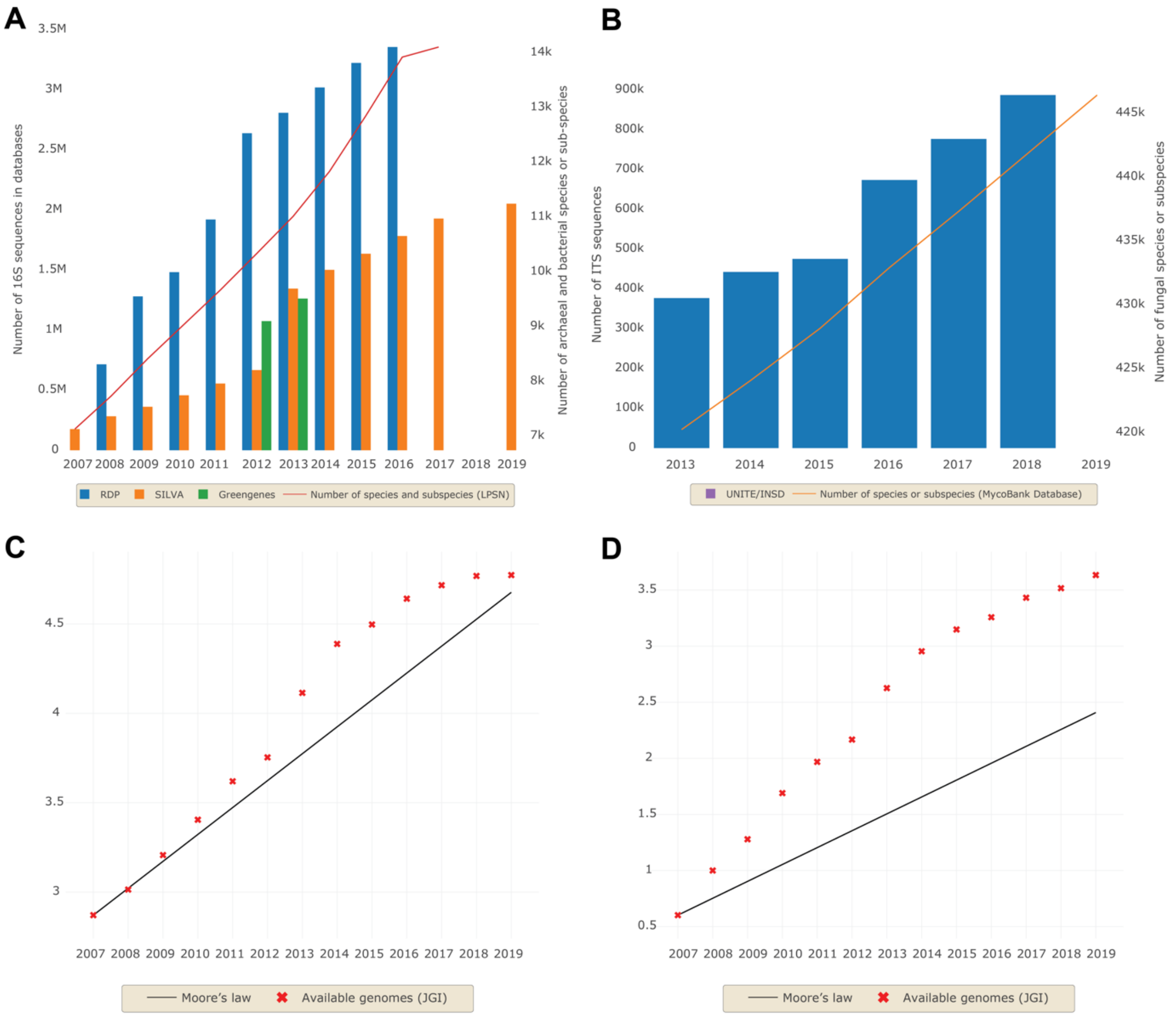

Figure 3: **Annual cumulative growth of databases in terms of bacterial/archaeal (A) and fungal (B) sequences, and species/subspecies deposited *per* year. Comparison of the annual cumulative growth of bacterial/archaeal (C) and fungal (D) genomes compared to simulations of Moore's law**. The plot is in logarithmic scale. Three databases were compared for 16S rRNA gene sequences: RDP (blue), SILVA (orange), Greengenes (green). Information is based on the List of Prokaryotic names with Standing in Nomenclature (LPSN [125], http://www.bacterio.net) website for bacterial and archaeal species, and on the MycoBank database for fungal species ([126], http://www.mycobank.org). Information about the bacterial, archaeal and fungal genomes is based on the Genome OnLine Database (GOLD) [127].

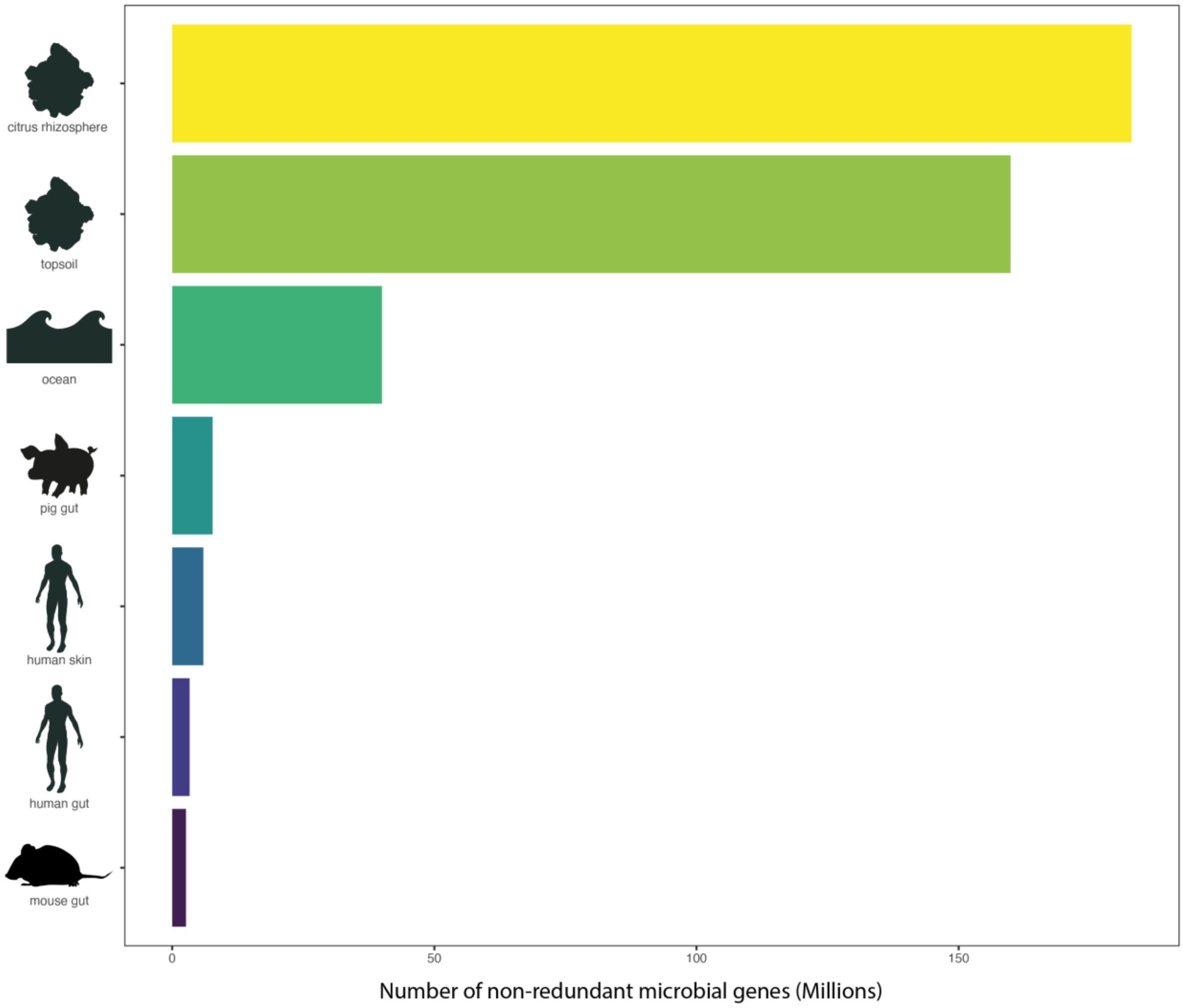

Figure 4: **Global microbial gene catalogs from various ecosystems. The** references are listed in Additional file 1.

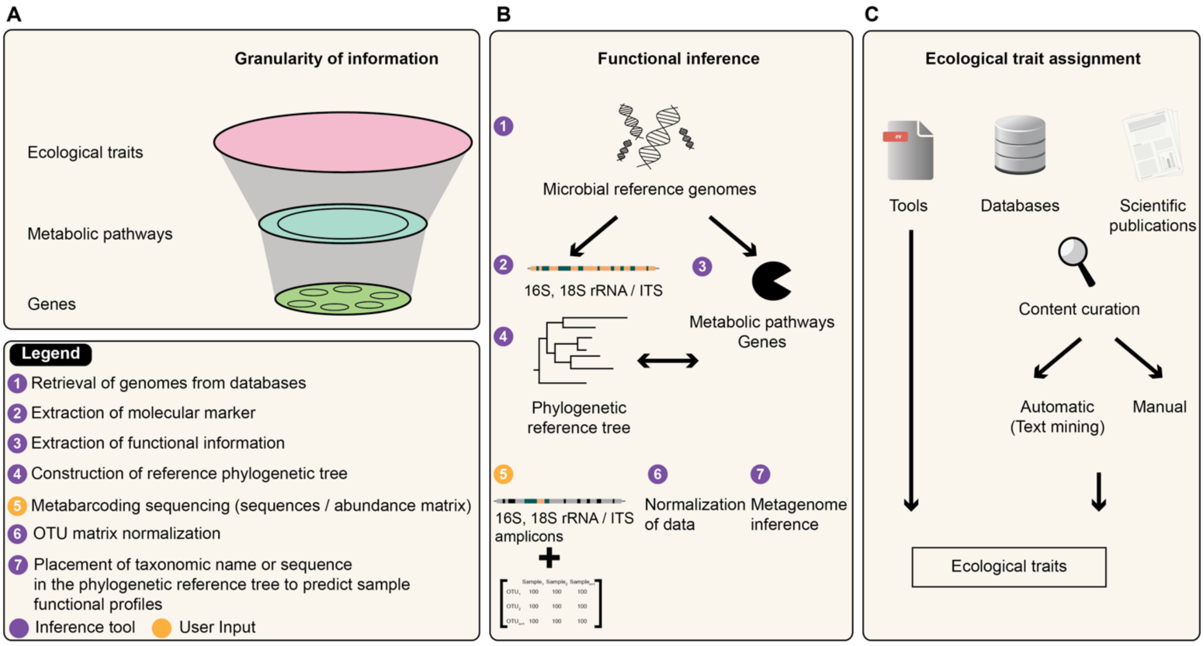

Figure 5: **Diagram of the granularity of the data (A) that can be obtained by functional inference (B) or ecological trait assignment (C)**.

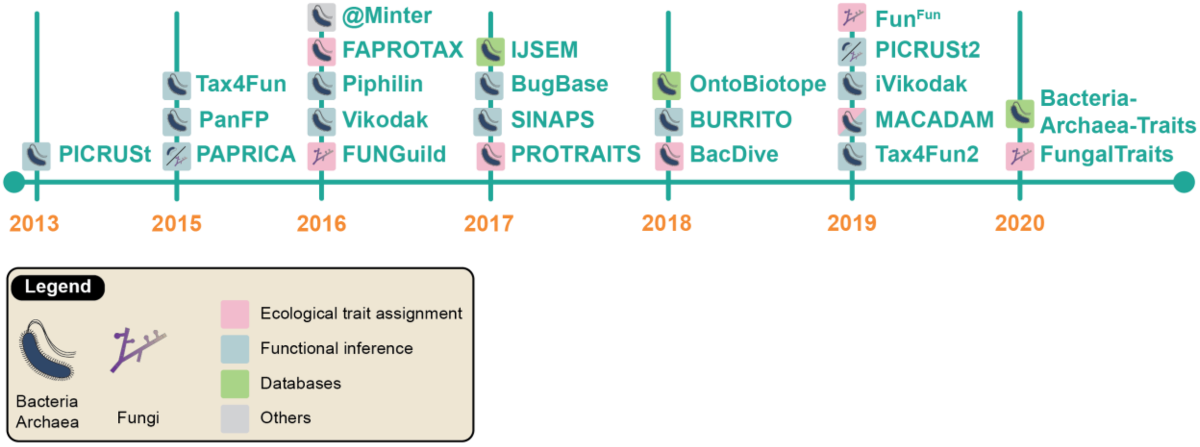

Figure 6: **Timeline depicting the historical record of the major tools developed for functional inference or ecological trait assignment.** The first version of the DEEMY database dates back to 1996; it was not included for aesthetic reasons.

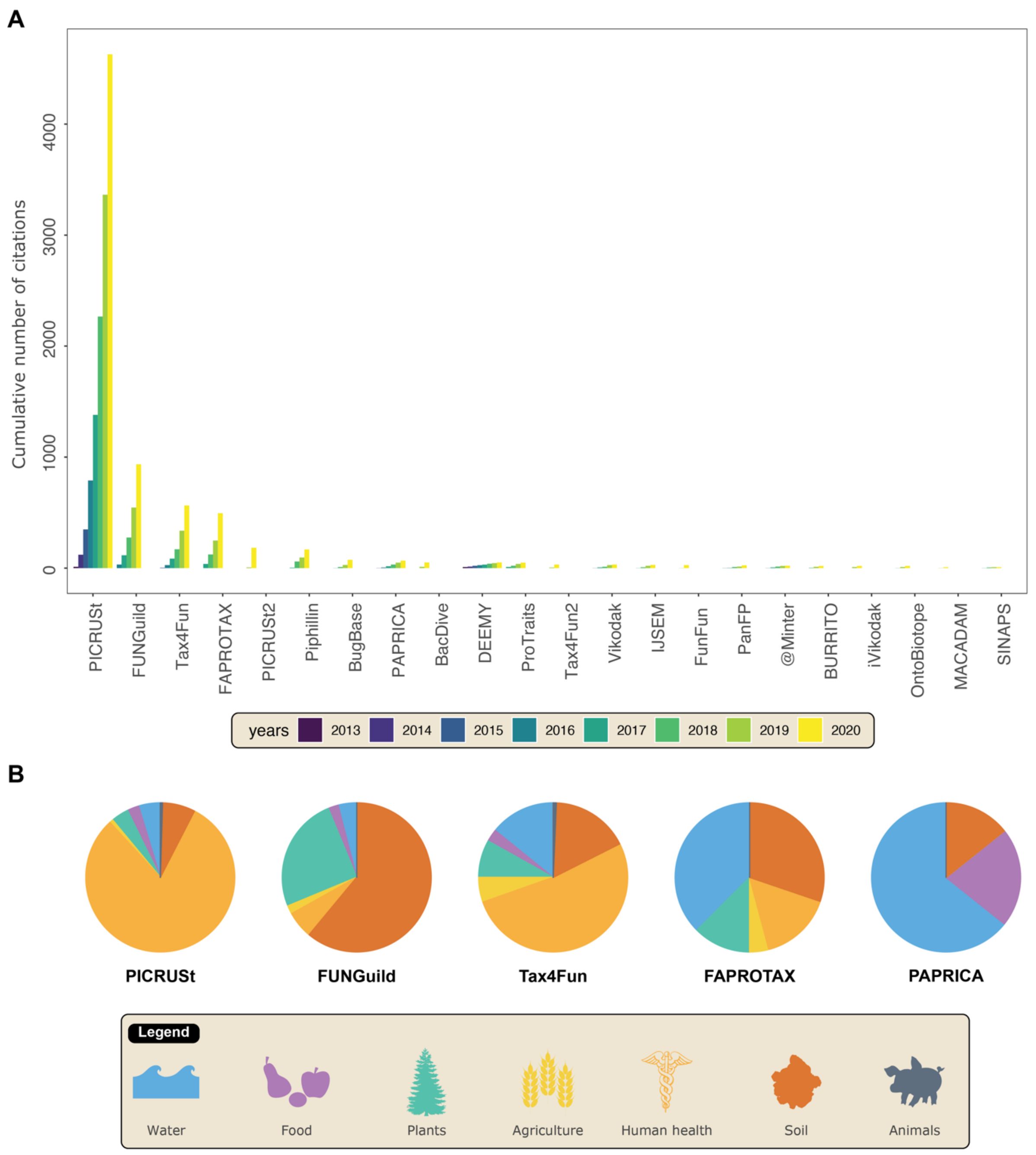

Figure 7: **Annual cumulative number of citations of the major tools (A) and their scope (B).** The keywords used for “scope” were retrieved from the titles and abstracts of the papers listed in Additional file 1.

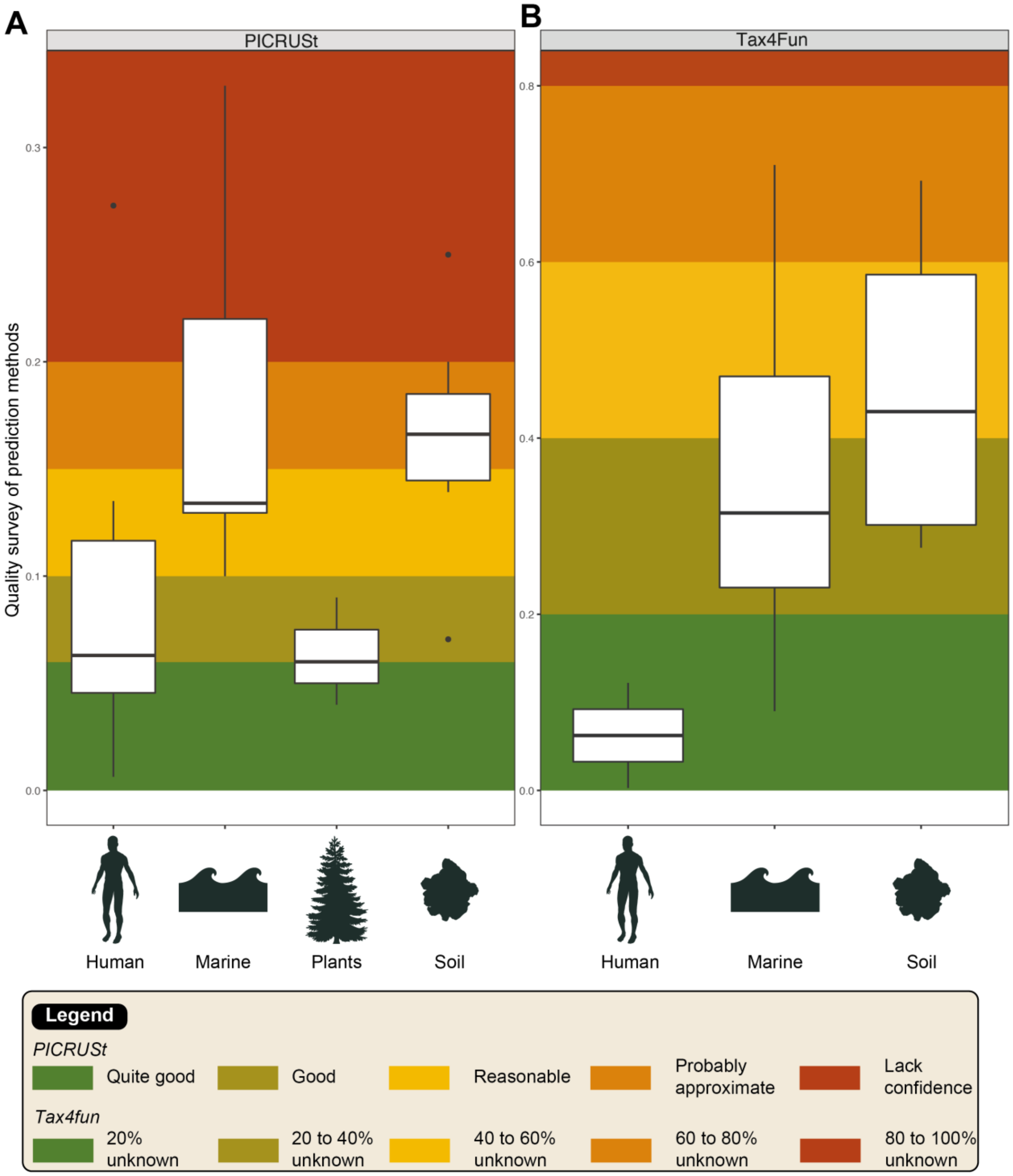

Figure 8: **Overview of the quality of functional prediction based on a subsampling of articles for PICRUSt (A) and Tax4Fun (B) across various ecosystems.** For PICRUSt, colors were assigned according NSTI results: < 0.06, quite good; 0.06 to 0.10, good; 0.10 to 0.15, reasonable but probably approximate; and > 0.20, probably unreliable. For Tax4Fun, we split the fraction of OTUs that could not be mapped to KEGG organisms in 5 harmonious groups. References are listed in Additional file 1.

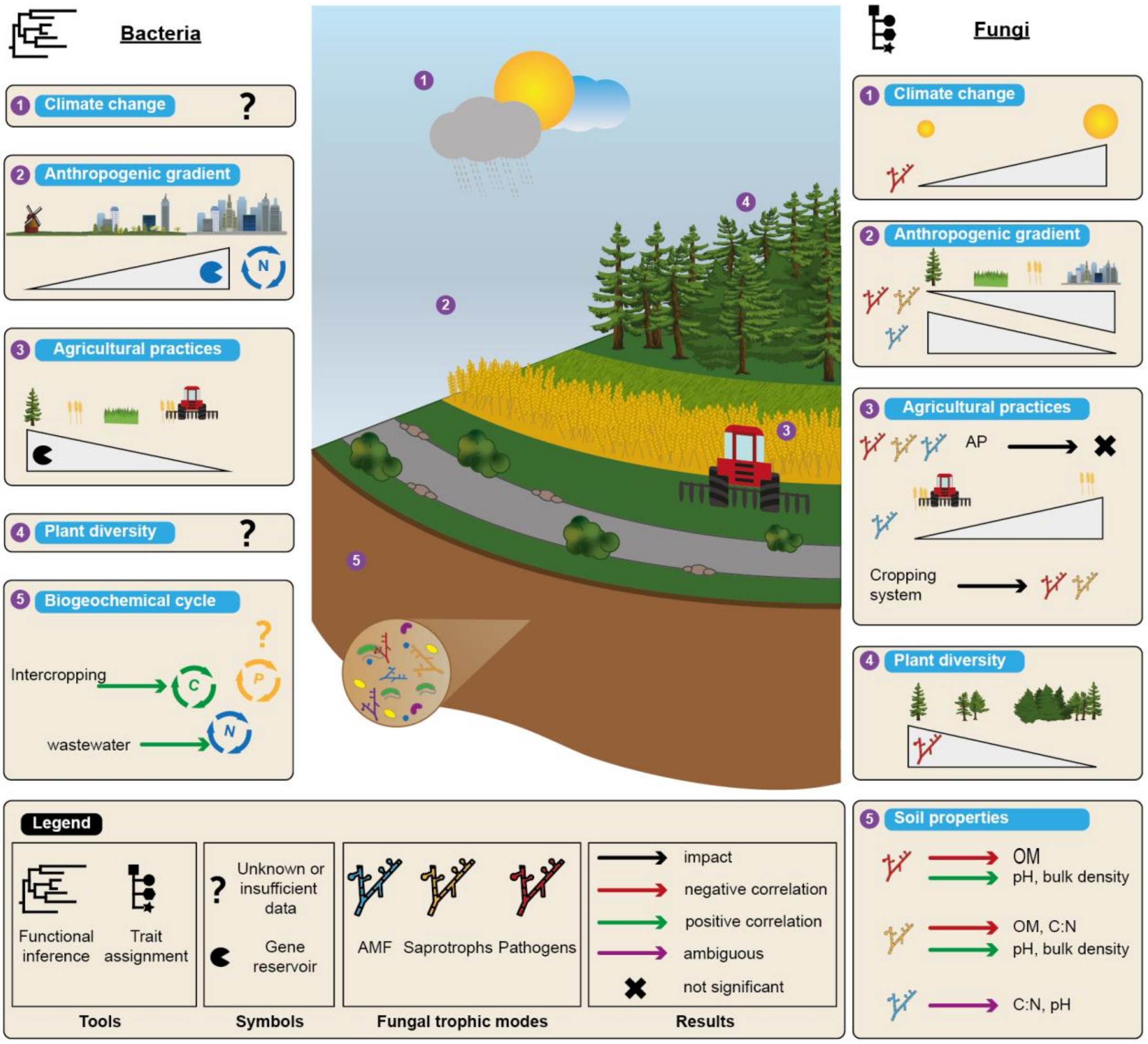

Figure 9: **Summary diagram of the most relevant microbial soil functions results based on functional inference and ecological trait assignment.**

The figure is made up of two parts: studies on bacterial communities based on functional inference on the left, and studies on fungal communities based on ecological trait assignment on the right. For all studies (climate change, anthropogenic gradient, agricultural practices, plant diversity or the biogeochemical cycle), if an impact or a correlation was found on the gene reservoir or on microbial communities with a particular ecological trait, a colored arrow indicates the effect and a cross indicates no significant effect. A triangle indicates either a decrease or an increase of the gene reservoir or microbial communities with a particular trait. References are listed in Additional file 1.

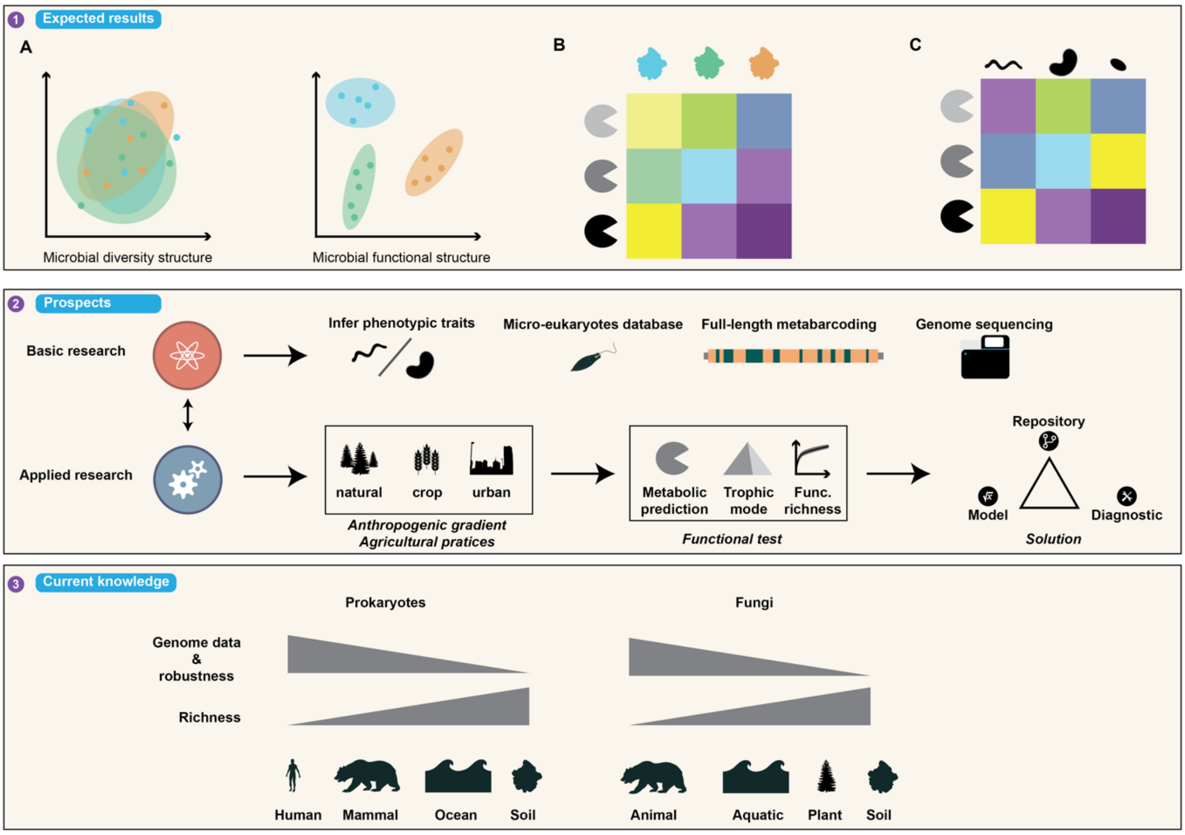

Figure 10: **Summary diagram of the expected results (first box), the functional prediction prospects (second box) and the limits of the microbial genomic data available for different habitats (third box).** The first box illustrates a comparative example of data results of community structures and functional structures through a PCA (A). This example illustrates the case when the functional community structure differentiates experimental conditions better than it differentiates the microbial community structure. Illustrative heat maps showing the relative abundance of genes *per* sample (B) or *per* OTU (C).

Additional File 1

Click here to access/download
Supplementary Material
Additional_file_1.docx

Cover letter Click here to access/download;Personal Cover;cover-letter.docx

Agroécologie Dijon Unité de Recherche AGRO SUP DIJON INRAE UBFC UNIVERSITE BOURGOGNE FRANCHE-COMTE

UMR 1347 - AGROECOLOGIE

RANJARD Lionel (BIOCOM Team)
Phone. : 33 (0) 03 80 69 30 88
Mail : lionel.ranjard@inrae.fr

Dijon, 04 October 2021

Dear Editor,

We would like to submit the paper entitled "Inferring microbiota functions from taxonomic genes: a review" by Christophe Djemiel, Pierre-Alain Maron, Sébastien Terrat, Samuel Dequiedt, Aurélien Cottin, and Lionel Ranjard for publication in *GigaScience*.

In this paper we review the tools and methods dedicated to functional inference and ecological trait assignment to explore the functional potential of microbial ecosystems. These approaches have been developed after the recent surge of big data in microbial ecology studies thanks to high-throughput sequencing. Some tools have become quite popular thanks to the popularization of metabarcoding, but studies allowing an overview, an evaluation and a ranking of the advantages, specificities and drawbacks of these tools are still blatantly lacking in current literature, both for bacterial and fungal communities.

Overall, our scientific and technical benchmarks show that functional inference and trait assignment are powerful methods for describing changes in the functional potential of complex microbial communities metabarcoding approaches. However, making them a robust diagnostic tool in various fields (*e.g*. soil studies) still remains a challenge.

We believe that this work will help scientists working on microbial communities make the appropriate choices to best take advantage of the high amounts of microbial data made available.

The work presented in this manuscript is original and has not been published or considered for publication by another journal.

We thank you for considering this manuscript for publication in *GigaScience*.

Yours sincerely,

Lionel RANJARD and Christophe DJEMIEL

 Click here to access/download;Personal Cover;Answer_to_reviewers.docx

10-November-2021

GIGA-D-21-00316Inferring microbiota functions from taxonomic genes: a review

Dear Dr Ranjard,
Your manuscript "Inferring microbiota functions from taxonomic genes: a review" (GIGA-D-21-00316) has been assessed by our reviewers. Based on these reports, and my own assessment as Editor, I am pleased to inform you that it is potentially acceptable for publication in GigaScience, once you have carried out some essential revisions suggested by our reviewers.
Their reports, together with any other comments, are below. Please also take a moment to check our website at https://www.editorialmanager.com/giga/ for any additional comments that were saved as attachments.
In addition, please register any new software application in the bio.tools and SciCrunch.org databases to receive RRID (Research Resource Identification Initiative ID) and biotoolsID identifiers, and include these in your manuscript. This will facilitate tracking, reproducibility and re-use of your tool.
Once you have made the necessary corrections, please submit a revised manuscript online at: https://www.editorialmanager.com/giga/
If you have forgotten your username or password please use the "Send Login Details" link to get your login information. For security reasons, your password will be reset.
Please include a point-by-point within the 'Response to Reviewers' box in the submission system. Please ensure you describe additional experiments that were carried out and include a detailed rebuttal of any criticisms or requested revisions that you disagreed with. Please also ensure that your revised manuscript conforms to the journal style, which can be found in the Instructions for Authors on the journal homepage. If the data and code has been modified in the revision process please be sure to update the public versions of this too.
The due date for submitting the revised version of your article is 31 Jan 2022. We look forward to receiving your revised manuscript soon.
Best wishes,
Nicole Nogoy, Ph.D
GigaScience

Reviewer reports:
Reviewer #1: This review manuscript provides an overview of the various options that have been developed for inferring function from taxonomic data, for Bacteria, Archaea and Fungi. It should be a good resource and introduction to the topic. One aspect that could be developed a bit more is that of specificity in functional prediction.

First of all, we want to thank the Reviewer 1 for these commentaries and he pinpoints some drawbacks to help us improving it.

The specific corrections, and modifications advised by the Reviewer 1 will be highlighted in Green in the manuscript.

Throughout, please change "consists in" to "consists of"
As requested, we did the correction, and checked in the whole manuscript (lines 205, 239, 258, 330).

Line 113: It is unclear why soil quality is mentioned here
Indeed, we chose to delete the "soil quality" term to avoid focusing our sentence only just on soil diagnosis, keeping only the global point of view in this background section. We also changed the next sentence in order to ensure consistency ("A repository" to "Repositories").

Line 145: Change "ocean" to "marine" or "aquatic"?
As requested, we did the correction in the sentence.

Line 148: Change "rDNA" to "rRNA gene"
As requested, we did the correction in the sentence.

Line 219: The comparison here to shotgun metagenomics should consider the impact of horizontal gene transfer and gene loss.
As requested, we have added a sentence to indicate that inferred metagenomes do not allow the study of lateral gene transfer and gene loss unlike shotgun metagenomics. In addition, only archaea, bacteria, and fungi can be studied directly by these tools, unlike shotgun metagenomics which provides an overview of all microbial communities.

Similarly, Line 545 should consider gene deletion in addition to horizontal gene transfer.
As requested, we have completed our sentence to indicate that gene gain and loss was also due to gene duplication, gene loss, and de novo gene birth in addition to predominant mode HGT. We have also added bibliographic references regarding the identification of HGT from shotgun metagenomic data.

Line 552: In addition to plasmid transfer, should consider phage / viruses
As requested, we have added a sentence to complete our paragraph on the transfer of plasmids by phages or viruses.

Line 665: It could be instructive to provide more examples of practical applications
Indeed, we were in the same expectation as reviewer 1 but there are very few examples of practical applications. Moreover, this is what we underline throughout our manuscript. However, as requested, we added two examples in the human health on the search for cancer biomarkers and two examples on the biomonitoring of water quality. We have also added bibliographic references regarding these practical applications.

Line 699: It is not clear why these particular soil measurements are of interest
Yes, we fully agree with the reviewer 1. As requested, we added a sentence to precise the definition of the volatile organic compound in order clear why it is interesting to use these soil measurements. We also added VOC abbreviation in the section Abbreviations.

Figure 1: Change "Functional inferance" to "Functional inference"
As requested, we did the correction in the figure 1.

Figure 2: Add data for most recent years?
Yes, we fully agree, it is not intended and we added data for 2018, 2019 and 2020 years in the figure 2.

Figure 9: Change "Unknow or few data" to "Unknown or insufficient data"
As requested, we did the correction in the figure 9.

Reviewer: 2
Reviewer #2: The authors presented a review about inferring microbiota functions from taxonomic genes. In general, this topic is very important to gain more insights from amplicon based sequencing strategies to decipher the role of the microbiome. The manuscript is clearly written and the authors present nicely the state of current tools.
I have just a couple of minor comments that should be addressed.

First of all, we want to thank the Reviewer 2 for these commentaries and he pinpoints some drawbacks to help us improving it.

The specific corrections, and modifications advised by the Reviewer 2 will be highlighted in Blue in the manuscript.

I wonder whether the author can mention PICRUSt2 earlier in the manuscript as it is a great improvement compared to v1. I would suggest to add one sentence in the section about PICRUSt v1.
As requested, we have added sentence to describe the main improvements of PICRUSt2 following the paragraph of PICRUSt1 (L271).

[L176] @MInter should be spelled correctly.
As requested, we did the correction in the sentence.

[L224] Picrust should be written PICRUSt
As requested, we did the correction in the sentence.

[L421] The author write 'No tool...' but I guess it should be 'The tool...'
Indeed, it is a clerical error. We did the correction in the sentence.

[L508] A space is missing ('...fungi - along with...')
As requested, we added the space.
