# Supplementary material for: Inferring microbiota functions from taxonomic genes: a review
Source: Gigascience. 2022 Jan 12;11:giab090. doi: 10.1093/gigascience/giab090 (PMC8756179; doi:10.1093/gigascience/giab090)
Supplement: giab090_Supplemental_File [file giab090_supplemental_file.docx]

**Inferring microbiota functions from taxonomic genes: a review**

Christophe Djemiel^1^, Sébastien Terrat^1^, Samuel Dequiedt^1^, Aurélien Cottin^1^, Pierre-Alain Maron^1^, Lionel Ranjard^1^

Authors’ affiliations

^1^ Agroécologie, AgroSup Dijon, INRAE, Univ. Bourgogne, Univ. Bourgogne Franche-Comté, F-21000 Dijon, France.

This section explains the construction of figures present in the main manuscript.

**Figure 2**

The cost data was downloaded from <https://www.genome.gov/about-genomics/fact-sheets/DNA-Sequencing-Costs-Data>, and the SRA data directly from the NCBI website with the following request ((((metabarcoding) OR targeted metagenomics) OR targeted metagenomic) OR targeted-metagenomics) OR metataxonomic (April 2021). The main HTS instruments are placed on the black line indicating their commercialization date.

**Figure 3**

| Date | SILVA database SSUref | UNITE+INSD |
| --- | --- | --- |
| 2007 | r92 | NA |
| 2008 | r96 | NA |
| 2009 | r100 | NA |
| 2010 | r104 | NA |
| 2011 | r108 | NA |
| 2012 | r111 | NA |
| 2013 | r115 | 6.0 |
| 2014 | r119 | 6.0 |
| 2015 | r123 | 7.0 |
| 2016 | r128 | 7.1 |
| 2017 | r132 | 7.2 |
| 2018 | NA | 8.0 |
| 2019 | r138 | NA |

The numbers of fungal species and subspecies were download from <https://www.mycobank.org/page/Stats%20page> (July 2021).

**Figure 4**

| Habitat | Reference |
| --- | --- |
| Human gut | [1] |
| Human skin | [2] |
| Mouse gut | [3] |
| Pig gut | [4] |
| Ocean | [5] |
| Topsoil | [6] |
| Citrus rhizosphere | [7] |

**Figure 7**

| Scope | Keywords |
| --- | --- |
| Human health | Human, health, disease, asthma, infancy, childhood, vaginal, intestinal, diet, gut |
| Soil | Soil |
| Plant | Plant |
| Water | Water, marine |
| Agricultural | Agricultural |
| Animal | Animal, murine |
| Food | food |

**Figure 8**

| Tools | Habitat | Reference |
| --- | --- | --- |
| PICRUSt | Soil | [8–11] |
| PICRUSt | Human | [10,12–14] |
| PICRUSt | Marine | [10,15–17] |
| PICRUSt | Plant | [11,18] |
| Tax4fun | Soil | [10,19] |
| Tax4fun | Human | [10] |
| Tax4fun | Marine | [10,20,21] |

**Figure 9**

| Tools | Habitat | Reference |
| --- | --- | --- |
| Functional Inference | Climate change | NA |
| Functional Inference | Anthropogenic gradient | [19] |
| Functional Inference | Agricultural practices | [22] |
| Functional Inference | Plant diversity | NA |
| Functional Inference | Biogeochemical cycle | [23,24] |
| Ecological traits | Climate change | [25] |
| Ecological traits | Anthropogenic gradient | [26] |
| Ecological traits | Agricultural practices | [27] |
| Ecological traits | Plant diversity | [28] |
| Ecological traits | Soil properties | [29,30] |

**References**

1. Qin J, Li R, Raes J, Arumugam M, Burgdorf KS, Manichanh C, et al. A human gut microbial gene catalogue established by metagenomic sequencing. Nature [Internet]. 2010;464:59–65. Available from: http://www.nature.com/articles/nature08821

2. Oh J, Byrd AL, Deming C, Conlan S, Kong HH, Segre JA. Biogeography and individuality shape function in the human skin metagenome. Nature [Internet]. 2014;514:59–64. Available from: http://www.nature.com/articles/nature13786

3. Xiao L, Feng Q, Liang S, Sonne SB, Xia Z, Qiu X, et al. A catalog of the mouse gut metagenome. Nat Biotechnol [Internet]. 2015 [cited 2016 Jul 26];33:1103–8. Available from: http://www.ncbi.nlm.nih.gov/pubmed/26414350

4. Xiao L, Estellé J, Kiilerich P, Ramayo-Caldas Y, Xia Z, Feng Q, et al. A reference gene catalogue of the pig gut microbiome. Nat Microbiol [Internet]. 2016;1:16161. Available from: http://www.nature.com/articles/nmicrobiol2016161

5. Sunagawa S, Coelho LP, Chaffron S, Kultima JR, Labadie K, Salazar G, et al. Structure and function of the global ocean microbiome. Science (80- ) [Internet]. 2015;348:1261359–1261359. Available from: https://www.ncbi.nlm.nih.gov/pubmed/25999513

6. Bahram M, Hildebrand F, Forslund SK, Anderson JL, Soudzilovskaia NA, Bodegom PM, et al. Structure and function of the global topsoil microbiome. Nature [Internet]. 2018;560:233–7. Available from: http://www.nature.com/articles/s41586-018-0386-6

7. Xu J, Zhang Y, Zhang P, Trivedi P, Riera N, Wang Y, et al. The structure and function of the global citrus rhizosphere microbiome. Nat Commun [Internet]. 2018;9:4894. Available from: http://www.nature.com/articles/s41467-018-07343-2

8. Mushinski RM, Zhou Y, Gentry TJ, Boutton TW. Bacterial metataxonomic profile and putative functional behavior associated with C and N cycle processes remain altered for decades after forest harvest. Soil Biol Biochem [Internet]. 2018;119:184–93. Available from: https://linkinghub.elsevier.com/retrieve/pii/S0038071718300087

9. Hariharan J, Sengupta A, Grewal P, Dick WA. Functional Predictions of Microbial Communities in Soil as Affected by Long‐term Tillage Practices. Agric Environ Lett [Internet]. 2017;2:170031. Available from: https://onlinelibrary.wiley.com/doi/10.2134/ael2017.09.0031

10. Aßhauer KP, Wemheuer B, Daniel R, Meinicke P. Tax4Fun: Predicting functional profiles from metagenomic 16S rRNA data. Bioinformatics [Internet]. 2015 [cited 2015 May 13];31:2882–4. Available from: http://www.pubmedcentral.nih.gov/articlerender.fcgi?artid=4547618&tool=pmcentrez&rendertype=abstract

11. Zarraonaindia I, Owens SM, Weisenhorn P, West K, Hampton-Marcell J, Lax S, et al. The soil microbiome influences grapevine-associated microbiota. Jansson JK, editor. MBio [Internet]. 2015;6. Available from: https://journals.asm.org/doi/10.1128/mBio.02527-14

12. Parras-Moltó M, de Cárcer DA. A comprehensive human minimal gut metagenome extends the host’s metabolic potential. Microb Genomics [Internet]. 2020;6:1–7. Available from: https://www.microbiologyresearch.org/content/journal/mgen/10.1099/mgen.0.000466

13. Peng X, Zhou L, Gong Y, Song Z, He L, Lin S, et al. Non-pylori Helicobacters (NHPHs) Induce Shifts in Gastric Microbiota in Helicobacter pylori-Infected Patients. Front Microbiol [Internet]. 2017;8. Available from: http://journal.frontiersin.org/article/10.3389/fmicb.2017.01038/full

14. Park CH, Han DS, Oh YH, Lee AR, Lee YR, Eun CS. Role of Fusobacteria in the serrated pathway of colorectal carcinogenesis. Sci Rep [Internet]. 2016;6:25271. Available from: http://www.nature.com/articles/srep25271

15. Wang K, Ye X, Zhang H, Chen H, Zhang D, Liu L. Regional variations in the diversity and predicted metabolic potential of benthic prokaryotes in coastal northern Zhejiang, East China Sea. Sci Rep [Internet]. 2016;6:38709. Available from: http://www.nature.com/articles/srep38709

16. Koo H, Mojib N, Hakim JA, Hawes I, Tanabe Y, Andersen DT, et al. Microbial Communities and Their Predicted Metabolic Functions in Growth Laminae of a Unique Large Conical Mat from Lake Untersee, East Antarctica. Front Microbiol [Internet]. 2017;8. Available from: http://journal.frontiersin.org/article/10.3389/fmicb.2017.01347/full

17. Raes EJ, Karsh K, Sow SLS, Ostrowski M, Brown M V., van de Kamp J, et al. Metabolic pathways inferred from a bacterial marker gene illuminate ecological changes across South Pacific frontal boundaries. Nat Commun [Internet]. 2021;12:2213. Available from: http://www.nature.com/articles/s41467-021-22409-4

18. Furtado BU, Gołębiewski M, Skorupa M, Hulisz P, Hrynkiewicz K. Bacterial and Fungal Endophytic Microbiomes of Salicornia europaea. Master ER, editor. Appl Environ Microbiol [Internet]. 2019;85. Available from: https://journals.asm.org/doi/10.1128/AEM.00305-19

19. Gonzalez Mateu M, Park C, McAskill C, Baldwin A, Yarwood S. Urbanization Altered Bacterial and Archaeal Composition in Tidal Freshwater Wetlands Near Washington DC, USA, and Buenos Aires, Argentina. Microorganisms [Internet]. 2019;7:72. Available from: https://www.mdpi.com/2076-2607/7/3/72

20. Steinert G, Wemheuer B, Janussen D, Erpenbeck D, Daniel R, Simon M, et al. Prokaryotic diversity and community patterns in antarctic continental shelf sponges. Front Mar Sci [Internet]. 2019;6. Available from: https://www.frontiersin.org/article/10.3389/fmars.2019.00297/full

21. Pavloudi C, Kristoffersen JB, Oulas A, De Troch M, Arvanitidis C. Sediment microbial taxonomic and functional diversity in a natural salinity gradient challenge Remane’s “species minimum” concept. PeerJ [Internet]. 2017;5:e3687. Available from: https://peerj.com/articles/3687

22. Sengupta A, Hariharan J, Grewal PS, Dick WA. Bacterial community dissimilarity in soils is driven by long‐term land‐use practices. Agrosystems, Geosci Environ. 2020;3.

23. Lian T, Mu Y, Jin J, Ma Q, Cheng Y, Cai Z, et al. Impact of intercropping on the coupling between soil microbial community structure, activity, and nutrient-use efficiencies. PeerJ [Internet]. 2019;7:e6412. Available from: https://peerj.com/articles/6412

24. Hartman WH, Ye R, Horwath WR, Tringe SG. A genomic perspective on stoichiometric regulation of soil carbon cycling. ISME J [Internet]. 2017;11:2652–65. Available from: http://www.nature.com/articles/ismej2017115

25. Delgado-Baquerizo M, Guerra CA, Cano-Díaz C, Egidi E, Wang J-T, Eisenhauer N, et al. The proportion of soil-borne pathogens increases with warming at the global scale. Nat Clim Chang [Internet]. 2020;10:550–4. Available from: http://www.nature.com/articles/s41558-020-0759-3

26. Brinkmann N, Schneider D, Sahner J, Ballauff J, Edy N, Barus H, et al. Intensive tropical land use massively shifts soil fungal communities. Sci Rep [Internet]. 2019;9:3403. Available from: http://www.nature.com/articles/s41598-019-39829-4

27. Legrand F, Picot A, Cobo-Díaz JF, Carof M, Chen W, Le Floch G. Effect of tillage and static abiotic soil properties on microbial diversity. Appl Soil Ecol [Internet]. 2018;132:135–45. Available from: https://linkinghub.elsevier.com/retrieve/pii/S0929139318301859

28. Liang M, Liu X, Parker IM, Johnson D, Zheng Y, Luo S, et al. Soil microbes drive phylogenetic diversity-productivity relationships in a subtropical forest. Sci Adv [Internet]. 2019;5:eaax5088. Available from: https://advances.sciencemag.org/lookup/doi/10.1126/sciadv.aax5088

29. George PBL, Creer S, Griffiths RI, Emmett BA, Robinson DA, Jones DL. Primer and Database Choice Affect Fungal Functional but Not Biological Diversity Findings in a National Soil Survey. Front Environ Sci [Internet]. 2019;7. Available from: https://www.frontiersin.org/article/10.3389/fenvs.2019.00173/full

30. Anthony MA, Frey SD, Stinson KA. Fungal community homogenization, shift in dominant trophic guild, and appearance of novel taxa with biotic invasion. Ecosphere [Internet]. 2017;8:e01951. Available from: http://doi.wiley.com/10.1002/ecs2.1951
