# Supplementary material for: Inferring microbiota functions from taxonomic genes: a review
Source: Gigascience. 2022 Jan 12;11:giab090. doi: 10.1093/gigascience/giab090 (PMC8756179; doi:10.1093/gigascience/giab090)
Supplement: giab090_Response_to_Reviewer_Comments_Original_Submission [file giab090_response_to_reviewer_comments_original_submission.pdf]

Author's Response To Reviewer Comments

Close

Dear Editor,

Please find enclosed the revised version of the paper Submission ID GIGA-D-21-00316 Inferring microbiota functions from taxonomic genes: a review by Christophe Djemiel, Pierre-Alain Maron, Sébastien Terrat, Samuel Dequiedt, Aurélien Cottin, Lionel Ranjard.
We answered carefully to the all comments of the reviewer 1 and 2. We thank the 2 reviewers, for their helpful comments during the reviewing process. We hope that the paper is now suitable for publication in GigaScience, and we look forward to hearing from you.
Kind regards
Christophe Djemiel & Lionel Ranjard

Reviewer reports:
Reviewer #1: This review manuscript provides an overview of the various options that have been developed for inferring function from taxonomic data, for Bacteria, Archaea and Fungi. It should be a good resource and introduction to the topic. One aspect that could be developed a bit more is that of specificity in functional prediction.

First of all, we want to thank the Reviewer 1 for these commentaries and he pinpoints some drawbacks to help us improving it.

The specific corrections, and modifications advised by the Reviewer 1 will be highlighted in Green in the manuscript.

Throughout, please change "consists in" to "consists of"
As requested, we did the correction, and checked in the whole manuscript (lines 205, 239, 258, 330).

Line 113: It is unclear why soil quality is mentioned here
Indeed, we chose to delete the "soil quality" term to avoid focusing our sentence only just on soil diagnosis, keeping only the global point of view in this background section. We also changed the next sentence in order to ensure consistency (“A repository” to “Repositories”).

Line 145: Change "ocean" to "marine" or "aquatic"?
As requested, we did the correction in the sentence.

Line 148: Change "rDNA" to "rRNA gene"
As requested, we did the correction in the sentence.

Line 219: The comparison here to shotgun metagenomics should consider the impact of horizontal gene transfer and gene loss.
As requested, we have added a sentence to indicate that inferred metagenomes do not allow the study of lateral gene transfer and gene loss unlike shotgun metagenomics. In addition, only archaea, bacteria, and fungi can be studied directly by these tools, unlike shotgun metagenomics which provides an overview of all microbial communities.

Similarly, Line 545 should consider gene deletion in addition to horizontal gene transfer.
As requested, we have completed our sentence to indicate that gene gain and loss was also due to gene duplication, gene loss, and de novo gene birth in addition to predominant mode HGT. We have also added bibliographic references regarding the identification of HGT from shotgun metagenomic data.

Line 552: In addition to plasmid transfer, should consider phage / viruses
As requested, we have added a sentence to complete our paragraph on the transfer of plasmids by phages or viruses.

Line 665: It could be instructive to provide more examples of practical applications
Indeed, we were in the same expectation as reviewer 1 but there are very few examples of practical applications. Moreover, this is what we underline throughout our manuscript. However, as requested, we added two examples in the human health on the search for cancer biomarkers and two examples on the biomonitoring of water quality. We have also added bibliographic references regarding these practical applications.

Line 699: It is not clear why these particular soil measurements are of interest
Yes, we fully agree with the reviewer 1. As requested, we added a sentence to precise the definition of the volatile organic compound in order clear why it is interesting to use these soil measurements. We also added VOC abbreviation in the section Abbreviations.

Figure 1: Change "Functional inferance" to "Functional inference"
As requested, we did the correction in the figure 1.

Figure 2: Add data for most recent years?
Yes, we fully agree, it is not intended and we added data for 2018, 2019 and 2020 years in the figure 2.

Figure 9: Change "Unknow or few data" to "Unknown or insufficient data"
As requested, we did the correction in the figure 9.

Reviewer: 2
Reviewer #2: The authors presented a review about inferring microbiota functions from taxonomic genes. In general, this topic is very important to gain more insights from amplicon based sequencing strategies to decipher the role of the microbiome. The manuscript is clearly written and the authors present nicely the state of current tools.
I have just a couple of minor comments that should be addressed.

First of all, we want to thank the Reviewer 2 for these commentaries and he pinpoints some drawbacks to help us improving it.

The specific corrections, and modifications advised by the Reviewer 2 will be highlighted in Blue in the manuscript.

I wonder whether the author can mention PICRUSt2 earlier in the manuscript as it is a great improvement compared to v1. I would suggest to add one sentence in the section about PICRUSt v1.
As requested, we have added sentence to describe the main improvements of PICRUSt2 following the paragraph of PICRUSt1 (L271).

[L176] @MInter should be spelled correctly.
As requested, we did the correction in the sentence.

[L224] Picrust should be written PICRUSt
As requested, we did the correction in the sentence.

[L421] The author write 'No tool...' but I guess it should be 'The tool...'
Indeed, it is a clerical error. We did the correction in the sentence.

[L508] A space is missing ('...fungi - along with...')
As requested, we added the space.

Close
