# Supplementary material for: Inferring microbiota functions from taxonomic genes: a review
Source: Gigascience. 2022 Jan 12;11:giab090. doi: 10.1093/gigascience/giab090 (PMC8756179; doi:10.1093/gigascience/giab090)
Supplement: giab090_Reviewer_2_Report_Original_Submission — Axel KÃ¼nsnter, PhD -- 11/2/2021 Reviewed [file giab090_reviewer_2_report_original_submission.pdf]

Reviewer Report

Title: Inferring microbiota functions from taxonomic genes: a review

Version: Original Submission **Date:** 11/2/2021

Reviewer name: Axel KÃ¼nsnter, PhD

Reviewer Comments to Author:

The authors presented a review about inferring microbiota functions from taxonomic genes. In general, this topic is very important to gain more insights from amplicon based sequencing strategies to decipher the role of the microbiome. The manuscript is clearly written and the authors present nicely the state of current tools.
I have just a couple of minor comments that should be addressed.
I wonder whether the author can mention PICRUSt2 earlier in the manuscript as it is a great improvement compared to v1. I would suggest to add one sentence in the section about PICRUSt v1.
[L176] @MInter should be spelled correctly.
[L224] Picrust should be written PICRUst
[L421] The author write 'No tool...' but I guess it should be 'The tool...'
[L508] A space is missing ('...fungi - along with...')

Methods

Are the methods appropriate to the aims of the study, are they well described, and are necessary controls included? Choose an item.

Conclusions

Are the conclusions adequately supported by the data shown? Choose an item.

Reporting Standards

Does the manuscript adhere to the journal's guidelines on minimum standards of reporting? Choose an item.

Choose an item.

Statistics

Are you able to assess all statistics in the manuscript, including the appropriateness of statistical tests used? Choose an item.

Quality of Written English

Please indicate the quality of language in the manuscript: Choose an item.

Declaration of Competing Interests

Please complete a declaration of competing interests, considering the following questions:

- Have you in the past five years received reimbursements, fees, funding, or salary from an organisation that may in any way gain or lose financially from the publication of this manuscript, either now or in the future?
- Do you hold any stocks or shares in an organisation that may in any way gain or lose financially from the publication of this manuscript, either now or in the future?
- Do you hold or are you currently applying for any patents relating to the content of the manuscript?
- Have you received reimbursements, fees, funding, or salary from an organization that holds or has applied for patents relating to the content of the manuscript?
- Do you have any other financial competing interests?
- Do you have any non-financial competing interests in relation to this paper?

If you can answer no to all of the above, write 'I declare that I have no competing interests' below. If your reply is yes to any, please give details below.

I declare that I have no competing interests

I agree to the open peer review policy of the journal. I understand that my name will be included on my report to the authors and, if the manuscript is accepted for publication, my named report including any attachments I upload will be posted on the website along with the authors' responses. I agree for my report to be made available under an Open Access Creative Commons CC-BY license (http://creativecommons.org/licenses/by/4.0/). I understand that any comments which I do not wish to be included in my named report can be included as confidential comments to the editors, which will not be published.

Choose an item.

To further support our reviewers, we have joined with Publons, where you can gain additional credit to further highlight your hard work (see: https://publons.com/journal/530/gigascience). On publication of this paper, your review will be automatically added to Publons, you can then choose whether or not to claim your Publons credit. I understand this statement.

Yes Choose an item.
